# Airway and Parenchymal Strains during Bronchoconstriction in the Precision Cut Lung Slice

**DOI:** 10.3389/fphys.2016.00309

**Published:** 2016-07-21

**Authors:** Jonathan E. Hiorns, Cécile M. Bidan, Oliver E. Jensen, Reinoud Gosens, Loes E. M. Kistemaker, Jeffrey J. Fredberg, Jim P. Butler, Ramaswamy Krishnan, Bindi S. Brook

**Affiliations:** ^1^School of Mathematical Sciences, University of NottinghamNottingham, UK; ^2^Laboratoire Interdisciplinaire de Physique, Centre National de la Recherche Scientifique, Université Grenoble AlpesGrenoble, France; ^3^Department of Molecular Pharmacology, University of GroningenGroningen, Netherlands; ^4^Department of Emergency Medicine, Beth Israel Deaconess Medical Center, Harvard Medical SchoolBoston, MA, USA; ^5^School of Mathematics, University of ManchesterManchester, UK; ^6^Department of Environmental Health, Harvard School of Public HealthBoston, MA, USA

**Keywords:** airway smooth muscle, contraction, PCLS, displacements, radial strain, circumferential strain

## Abstract

The precision-cut lung slice (PCLS) is a powerful tool for studying airway reactivity, but biomechanical measurements to date have largely focused on changes in airway caliber. Here we describe an image processing tool that reveals the associated spatio-temporal changes in airway and parenchymal strains. Displacements of sub-regions within the PCLS are tracked in phase-contrast movies acquired after addition of contractile and relaxing drugs. From displacement maps, strains are determined across the entire PCLS or along user-specified directions. In a representative mouse PCLS challenged with 10^−4^M methacholine, as lumen area decreased, compressive circumferential strains were highest in the 50 μm closest to the airway lumen while expansive radial strains were highest in the region 50–100 μm from the lumen. However, at any given distance from the airway the strain distribution varied substantially in the vicinity of neighboring small airways and blood vessels. Upon challenge with the relaxant agonist chloroquine, although most strains disappeared, residual positive strains remained a long time after addition of chloroquine, predominantly in the radial direction. Taken together, these findings establish strain mapping as a new tool to elucidate local dynamic mechanical events within the constricting airway and its supporting parenchyma.

## 1. Introduction

Airway smooth muscle (ASM) cells residing within the airway wall, and the tissue in the surrounding parenchyma, are under constantly changing strains during tidal breathing. It is widely recognized that the effect of imposed strains and resulting stresses, as well as internally generated mechanical force, are of crucial importance in normal physiology and are altered in diseases such as asthma and COPD. However, while complex and inferred in organs and overly simplified in the cultured cell, their generation, transmission, and transduction in the settings of an intact airway remain difficult to measure. Indeed, there are currently no straightforward approaches to quantify the strains or stresses acting on cells and tissues in their native airway microenvironment. In the absence of such knowledge, the mechanical interactions involved in airway (patho)physiology will remain poorly understood.

A well-established experimental preparation for studying airway reactivity, and corresponding biomechanical response, is the precision-cut lung slice (PCLS) (e.g., Perez and Sanderson, 2005; Wang et al., 2008; Tan and Sanderson, 2014). The key advantage of the PCLS is that vital functional interactions between airways, arterioles, and veins are preserved within the alveolar parenchyma (Sanderson, 2011). Additional practical considerations include its ease of preparation, ease of storage via cryopreservation (Rosner et al., 2014; Bai et al., 2016), widespread applicability to many animal species (Seehase et al., 2011) including humans (Wohlsen et al., 2003), and suitability for high-resolution imaging of molecular dynamics (Sanderson, 2011). In the PCLS, responses to electric field stimulation (Schleputz et al., 2011, 2012) and mechanical stretch (Dassow et al., 2010; Lavoie et al., 2012; Davidovich et al., 2013) have also been ascertained, highlighting the physiological relevance of this system.

Biomechanical data from PCLS studies, to date, have largely focused on changes in airway caliber. These datasets, however, contain a rich source of additional dynamic and spatial biomechanical data that heretofore have not been investigated. For example, a limited number of studies have utilized the PCLS to examine the mechanical interdependence between the constricting airway and the surrounding parenchyma (Adler et al., 1998; Brook et al., 2010; Ma et al., 2013). However, beyond the immediate vicinity of the contracting airway, the parenchyma contains other airways and arterioles which may themselves contract or even passively contribute to the effective material properties of surrounding tissues. Accordingly, detailed spatio-temporal maps of tissue deformation are necessary to elucidate the biomechanical aspects of airway-parenchymal interactions and the inherent transmission of force.

Here, we describe the development and implementation of a strain mapping tool that provides spatial and temporal data from PCLS video recordings. In a representative mouse PCLS they revealed heterogeneous strain profiles around distinct structural features that surround the contracting airway. These heterogeneities highlight the possibility of distinct micromechanical environments for resident cells so that cells may in turn respond heterogeneously depending on their location (Bossé et al., 2011). Furthermore, the present analysis technique promises to be highly useful in correlating levels of strain and structural remodeling in the airway and surrounding parenchyma.

## 2. Methods

### 2.1. Precision cut lung slice preparation and contraction experiment

#### 2.1.1. Animals

Homozygous, inbred, specific-pathogen-free breeding colonies of C57Bl/6NTac wild-type mice were obtained from Taconic. Animals were housed conventionally under a 12-h light-dark cycle and received food and water *ad libitum*. All experiments were performed in accordance with the national guidelines and approved by the University of Groningen Committee for Animal Experimentation (DEC5463I and DEC6792A).

#### 2.1.2. Precision-cut lung slices

Mouse PCLS were prepared according to a protocol described previously for guinea pig PCLS (Oenema et al., 2013). Male C57Bl/6 mice (6–8 weeks old) were euthanized by intraperitoneal pentobarbital injection (400 mg/kg, hospital pharmacy, University Medical Center Groningen), after which the lungs were filled with 1.5 mL low melting-point agarose solution (1.5% final concentration (Gerbu Biotechnik GmbH, Wieblingen, Germany) in CaCl2 (0.9mM), MgSO4 (0.4 mM), KCl (2.7 mM), NaCl (58.2 mM), NaH2PO4 (0.6 mM), glucose (8.4 mM), NaHCO3 (13 mM), Hepes (12.6 mM), sodium pyruvate (0.5 mM), glutamine (1 mM), MEM-amino acids mixture (1:50), and MEM-vitamins mixture (1:100), pH = 7.2). The agarose was solidified for 15 min, by placing the lungs on ice and at 4°C. Lungs were harvested and individual lobes were sliced at a thickness of 250 μm in medium composed of CaCl2 (1.8 mM), MgSO4 (0.8 mM), KCl (5.4 mM), NaCl (116.4 mM), NaH2PO4 (1.2 mM), glucose (16.7 mM), NaHCO3 (26.1 mM), Hepes (25.2 mM), pH = 7.2, using a tissue slicer (Compresstome™ VF-300 microtome, Precisionary Instruments, San Jose CA, USA). Thereafter, slices were kept at 37°C in a humidified atmosphere of 5% CO_2_ and washed every 30 min for four times to remove the agarose and cell debris in medium composed of CaCl2 (1.8mM), MgSO4 (0.8 mM), KCl (5.4 mM), NaCl (116.4 mM), NaH2PO4 (1.2 mM), glucose (16.7 mM), NaHCO3 (26.1 mM), Hepes (25.2 mM), sodium pyruvate (1mM), glutamine (2 mM), MEM-amino acids mixture (1:50), MEM-vitamins mixture (1:100,) penicillin (100 U/mL), and streptomycin (100 μg/mL), pH = 7.2.

#### 2.1.3. Contraction studies

The response of lung slices were recorded after addition of the contractile agonist methacholine (MCh; 10^−4^M, ICN Biomedicals, Zoetermeer, Netherlands) at *t*_0_ = 0 s, and then addition of the bitter taste receptor agonist chloroquine (ChQ; 10^−3^M, Sigma-Aldrich, Zwijndrecht, Netherlands) to induce relaxation at *t*_1_ = 600 s (in the presence of MCh). As described previously, a nylon mesh and a metal washer were used to keep the lung slice in place. Bright field images of the lung slices were captured in time-lapse (1 frame per 2 s) with a resolution of 1280 × 960 pxl (1.15 m/pxl) using an inverted microscope (Eclipse, TS100; Nikon). Airway luminal area was quantified using image acquisition software (NIS-elements; Nikon).

### 2.2. Strain and displacement maps using image analysis

This section details the determination of 2-dimensional time-dependent displacement and strain maps from video sequences of mouse PCLS. In order to calculate displacement and strain fields in a given video frame at a given time point, a number of computational algorithms were developed; the overview of the whole method is shown in Figures A-1, A-2 in the Appendix, as well as further details of the algorithms.

#### 2.2.1. Displacement fields

First, the frames were pre-processed with MATLAB to set the length scale in μm and stretch the range of pixel densities so that specific features became more prominent. This pre-processing step gave a list of the frame numbers and a series of images corresponding to adjusted frames. Second, the Farnebäck algorithm (Farneback, 2003) as implemented in C++ / OpenCV was used to calculate an estimate of the displacement vector between an initial (or reference) and final image (the frame of interest) for each of the pixels. Then, strain matrices were determined at equally spaced points chosen across the image. To do so, four displacement vectors around the point of interest and central difference methods were used to calculate derivatives in the horizontal and vertical directions from which the major and minor eigenvectors and eigenvalues of the strain matrix were evaluated. The initial coordinates of the selected points, the components of the displacement vectors, the major and minor strain eigenvalues and the components of the major strain eigenvector were saved to be used in post-processing. This sequence was repeated for each frame of interest.

Finally, displacements and strains were displayed with MATLAB and their value was set to zero where there was no tissue. Displacement plots could either show arrows on a bright field image (initial or final) or display the magnitude of the displacements in color maps. Major (radial) and minor (circumferential) strain eigenvalue distributions were also displayed as color maps.

#### 2.2.2. Determining strain fields from displacement fields

An alternative to plotting displacement fields is to plot strain fields. An advantage of analyzing strains over displacements is that, if there is movement of a lung slice (relative to the camera position) that is not related to the contraction of the airway, the displacement field will be affected, but the strain field will not.

We assume that displacements between two frames are known (as determined previously), where the coordinates are denoted (*X, Y*) in the first image and (*x, y*) in the second image. The deformation gradient tensor is given by

(1) $$\begin{array}{l} F=\left(\begin{array}{cc} \frac{\partial x}{\partial X} & \frac{\partial x}{\partial Y}\\ \frac{\partial y}{\partial X} & \frac{\partial y}{\partial Y} \end{array}\right). \end{array}$$

The Lagrangian strain tensor is defined as E ≡ (C − I)∕2, where C ≡ F^*T*^F is the right Cauchy-Green deformation tensor. Thus,

(2) $$\begin{array}{l} E=\left(\begin{array}{cc} E_{11} & E_{12}\\ E_{12} & E_{22} \end{array}\right)=\left(\begin{array}{cc} {\left(\frac{\partial x}{\partial X}\right)}^{2}+{\left(\frac{\partial y}{\partial X}\right)}^{2}-1 & \frac{\partial x}{\partial X}\frac{\partial x}{\partial Y}+\frac{\partial y}{\partial X}\frac{\partial y}{\partial Y}\\ \frac{\partial x}{\partial X}\frac{\partial x}{\partial Y}+\frac{\partial y}{\partial X}\frac{\partial y}{\partial Y} & {\left(\frac{\partial x}{\partial Y}\right)}^{2}+{\left(\frac{\partial y}{\partial Y}\right)}^{2}-1 \end{array}\right)∕2. \end{array}$$

One way to visualize the strain is to find the eigenvalues and eigenvectors of E so that the magnitude and direction of the principal strains can be plotted. The characteristic polynomial for the tensor is

(3) $$\begin{array}{l} \lambda^{2}-\left(E_{11}+E_{22}\right)\lambda +\left(E_{11}E_{22}-E_{12}^{2}\right)=0, \end{array}$$

with coefficients given by the strain invariants *I*_1_ = *E*_11_+*E*_22_ and $I_{2}=E_{11}E_{22}-E_{12}^{2}$. Solving the characteristic polynomial yields the eigenvalues in terms of the invariants,

(4) $$\begin{array}{l} \lambda^{\pm }=\frac{I_{1}\pm \sqrt{I_{1}^{2}-4I_{2}}}{2}. \end{array}$$

The eigenvalues depend on a combination of the invariants and so are independent of the coordinate system used. Now

(5) $$\begin{array}{l} I_{1}^{2}-4I_{2}={\left(E_{11}-E_{22}\right)}^{2}+4E_{12}^{2}\geq 0, \end{array}$$

so in general there are two real eigenvalues. The only exception is when *E*_11_ = *E*_22_, for which there is a repeated eigenvalue.

To find the eigenvectors the following equation must be solved:

(6) $$\begin{array}{l} \left(\begin{array}{cc} E_{11}-\lambda^{\pm } & E_{12}\\ E_{12} & E_{22}-\lambda^{\pm } \end{array}\right)\left(\begin{array}{c} x\\ y \end{array}\right)=\left(\begin{array}{c} 0\\ 0 \end{array}\right). \end{array}$$

From the first row, the unit eigenvectors satisfy

$$\frac{\left(E_{12},\lambda^{\pm }-E_{11}\right)}{\sqrt{E_{12}^{2}+{\left(\lambda^{\pm }-E_{11}\right)}^{2}}}.$$

The second row provides an equivalent relationship. The eigenvector could equally point in the opposite direction. The sign of the corresponding eigenvalue can be used to determine if the strain is expansive (λ > 0) or compressive (λ < 0). We assumed that for a single contracted airway, a component of the major principal strain points toward, rather than away from, the lumen. In all our results we found that our principal directions were essentially radial and circumferential; here on we therefore refer to these as radial and circumferential directions.

### 2.3. Radial profiles of strain

#### 2.3.1. Strain kymographs

The strain values plotted as maps were averaged along the circumferential direction as a function of the distance to the airway edge. Each frame of the sequence was made binary with the lumen in white (1) and the rest in black (0) and resized so as to match the dimensions of the corresponding strain maps. For each pixel out of the airway (0), the distance to the nearest airway pixel (1) was computed and stored in a matrix of the same dimension as the ones containing the radial and circumferential strain values. The distance/strain couples were then sorted in distance bins of 15 μm and the mean of the corresponding strain values for this bin was calculated. The results were then represented as series of line plots of the mean strain average as a function of the distance to the airway, time being represented by the color code of the lines. Alternatively a form of kymograph was plotted with the strain represented as a function of time on the *x*-axis and the distance to the airway on the *y*-axis; the value of the strain was color-coded at the corresponding coordinates. This graphic representation highlights how the strains were altered during and after airway contraction and relaxation.

#### 2.3.2. Spokes analysis

An alternative to finding strain fields across the whole image was to determine the strains only at a selection of points along vectors normal to the lumen. To do this, the lumen area of airway in the image had to be identified first. An ellipse was fitted to the lumen of the airway in the first frame. Two methods were used to estimate the position of the lumen and the best result was chosen (A-1.2.1 in the Appendix). The area around the airway was split into eight regions, within which we selected seven sets of points radiating out from the lumen; multiple sets of points were used so that the average strain and variability could be determined as a function of distance from the lumen.

The Farnebäck algorithm (A-1.2.2 in the Appendix) was used to determine the displacements in the radial and circumferential directions at each point and these values were averaged within each of the sections for each radial position, in order to remove small errors. The coordinates of four neighboring points were then determined for each of the points on the spokes. These were selected based on being a specific distance away in the *x* or *y* direction. However, the coordinates of these surrounding points were unlikely to be at integer values of pixels, in which case bilinear interpolation of the displacement of the four closest pixels was used first to calculate estimates for the displacements at the surrounding points, then to derive the strain matrix (using central difference methods to calculate derivatives) and finally the mean of the radial and circumferential strain eigenvalues in each section. MATLAB was used to plot graphs of the section-averaged strain as a function of distance from the lumen, which could also be used to show how the time-dependent distribution of average strain alters as the airway contracts.

## 3. Results

### 3.1. Strain maps show global spatio-temporal influence of airway smooth muscle contraction on the airway wall and parenchyma

**Figure 1:**
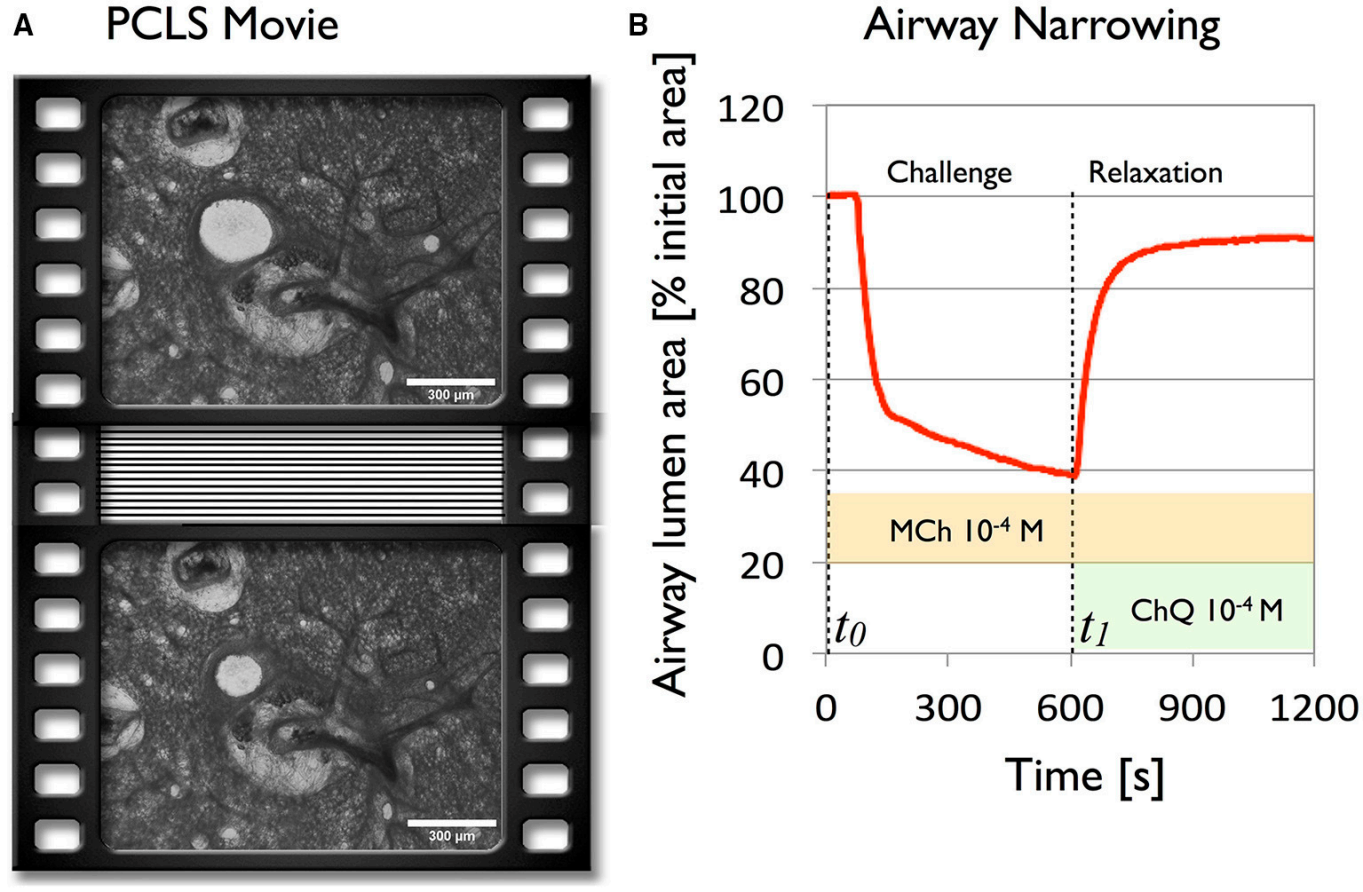
**Global analysis of the deformations of a 250 μm mouse lung slice during agonist driven contraction with methacholine (10^**−**4^M) and subsequent bitter taste receptor agonist relaxation with chloroquine (10^**−3**^M), reveals the global behavior of the parenchyma. (A)** Two frames of a phase contrast PCLS movie selected before (*t*_0_ = 0s) and after contraction (*t*_1_ = 600s). **(B)** Airway caliber plotted as a function of time during contraction and relaxation.

Bronchoconstriction in response to 10^−4^M methacholine (MCh), followed by relaxation in response to 10^−3^M Chloroquine (ChQ) (in the presence of MCh) were recorded by phase contrast microscopy with example images before and at maximal contraction shown in Figure 1A. The time-dependent change in cross sectional area of the airway (Figure B) matched previously measured airway profile changes (e.g., Bergner and Sanderson, 2002; Wang et al., 2008), demonstrating features previously pointed out by Bergner and Sanderson (2002) with an initial steep phase of fast narrowing, followed by a slower, asymptotic phase. We note that in this particular strain of mice, maximal MCh-induced airway narrowing, as measured by luminal area, was achieved only on addition of 1mM methacholine, although airway closure is nearly maximal at 0.1 mM (Figure S1) in Supplementary Material). Addition of ChQ in the presence of MCh induced complete bronchodilation in a series of experiments as shown in Figure S2 (Supplementary Material).

**Figure 2:**
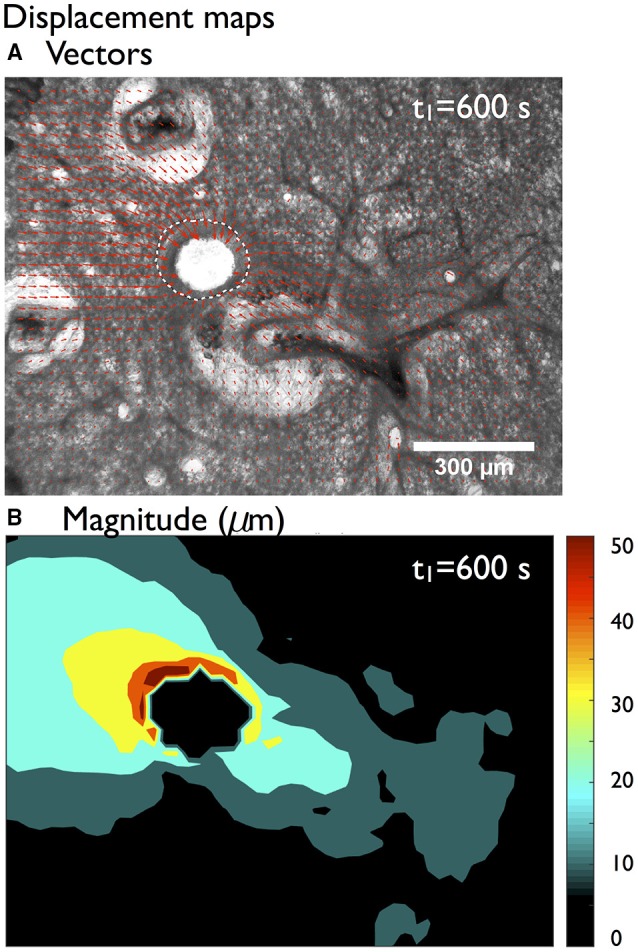
**Global analysis of the deformations of the mouse lung slice in Figure 1**. Displacement **(A)** vectors and **(B)** magnitude of small regions (7 × 7 pixels) of the slice computed between the reference (at *t*_0_) and the contracted state (at *t*_1_). The boundary of the airway before contraction is represented as a white dashed line. See Movies M1, M2 (and Figures S3–S5 for analyses of additional PCLS) in Supplementary Material.

Local displacement of small regions (7 × 7 pixels), computed with respect to a reference image (at *t*_0_ right before the challenge) are displayed using displacement vectors in Figure 2A 10 min after MCh challenge. These are overlaid on top of the image of the contracted airway, with the boundary of the airway before contraction represented as a white dotted line. As expected, these displacement vectors are oriented toward the center of the lumen. A map of displacement magnitude over the entire PCLS (Figure 2B) indicates, however, that although the largest displacements are in or near the airway wall, there are significant non-zero displacements almost two airway-diameter lengths away from the airway wall (to the left of the airway in (Figure 2B).

**Figure 3:**
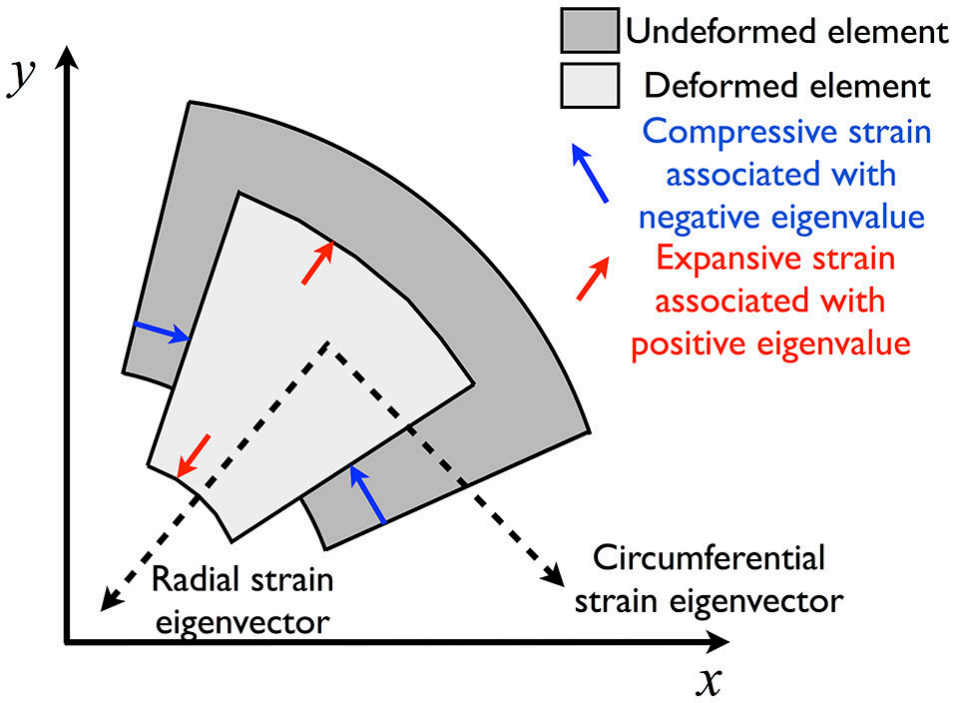
**Schematic illustrating effect of contraction on an element of tissue in the PCLS**. Strains are decomposed into radial and circumferential components associated with eigenvectors that essentially point in the radial and circumferential directions. Determination of these eigenvector directions allows the deformation to be described predominantly as expansion and compression with minimal shear (diagonal elements in the strain tensor, *E*_11_ and *E*_22_ in (2), dominate over the off-diagonal elements, *E*_12_).

**Figure 4:**
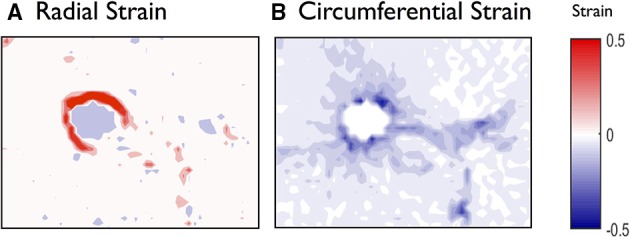
**Global analysis of the deformations of the mouse lung slice in Figure 1**. **(A)** Radial (major) and **(B)** circumferential (minor) strains calculated by spatial derivation of the displacements and displayed as maps over the whole field. See Movie M3 (and Figures S3–S5 for analyses of additional PCLS) in Supplemental Material.

**Figure 5:**
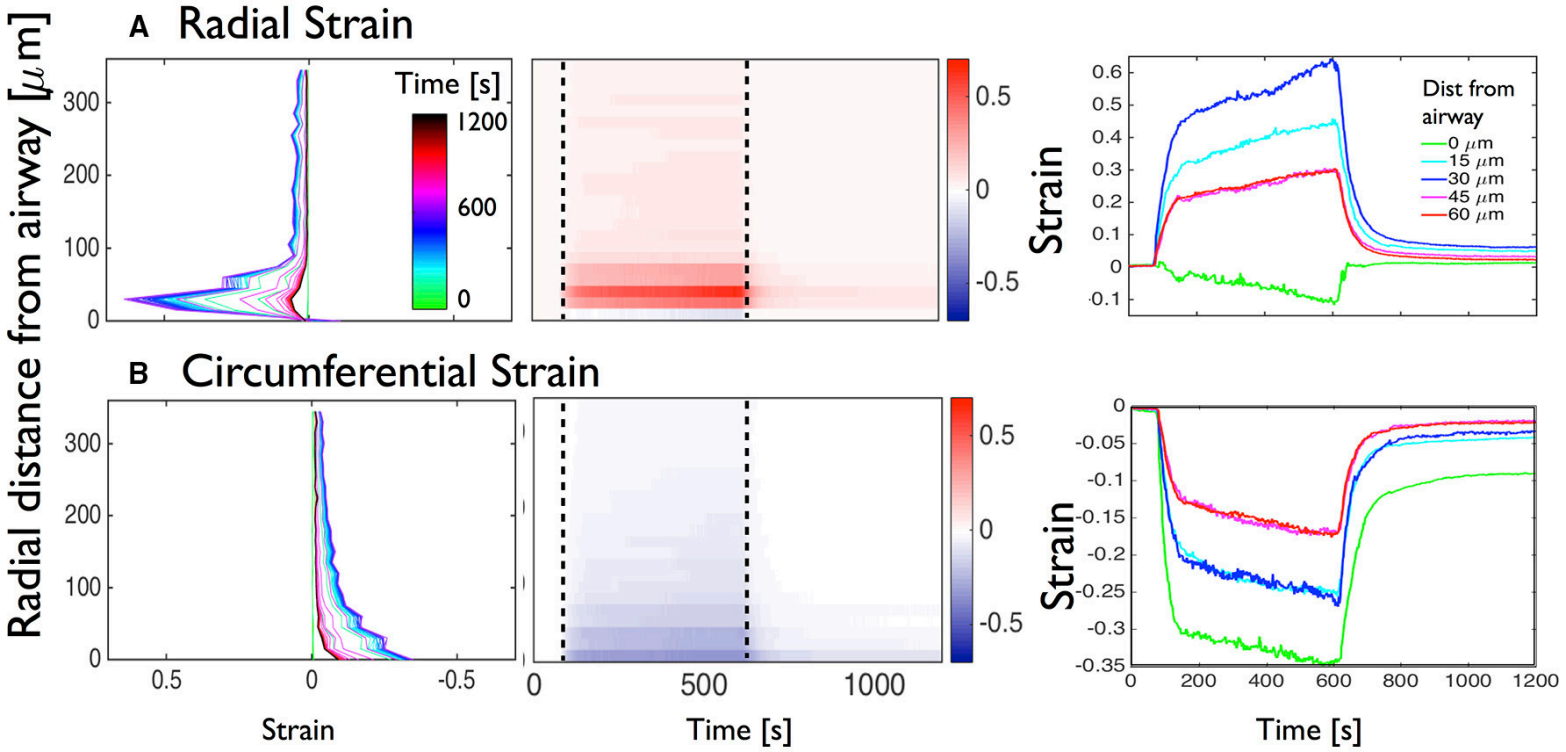
**Global analysis of the deformations of the mouse lung slice in Figure 1**. Temporal evolution of **(A)** radial and **(B)** circumferential strains as a function of distance from the airway. Left column: superimposition of distance-strain line plots for increasing time as indicated by the colorbar (inset on top figure of left column). Middle column: adapted kymographs showing magnitude of strain, as indicated by the colorbar to the right of each figure. Right column: superimposition of line plots showing temporal evolution of strain at 0, 15, 30, 45, and 60 μm from the airway lumen.

Tissue displacements, observed in Figure 2B, are determined for the whole image and normalized to obtain strain maps. The major and minor strains approximately represent the radial and circumferential strains respectively (Figure 3). We observe that their spatial distributions are clearly quantitatively and qualitatively different (Figure 4). In particular we note that the deformations in the radial directions are essentially stretches (Figure 4A; positive major strains) whereas the deformations in the circumferential directions are largely compressive (Figure 4B; negative minor strains). In both cases, the largest deformations are found along the airway wall, but hot spots of strains are also present in the parenchyma. Figure 5 (right) highlights the heterogeneous distribution of the deformations over the parenchyma surrounding the airway, with some regions being dominated by extension/stretch and others by compression.

To visualize the temporal evolution of strain distribution, the displacements and strain maps were computed for each frame of the 20 min contraction and relaxation movie, with respect to the reference image at *t*_0_ (see Movies in Supplementary Materials). For each time point, major and minor strains are averaged circumferentially over pixels that are radially equidistant (at 15 μm intervals) from the airway wall and plotted along the radial direction for each time point and superimposed on Figure 5 (left column). Again, the peaks of strain are found in the 100 μm region closest to the airway wall, with the radial strain being mainly positive (expansive) and the circumferential strain negative (compressive). The strain profiles however, show that compression dominates in the 50 μm closest to the airway lumen whereas stretch dominates between 50 and 100 μm away from the lumen edge. The color code used to represent the time indicates that in all cases, the strain magnitudes progressively increase until the addition of relaxant at *t*_1_ = 600 s.

To better visualize the evolution of the strain profiles, the data are represented as kymographs [Figure 5 (middle column)]. We observe that at 90s, after the contraction starts, both radial and circumferential strains over the entire PCLS indicate that the large deformations observed in the vicinity of the airway wall propagate further away. Aligning the 2D plots with the standard contraction curve (Figure 1B) enables us to (i) correlate the lag time with the absence of deformation, (ii) correlate the early phase of fast narrowing with the rapid appearance of deformations in the 100 μm closest to the airway lumen, (iii) observe the slower asymptotic phase of contraction from 400 to 600 s and (iv) correlate the rapid attenuation of the majority of strain with addition of relaxant added after 600 s.

Plotting the strains at specific distances from the lumen as a function of time [Figure 5 (right column)], we observe that there is some compressive radial strain at the lumen [green curve; Figure 5A (right column)] which is not visible in the left panel. Additionally we observe that although the radial strains return to zero at the lumen upon addition of ChQ (green curve, 0 μm), the regions further away from the lumen (blue curve, 30 μm) retain a residual positive major strain a long time after relaxant (*t* = 1200s) was added, suggesting some longer term structural changes. Furthermore, the circumferential strain remained significantly compressive at the lumen, and to a lesser extent further away from the lumen, at *t* = 1200s.

### 3.2. Spokes analysis reveals the influence of structural heterogeneities on strain distribution in the airway wall and parenchyma

**Figure 6:**
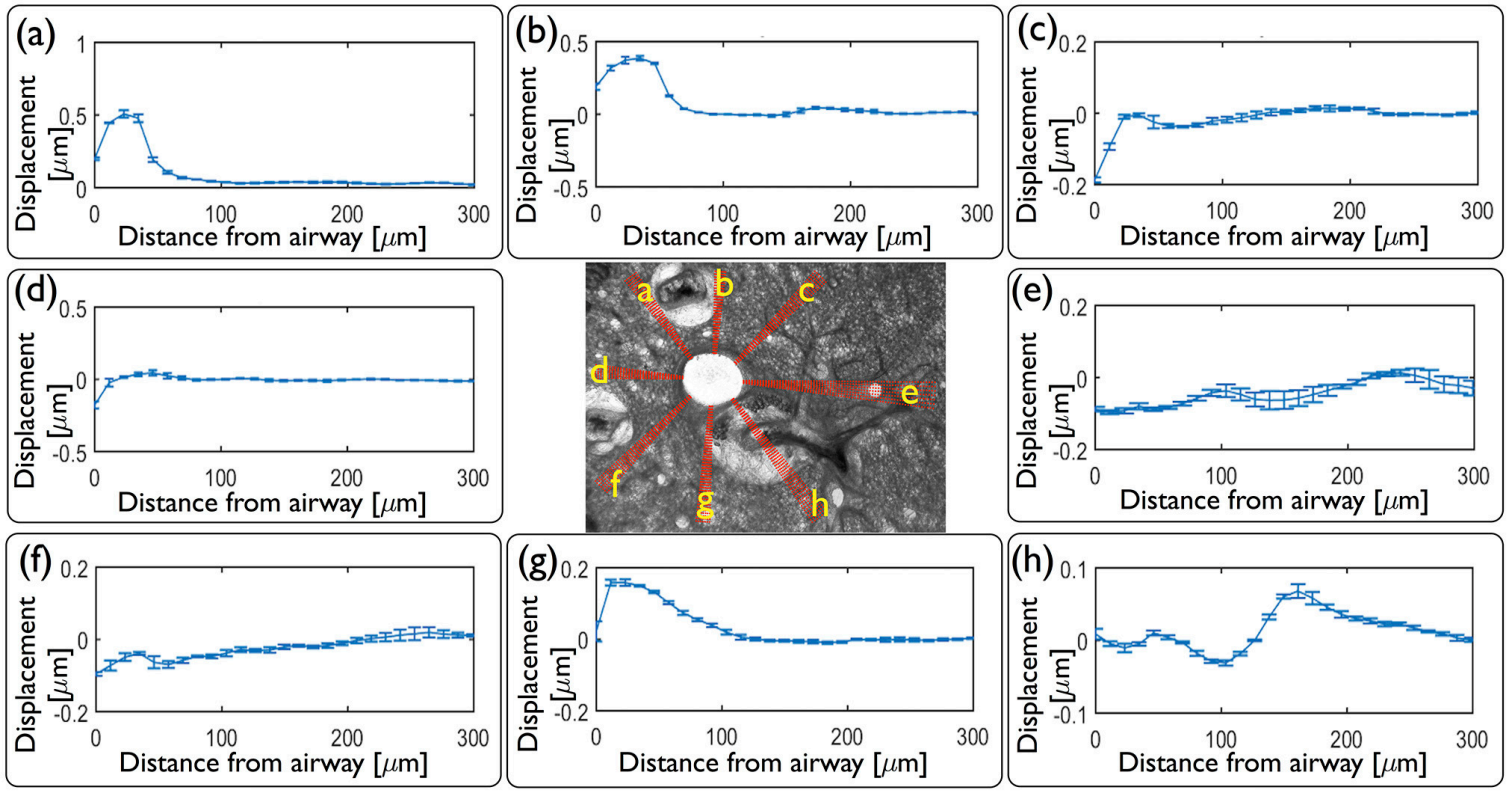
**Local quantitative analysis of the mouse lung slice (central image) from Figure 1 at peak contraction following application of agonist**. Inward radial displacements are plotted as a function of distance from the airway lumen for each of the eight sets of independent spokes **(a** – **h)** shown on the central image. Spokes **a**, **b**, and **g** go through the highly collagenous part on the edge of a blood vessel; spokes **c**, **d**, and **f** go through alveolar tissue, spoke **e** intersects another small contractile airway and **h** goes through a blood vessel.

As an alternative to computing the displacements and strains across the whole field, we compute displacements and strains along eight sets of spokes normal to the airway wall [Figure 6 (center)]. Displacements averaged over each set of spokes at maximum contraction, at *t*_1_ (Figures 6a–h), reveal the heterogeneity observed in Figure 2. Within each set of spokes, we observe very small variability (as indicated by the error bars on each line plot in Figure 6a–h) but significantly different displacement profiles around the airway.

**Figure 7:**
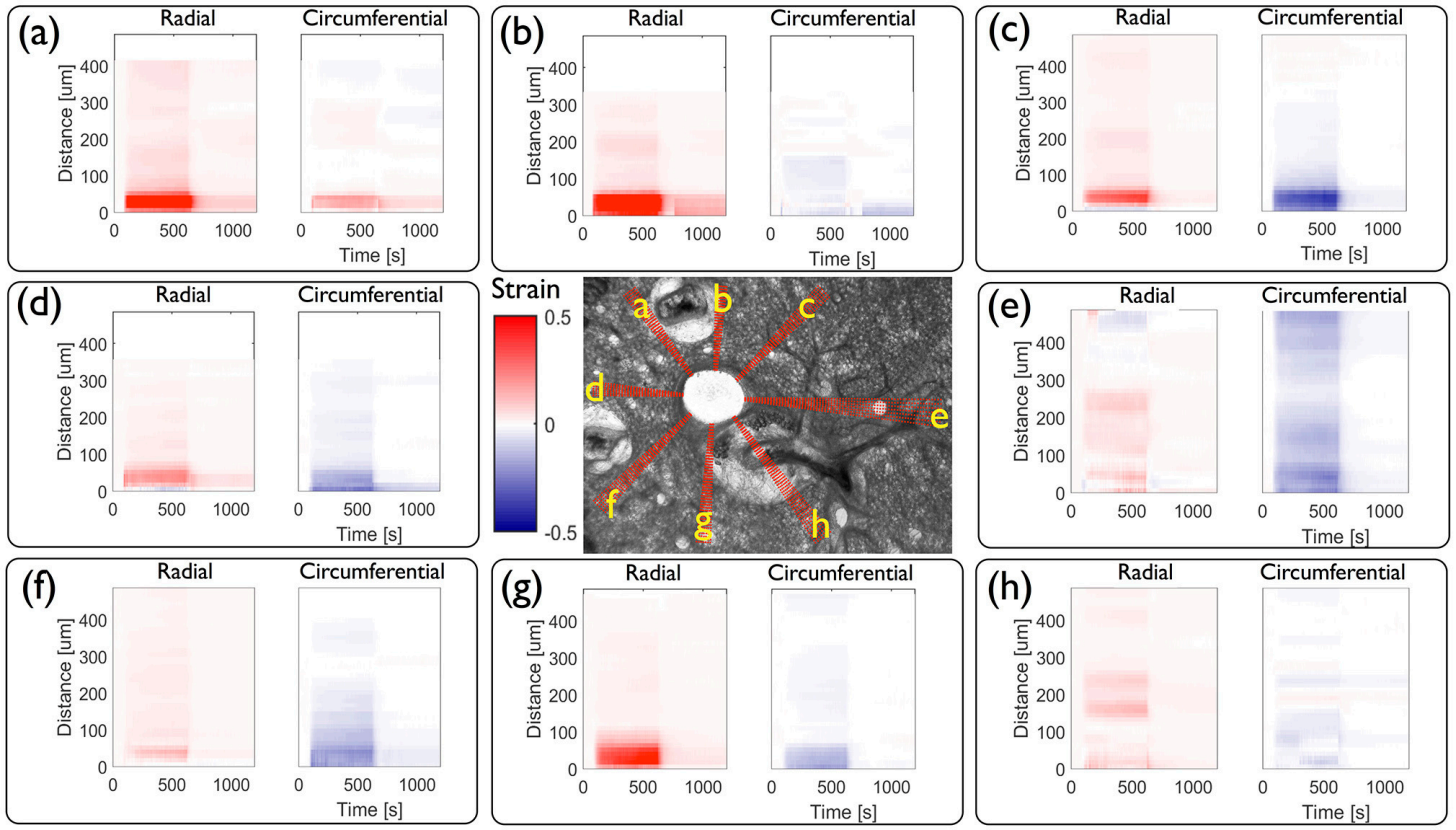
**Local quantitative analysis of a mouse lung slice (central image) from Figure 1** during agonist driven contraction. Radial and circumferential strain kymographs are plotted as a function of both time and distance from the airway lumen for each of the eight sets of independent spokes **(a** – **h)** shown on the central image. Spokes **a**, **b**, and **g** go through the highly collagenous part on the edge of a blood vessel; spokes **c**, **d**, and **f** go through alveolar tissue, spoke **e** intersects another small contractile airway and **h** goes through a blood vessel.

From these displacements we determine the time evolution of radial and circumferential strain profiles in all directions and represent them as kymographs in Figure 7. As the parenchymal tissue is structurally heterogeneous, the spokes selected around the airway intersect different structural features and hence display different strain profiles. While most of these features are physiological, some of them are modified during the slicing procedure. For instance, blood vessels are known to contract strongly in response to the slicing, disrupting the rather weak connective tissue tethering the blood vessel to the parenchyma, leaving behind spaces that appear to be filled by agarose. The strains along three spokes going through the agarose surrounding the blood vessels (a, b, g) show high positive radial strains characteristic of large stretch in the close vicinity of the airway lumen. Three spokes that intersect only alveolar tissue (c, d, f), display roughly similar magnitudes of radial and circumferential strains during the entire contraction event. The spoke (e) intersects another smaller contractile airway, which greatly affects the corresponding strain profile in spatially distributing the deformations between the main and the secondary airway, with a slight domination of compression which persists as far as 400 μm from the lumen. In contrast, the spoke (h) passes through an adjoining blood vessel surrounded by agarose, which also smooths the strain profile. During the relaxation phase, most of the strains disappear, except in the spokes (a, b, g). Along these, one can observe residual positive strains, predominantly in the radial direction. This suggests that the circumferentially averaged positive residual strains observed above [Figure 5 (right column)] can be attributed specifically to positive residual strains in this region of the tissue. Taken together, the strain profiles show that the extent to which strain is transmitted from the contractile airway toward the parenchyma depends highly on the structural heterogeneities present around the airway (be they physiological or experimentally-induced).

## 4. Discussion

To date, most studies using PCLS have simply monitored airway caliber. Although, a few studies have extracted some detailed strain data from PCLS (Adler et al., 1998; Brook et al., 2010; Ma et al., 2013), these have been obtained by tracking specific landmarks in the tissue. In this study, by contrast, we present a computational strain-mapping tool that is able to characterize heretofore inaccessible mechanical events that bear directly upon the physiology of airway narrowing. In a representative mouse PCLS, we illustrate how a variety of displacement and strain measures can be visualized dynamically and quantitatively in both the contracting airway and the surrounding parenchymal tissue. Displacements of sub-regions of the slice are tracked on the phase contrast movies acquired after addition of contractile and/or relaxing drugs to generate maps of displacement across the whole slice. Sequences of strain maps or maps of normalized deformations are then derived from the displacement maps. With our computational strain-mapping tool, we provide access to the detailed mechanical response data in PCLS in the whole airway-parenchymal tissue both globally and also along local user-specified directions. The strain maps give an overview of the deformations imposed by ASM contraction on the airway wall, the tethers and the alveolar tissue. At maximum contraction, both radial and circumferential strains are higher in the airway wall and on the tethers. However, the maps reveal that these deformations are partly transmitted through the slice and that their distribution in the parenchymal tissue is highly heterogeneous. Strain data are thus treated at two different scales so as to derive global and local behaviors of the tissues in response to ASM contraction.

We first extracted the global behavior of the radial and circumferential strain profiles as a function of both time and space (Figure 4). In the present representative mouse slice, the maximum deformation appears at the airway lumen, where the ASM is located (due to contraction, triggered by methacholine), about 1 min after addition of the contractile agonist, and essentially manifests as a radial expansion and a circumferential compression. In the radial direction, a sharp drop in strain is observed, starting from 120 μm away from the airway lumen, but the non-zero strain values observed at larger distances from the lumen indicate that deformations are partially transmitted to the parenchymal tissue during bronchoconstriction (Figure 5). After addition of the bitter taste receptor agonist, chloroquine, to relax the ASM cells, the small strains quickly disappear in the parenchyma but a residual radial stretch remains in the ASM even after 10 min. This sustained mechanical response is completely missed if only the airway caliber is measured.

We also extracted the local displacement (Figure 6) and strain profiles as a function of time and space (Figure 7) in order to investigate the heterogeneities revealed by the strain maps. These heterogeneous patterns are likely to be linked to the mechanical and structural heterogeneities of the underlying tissue. Indeed, stiffer tissue is subject to relatively small deformations, relatively high stresses and transmits the force generated by the contractile ASM, whereas softer tissue is subject to large deformations and cannot transmit the same levels of force. Furthermore, other contractile airways in the neighborhood of the airway of interest affect the strain distribution as they contribute to additional load and stiffer tissue. This structural aspect is striking in this representative mouse slice (Figure 7), where three blood vessels and a smaller contractile airway surround a large bronchial airway. Strain profiles computed in spokes that traverse these particular features of the tissue, show very different behavior. It is also possible that the strain profile depends on a possible heterogeneous distribution of ASM bundles around the airway lumen; the larger strains observed in the upper left part of the tissue adjacent to the airway may be due to larger amounts of ASM there than in the lower part of the airway. In any case these heterogeneous strain profiles [that emerge from the integrative response of both force generation and locally variable stiffnesses (Hiorns et al., 2016)] are likely to provide distinct micromechanical environments for resident cells that may in turn respond heterogeneously depending on their location (Bossé et al., 2011).

As with many image analysis methods, robust mechanical studies on PCLS require samples and contraction experiments of high quality. Therefore, strain map users have to be aware of the limitations associated with both PCLS harvesting and image acquisition during contraction experiments when interpreting the results. For example, the vascular smooth muscle in blood vessels are known to spontaneously contract before the slicing process, which causes disruption of tethers connecting blood vessels to surrounding parenchymal tissue which show up in the image as large white areas filled with agarose (Figure 2A). Agarose being relatively stiff compared to the rest of the alveolar tissue, the positive major strains (predominantly stretch in the radial direction) indicate that tissue is rather squeezed between the airway wall and the edge of the large agarose area, whereas the negative minor strains (compression in the circumferential direction) are also observed in the center of the collapsed blood vessels (Figure 7). The artificial presence of agarose around the blood vessels in the tissue thus generates strain patterns that are likely not physiologically relevant *in vivo*. Injection of gelatin into the vasculature during lung harvesting may prevent this phenomenon (Perez and Sanderson, 2005; Wang et al., 2008). Additionally the presence of agarose in the parenchymal spaces will contribute viscoelastic components not ordinarily present *in vivo* (Brook et al., 2010; Ma et al., 2013) thus modifying effective mechanical properties and dynamic response of the parenchymal tissue. It is also vitally important to ensure that the edges of the PCLS during the contraction experiment are held down to prevent sliding of the slice and therefore control the boundary conditions of the system. This is currently done with a mesh and a washer. Acquiring the contraction movie with high resolution and low magnification is preferable in order to capture enough of the parenchymal tissue surrounding the airway of interest. Although, strain maps can be derived from any set of contraction images as illustrated in additional examples in the Supplementary Material (Figures S3–S5) the significant structural variability seen in all the PCLS has precluded the derivation of a single global metric that can capture the different strain distributions observed around just one airway. Finally, our approach for image analysis was developed and validated specifically on bright-field images. In future, we intend to expand its use to phase contrast images that have significantly higher contrast and increased clarity.

The mechanisms of bronchodilator-induced airway dilation, including the intracellular signaling events that these substances activate in the ASM cells or lung tissue, are likely to vary between each class of bronchodilator and are different to those that cause airway dilation due to bronchoconstrictor degradation (e.g., by esterases in the tissue) or withdrawal. However, our primary aim was to demonstrate how our computational tool allows us to assess residual strains after a full cycle of constriction and dilation, regardless of the underlying chemical pathways that have induced them. Indeed these data remain to be verified more broadly with other bronchodilator pathways in future studies.

Methods for determination of local tissue distortions have been previously developed by Malcolm et al. (2002) and used in some PCLS studies (e.g., Adler et al., 1998; Brook et al., 2010; Ma et al., 2013). This technique, mentioned above, requires identification of visually obvious anatomical landmarks around the image, the changing positions of which are then tracked through the sequence of images until contraction is complete; displacement vectors are then determined between the start and end positions of the landmark. The technique we have exploited and further developed, however, is able to determine the displacement vectors and strain fields over the entire image without need to select landmarks, allowing for more systematic interrogation of the underlying data (such as through the spokes analysis we have developed). A similar strain-mapping technique was used by West et al. (2013) to characterize strains in a tissue-engineered ASM.

We also expect this strain-mapping tool to have application in other PCLS studies aimed at understanding airway mechanics. For example, Lavoie et al. (2012) addressed the role of transpulmonary pressure variations on bronchoconstriction by adapting cell mapping rheometry for use with PCLS. Such studies can benefit from strain mapping; first to calibrate the stretch device through a precise measurement of the strains imposed on the soft substrate; then to quantify the deformations of the PCLS in response to those strains. The predictive capabilities of computational models, developed to understand airway tissue mechanics (e.g., Hiorns et al., 2014) and airway-parenchymal interdependence (Ma et al., 2013), can be further enhanced by quantitative validation using additional data provided by the strain-mapping method.

Further work is required to investigate whether residual strains observed are due to sustained mechanical change or length adaptation. If present *in vivo*, this is likely to trigger mechanotransduction pathways responsible for longer term modification of cellular and extracellular properties as well as structural changes termed airway remodeling (Noble et al., 2014). Such remodeling of the ASM compartment is a hallmark of lung diseases such as asthma (James et al., 2009; Brightling et al., 2012; Gosens and Grainge, 2015), and COPD (Bidan et al., 2015). When combined with biological markers of remodeling (such as contraction-driven activation of TGF-β (Tatler et al., 2011; Oenema et al., 2013), the present analysis technique promises to be highly useful in correlating levels of deformations and remodeling in the airway and surrounding parenchyma. Internal stresses in response to tissue strains, which are experimentally inaccessible but can be predicted using validated models (Hiorns et al., 2016), will play an important role in understanding the nature of the micromechanical environment *in vivo*.

Many lung diseases such as asthma and COPD are characterized by airway hyper-responsiveness and structural changes in the airway (remodeling) or the parenchymal tissue (emphysema). We believe the strain-mapping tool we have developed could enable characterization of the mechanical aspects of such pathophysiology in human PCLS. The evident wide use (Held et al., 1999; Wohlsen et al., 2003; Bai and Sanderson, 2006; Dassow et al., 2010; Sanderson, 2011; Schleputz et al., 2011, 2012; Seehase et al., 2011; Lavoie et al., 2012; Davidovich et al., 2013), and need to characterize the mechanics of airway tissue, (Adler et al., 1998; Brook et al., 2010; Ma et al., 2013) suggests that making the strain mapping computational tool widely available will benefit researchers within the ASM, asthma and COPD communities. Moreover, the method proposed in this work can be easily adapted to any other type of precision cut slices focusing on contracting hollow organs like the gut, bladder, uterus, or the vascular system, and the associated pathologies related to their contractile behavior.

## Author contributions

JEH, CMB: Conception and design; acquisition of experimental data; analysis and interpretation; drafting and revising the manuscript for important intellectual content; final approval of version to be published. OEJ, JJF, JPB, RK, BSB: Conception and design; interpretation; revising the manuscript for important intellectual content; final approval of version to be published. RG: experimental design; interpretation; revising the manuscript for important intellectual content. LEMK: acquisition of experimental data; interpretation; revising the manuscript for important intellectual content.

## Funding

JEH was supported by the Medical Research Council (MRC) Capacity Building Studentship scheme (G0900197). BSB was supported by a New Investigator Research Grant funded by the MRC (G0901174).

### Conflict of interest statement

The authors declare that the research was conducted in the absence of any commercial or financial relationships that could be construed as a potential conflict of interest.
