# Corrigendum: Airway and Parenchymal Strains during Bronchoconstriction in the Precision Cut Lung Slice

**DOI:** 10.3389/fphys.2017.00117

**Published:** 2017-02-28

**Authors:** Jonathan E. Hiorns, Cécile M. Bidan, Oliver E. Jensen, Reinoud Gosens, Loes E. M. Kistemaker, Jeffrey J. Fredberg, Jim P. Butler, Ramaswamy Krishnan, Bindi S. Brook

**Affiliations:** ^1^School of Mathematical Sciences, University of NottinghamNottingham, UK; ^2^Laboratoire Interdisciplinaire de Physique, Centre National de la Recherche Scientifique, Université Grenoble AlpesGrenoble, France; ^3^Department of Molecular Pharmacology, University of GroningenGroningen, Netherlands; ^4^Department of Emergency Medicine, Beth Israel Deaconess Medical Center, Harvard Medical SchoolBoston, MA, USA; ^5^School of Mathematics, University of ManchesterManchester, UK; ^6^Department of Environmental Health, Harvard School of Public HealthBoston, MA, USA

**Keywords:** airway smooth muscle, contraction, PCLS, displacements, radial strain, circumferential strain

In the original article, we neglected to thank our funder MRC, MR/M004643/1 to BB. The authors apologize for this error and state that this does not change the scientific conclusions of the article in any way.

## Conflict of interest statement

The authors declare that the research was conducted in the absence of any commercial or financial relationships that could be construed as a potential conflict of interest.

