# Supplementary material for: Airway and Parenchymal Strains during Bronchoconstriction in the Precision Cut Lung Slice
Source: Front Physiol. 2016 Jul 21;7:309. doi: 10.3389/fphys.2016.00309 (PMC4989902; doi:10.3389/fphys.2016.00309)
Supplement: Supplementary file 4 [file Presentation1.zip › StrainMap_package_21-08/Strain_Map/How_to_setup_cpp.rtf]

How to install C++ to run Jonathan's codes
Install files for Windows7 Pro 64-bit can be found in …\Strain_Map\install 
1.	Install Code::Blocks with the (MinGW) C++ compiler option.
2.	Add “C:\Program Files\CodeBlocks\MinGW\bin” to system path 
Note1: if you don't know how to change the path
http://www.computerhope.com/issues/ch000549.htm
Note2: adapt your name folder in the following if CodeBlocks is installed in Program Files (x86)
3.	Install Cmake 2.8
4.	Extract “OpenCV-2.2.0-win.zip” to “C:\OpenCV-2.2.0-win” (It creates a second folder so the final destination looks like that: “C:\OpenCV-2.2.0-win\OpenCV-2.2.0″)
5.	Run Cmake (cmake-gui)
6.	Set the source code: “C:\OpenCV-2.2.0-win\OpenCV-2.2.0″
7.	Set where to build the binaries: e.g. “C:\OpenCV2.2.0MinGW”
8.	Press Configure
9.	Let Cmake create the new folder
10.	Specify the generator?: MinGW Makefiles
11.	Select “Specify native compilers” and click next
12.	For C set: C:/Program Files/CodeBlocks/MinGW/bin/gcc.exe
Note: adapt your name folder in the following if CodeBlocks is installed in Program Files (x86)
13.	For C++ set: C:/Program Files/CodeBlocks/MinGW/bin/g++.exe
Note: adapt your name folder in the following if CodeBlocks is installed in Program Files (x86)
14.	Click finish
15.	In the configuration screen type in “RELEASE” for “CMAKE_BUILD_TYPE” and select BUILD_EXAMPLES
16.	Click Configure again
17.	Clicked Generate
18.	Closed Cmake
19.	Go to the command prompt (Start>All Programs>Accessories) - type cd OpenCV2.2.0MinGW and hit enter to access the folder “C:\OpenCV2.2.0MinGW” - type “mingw32-make” and hit enter (takes some time, black window with green fonts)
20.	Then type “mingw32-make install” and hit enter again
21. Add “C:\OpenCV2.2.0MinGW\bin” to the system path
22. Open CodeBlocks and use it to open C and C++ files by default
Source: http://conanhung.wordpress.com/2012/05/23/opencv-2-2c-codeblocks-and-mingw-got-it-working-on-windows/
