# Supplementary material for: Airway and Parenchymal Strains during Bronchoconstriction in the Precision Cut Lung Slice
Source: Front Physiol. 2016 Jul 21;7:309. doi: 10.3389/fphys.2016.00309 (PMC4989902; doi:10.3389/fphys.2016.00309)

How to use Jonathan’s codes to compute strain maps?

Updated by Cecile on 14/08/15

Organization of the folders and files

In Strain_Map (where you want in your computer, but don’t move it afterwards)

└ contains [1 Folder per slice (ex: A1_c1_01) + ‘BlackColormaps’ + ‘RedBlueColormaps’]

└ contains [1 Folder ‘Strain Files’ + Exp images (frame*4digits*.tif)]

└ contains [Lungslice, main_strain_calc.cpp, main_strain_calc_spokes.cpp (CodeBlocks, C++)
post_strain_calc.m, post_strain_calc_spokes.m, pre_strain_calc.m (Matlab)] - Copy the folder for each new set of data because the codes all need parameters specific to the slice analyzed, so you don’t want to modify them each time.

1/ In Matlab, open **A_pre_strain_calc.m**, adjust the section “VARIABLES TO BE ADJUSTED TO THE SET OF DATA”: you will specify the right ‘current folder’ to work in, give in the number of the first and last frames, the folder and the ‘pixelscale’ (um/pxl)

Run the code, draw a rectangle around the airway (close enough) and choose the best method that fits the lumen (1 or 2).

At the end, adjusted frames (.tif) and the parameters of the ellipse fitted to the lumen (.txt) are saved in the folder.

To get regular strain maps

2/ Open **B_Lungslice_strain_maps** project with Code:Blocks. In the C++ code *main_strain_calc.cpp* (in source), adjust (copy/paste from ellipse.txt): the folder, the number of the frames in ‘frame vector’, the length of the frame vector and the ‘pixelscale’ (um/pxl)

Hit ‘build and run’, strain data between the 1st frame and the current frame are saved in the folder.

Note: if you have a big set of data to analyze the computer might run out of memory. The trick is to make 2 batches of calculations. You just need to keep the same reference image (the 1^st^ frame number in framevec remains the same). Don’t forget to adjust the total number of frames on the *framevec* and *lengthframevec* lines (24 and 25).

3/ In Matlab, open **C_post_strain_calc.m**, specify the right ‘current folder’ and adjust some parameters in the code: the folder, the number of the frames analyzed (copy/paste from ellipse.txt is faster) and the time interval between 2 frames, max_disp and max_strain set the max values for the color scales of the maps.

Run the code, draw a rectangle around the airway and choose the best method to fit the lumen (1 or 2). At the end, images with the displacements vector, figures with the displacement maps and strain maps are saved in the folder (.tif).

**BEFORE PURSUING, CHECK THE EVOLUTION OF THE DISPLACEMENT VECTORS !!!** For contraction movies with high resolution and/or large contraction, the c++ code may have troubles to track the displacements along the airway, resulting in aberrant values of displacements and strains.

General Troubleshooting.

Changing lines 34 *poly_n* and 35 *poly_sigma*, from *5 and 1.1* to *7 and 1.5* may help to make the code more robust (less precise though)

FYI: the values in the package are 7 and 1.5 for images of 1280 x 960 pxl resolution.

For more details, check:

<http://docs.opencv.org/3.0-beta/modules/video/doc/motion_analysis_and_object_tracking.html#void%20calcOpticalFlowFarneback%28InputArray%20prev%2c%20InputArray%20next%2c%20InputOutputArray%20flow%2c%20double%20pyr_scale%2c%20int%20levels%2c%20int%20winsize%2c%20int%20iterations%2c%20int%20poly_n%2c%20double%20poly_sigma%2c%20int%20flags%29>

4/ Open **D_post_strainVSdistance.m**, specify the right ‘current folder’ and adjust some parameters in the code: the folder, the number of the frames analyzed (copy/paste from ellipse.txt is faster), the time interval between 2 frames and the bin size and maximum distance to the lumen and maximum strain wished to plot the strain profiles.

Run the code, check if the contrast will be good enough to track the boundary of the lumen (reference for the strain profiles). If yes, click in the center of the lumen, if not, you will have to draw manually the boundary on each frame. At the end, the evolution of the strain profiles is represented in line plots and as kymographs (.tif).

Note: to modify the aspect ratio of the graphs and/or the way they are plot, modify the settings in the %% Plot strains section.

To get strain analysis on spokes

2/ Open **E_Lungslice_strain_spokes** project with Code:Blocks. In the C++ code *main_strain_calc_spokes.cpp* :

-turn setup to 1 on line 13 to display an image with the initial points. The green spoke is Spoke1 and there are ordered counterclockwise (see the sketch below).
-adjust (copy/paste from ellipse.txt): the folder, the number of the frames in ‘frame vector’, the length of the frame vector, the ‘pixelscale’ (um/pxl), the parameters saved in ellipse.txt (major, minor, orientation, center, edge)
-adjust the values in ‘edge’ to put the initial spokes at the edge of the lumen (not necessarily on the fitted ellipse), hit ‘build and run’ to verify the position of the spokes on the image ‘initialpoints’ saved in the folder.

Troubleshooting. Sometimes C++ runs the last code used (strain instead of strain_spokes for example). In this case, click right on the project “LungSlice” and “clean”. Add the *main_strain_calc_spokes.cpp* again

*Good to know:
-you can play a bit with the ‘orientation’ value to orient the spokes in a particular directions (to make one go through a blood vessel for example)
-smaller numbers bring the spoke towards the center of the lumen*

When the initial spokes are positioned, turn the setup on line 13 back to 0 and hit ‘build and run’.

BE CAREFUL to use the same parameters as for the strain maps.

Strain data computed with the spoke method are saved in the folder.

3/ In Matlab, open **F_post_strain_calc_spokes.m**, specify the parameters in the code: the folder and the number of the frames analyzed (copy/paste from ellipse.txt)

Run the code, profiles of averaged strains along the spokes are saved in the folder (.tif).

4/ Open **G_post_strainVSdistance_spokes.m**, specify the parameters in the code: the folder and the number of the frames analyzed (copy/paste from ellipse.txt)

Run the code, kymographs of major, minor and average strains are plotted for all the spokes (.tif).

In case of image drift during image acquisition of the experiment

Use the code *imageshift.m* (in the Strain_Map folder) to correct for the drift on the relevant part of the image sequence prior to running the strain map analysis.

*Tip*: if the list of images to correct is long, you can run the code A_pre_strain, to obtain the ellipse.txt file and copy paste the list of frame numbers from there.

Instructions to run *imageshift.m* are in the code.


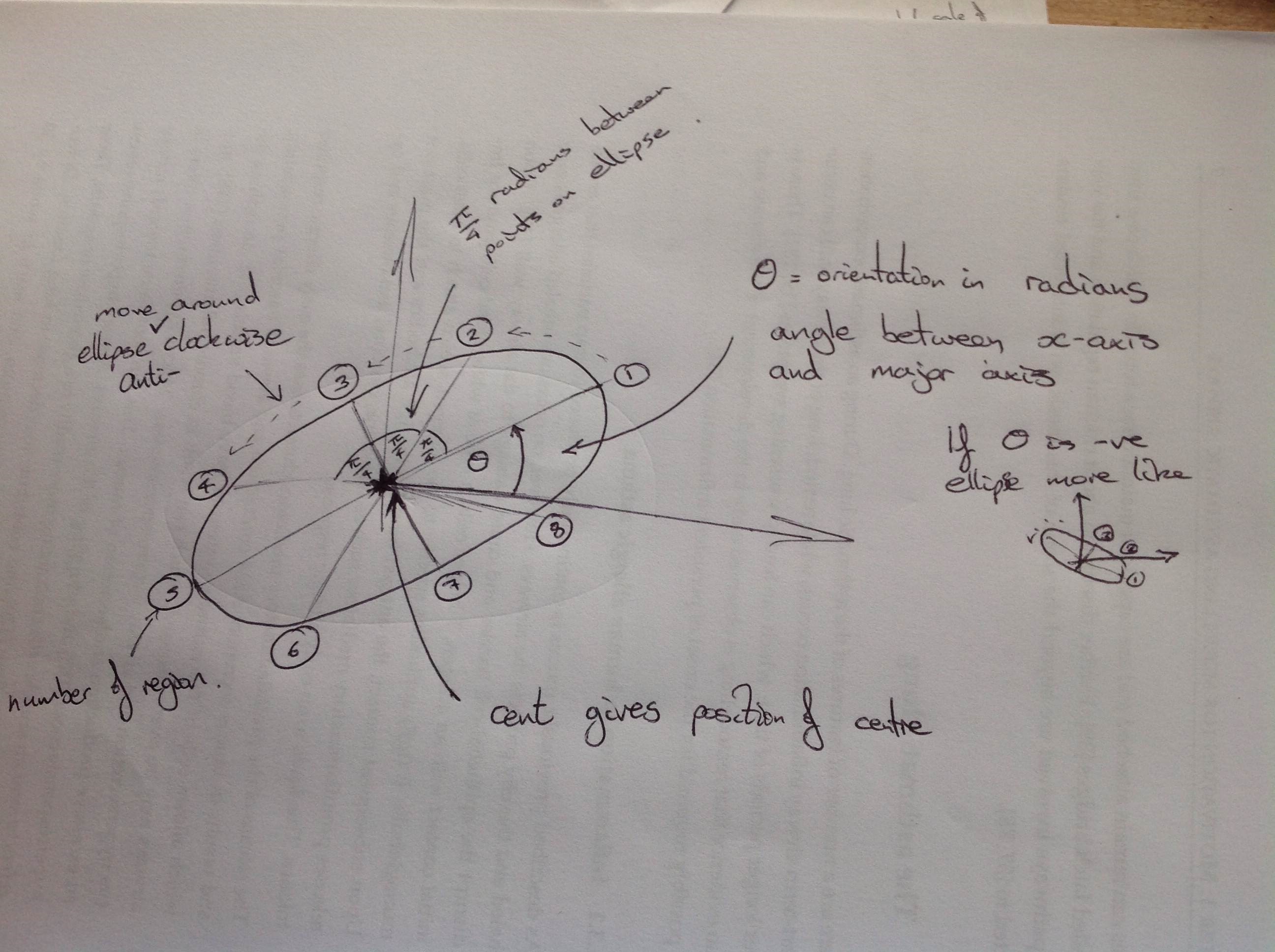

Supplement: Supplementary file 4 [file Presentation1.zip › StrainMap_package_21-08/Strain_Map/How_to_use_the_codes.docx]
