# Supplementary figures and images for: Airway and Parenchymal Strains during Bronchoconstriction in the Precision Cut Lung Slice
Source: Front Physiol. 2016 Jul 21;7:309. doi: 10.3389/fphys.2016.00309 (PMC4989902; doi:10.3389/fphys.2016.00309)

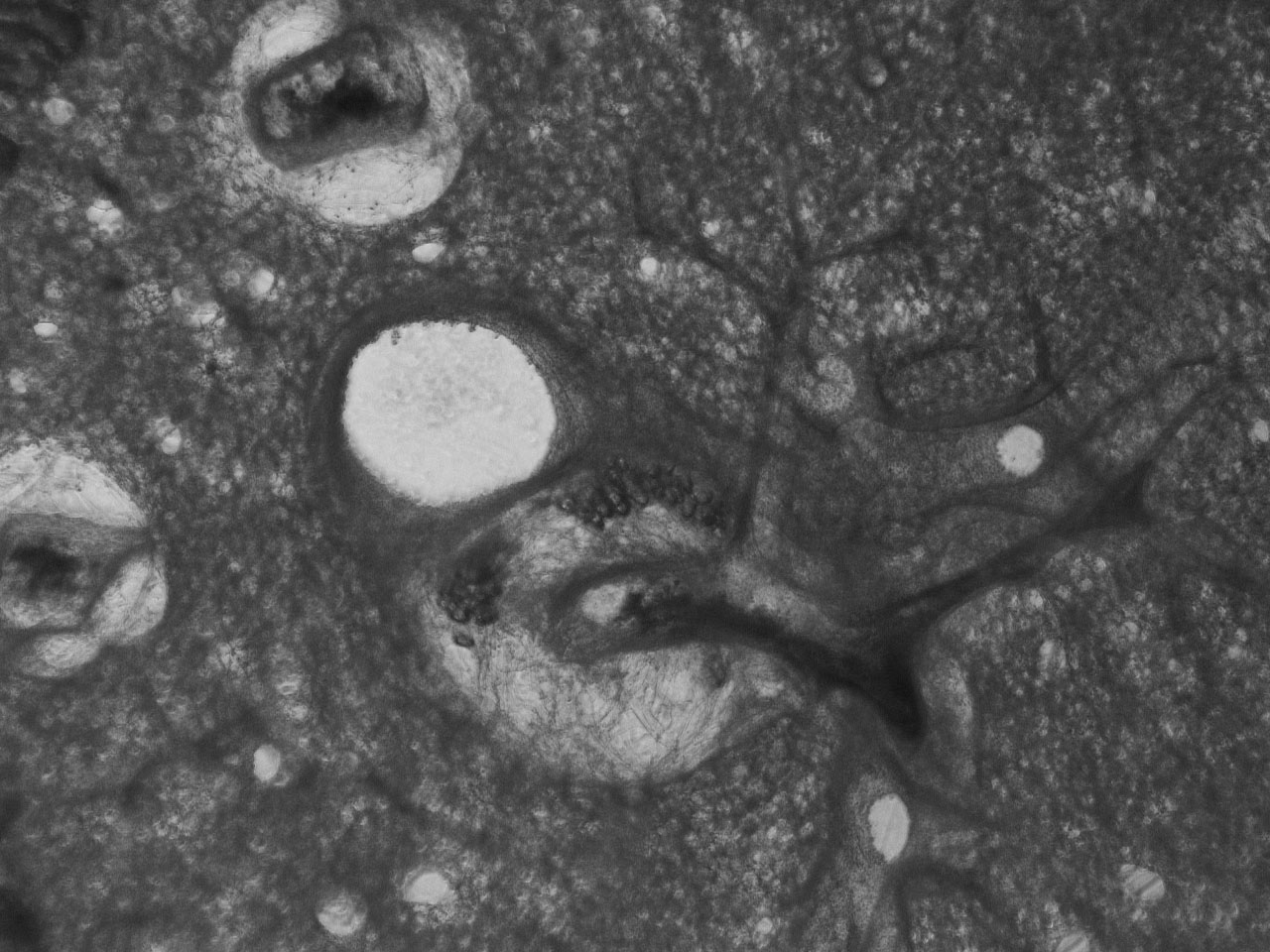

Supplement: Supplementary file 4 [file Presentation1.zip › StrainMap_package_21-08/Strain_Map/A1_c1_01_clean/frame0055.jpg]

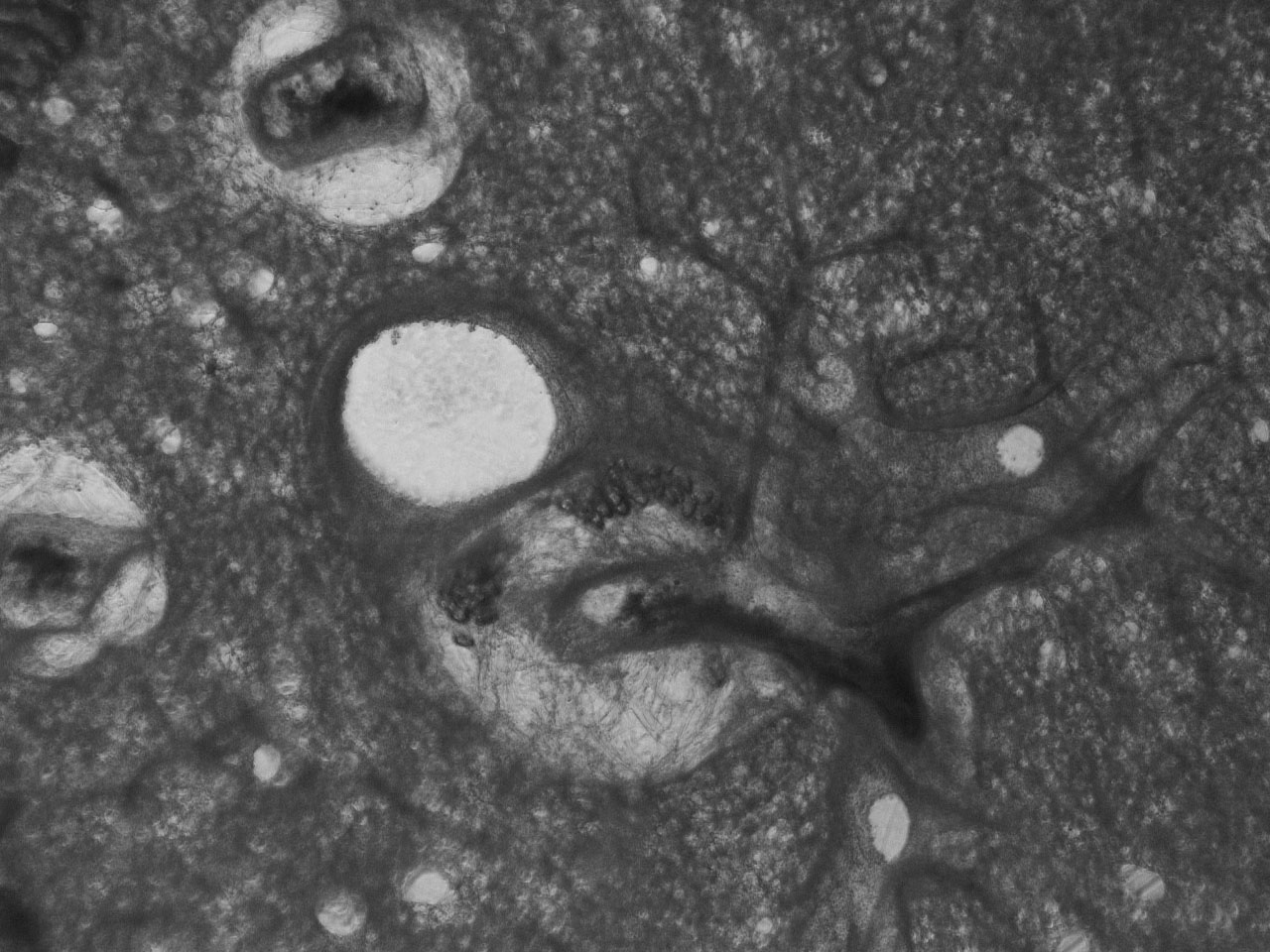

Supplement: Supplementary file 4 [file Presentation1.zip › StrainMap_package_21-08/Strain_Map/A1_c1_01_clean/frame0065.jpg]

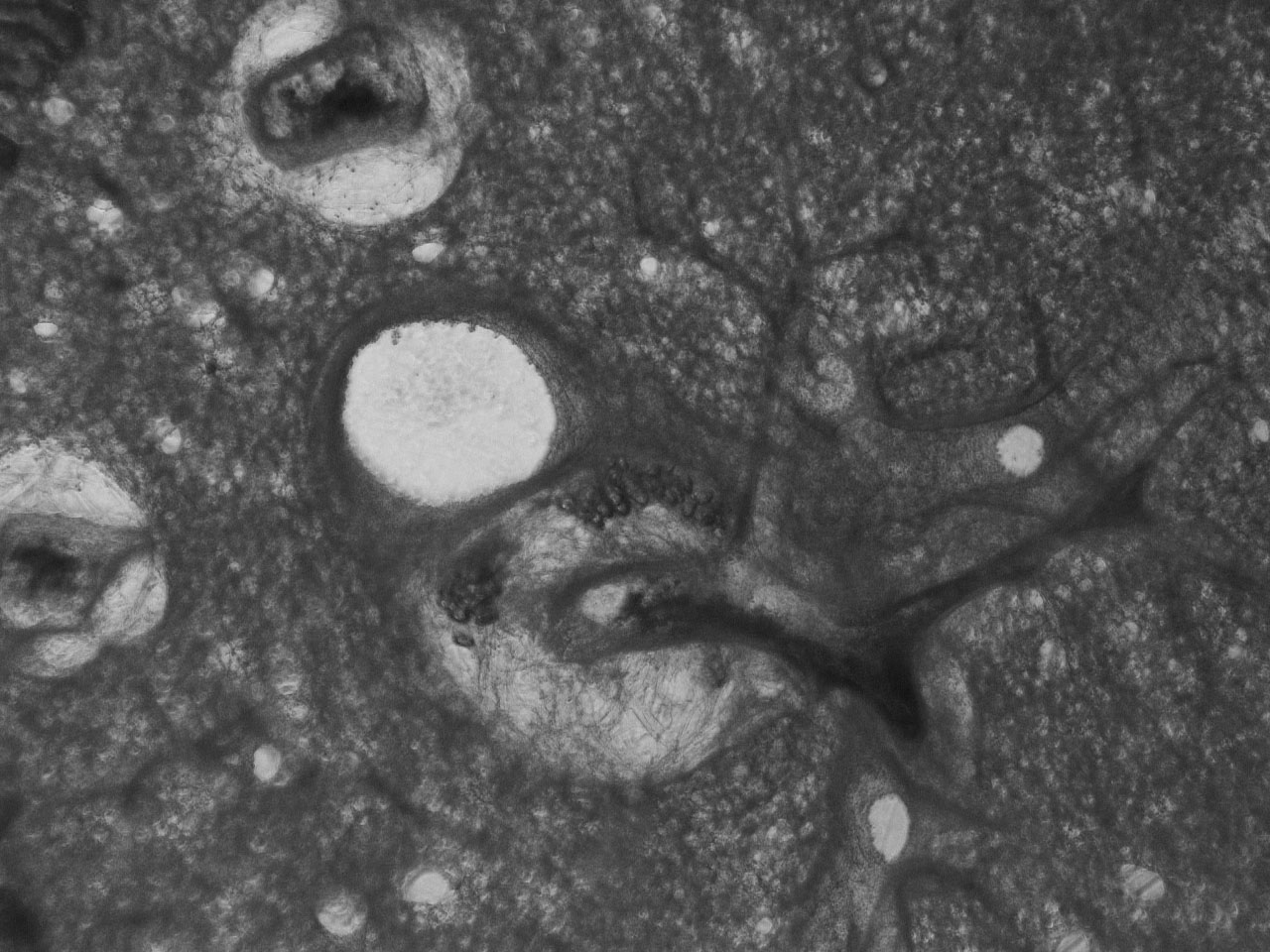

Supplement: Supplementary file 4 [file Presentation1.zip › StrainMap_package_21-08/Strain_Map/A1_c1_01_clean/frame0075.jpg]

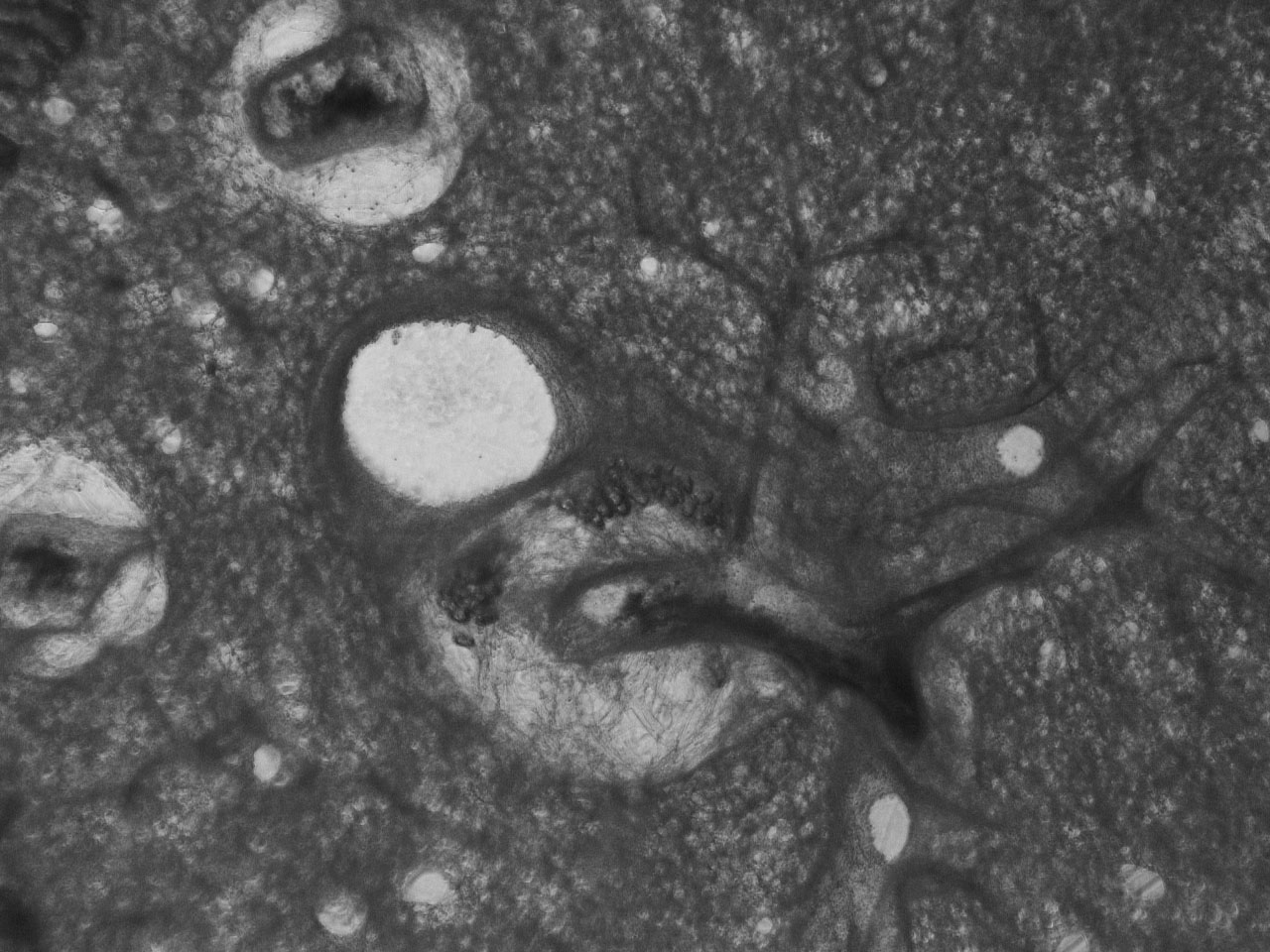

Supplement: Supplementary file 4 [file Presentation1.zip › StrainMap_package_21-08/Strain_Map/A1_c1_01_clean/frame0085.jpg]

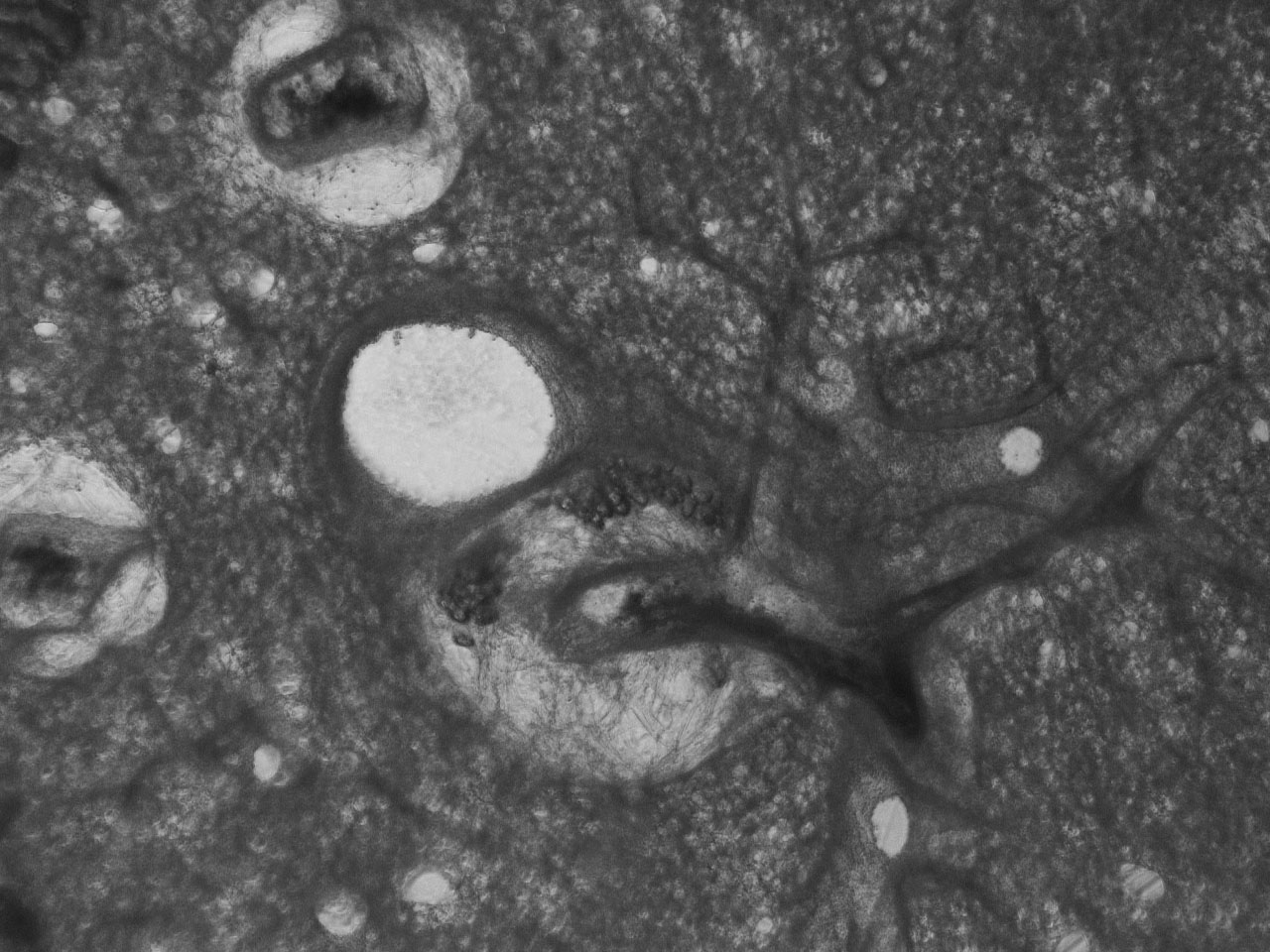

Supplement: Supplementary file 4 [file Presentation1.zip › StrainMap_package_21-08/Strain_Map/A1_c1_01_clean/frame0095.jpg]

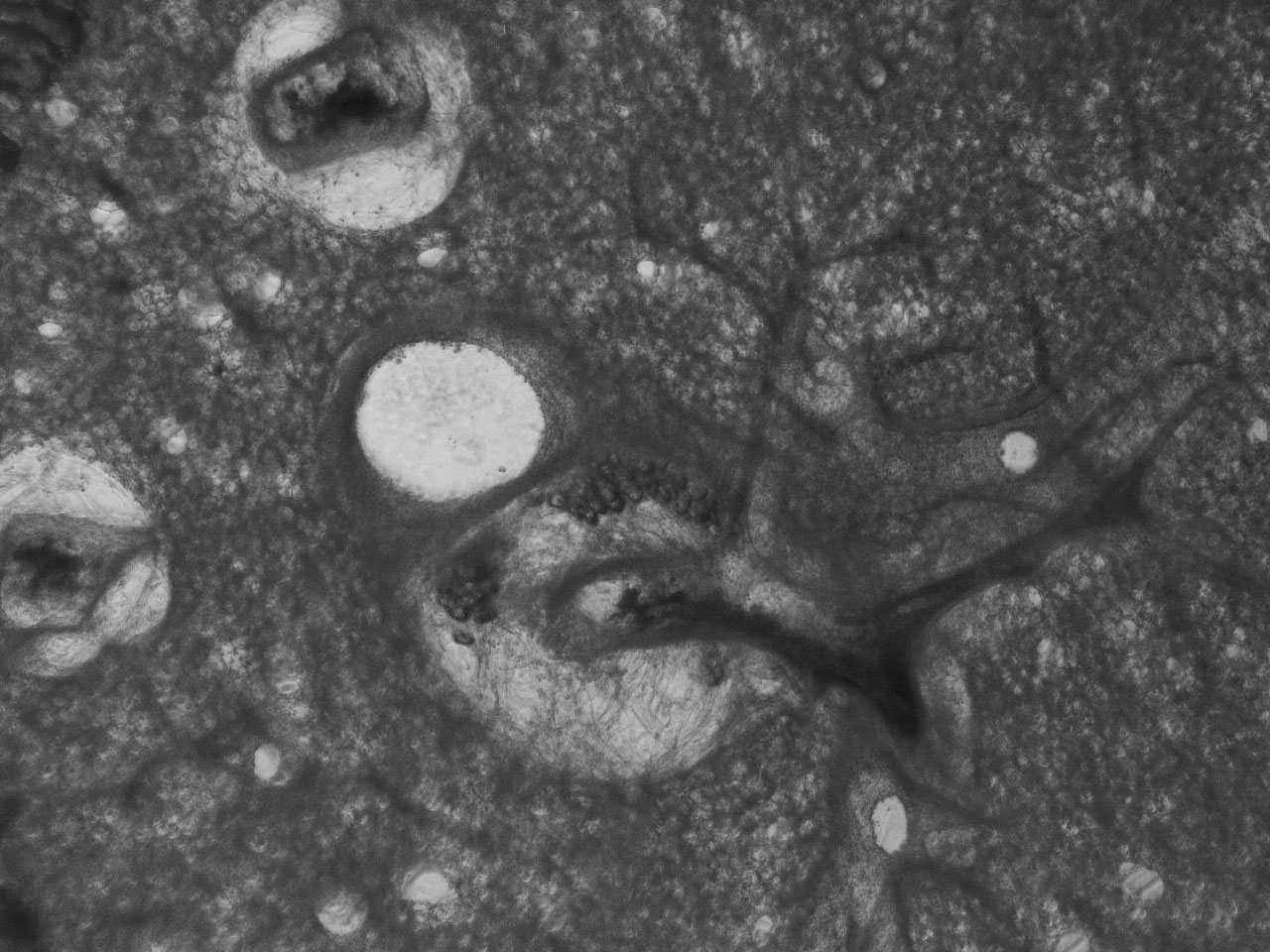

Supplement: Supplementary file 4 [file Presentation1.zip › StrainMap_package_21-08/Strain_Map/A1_c1_01_clean/frame0105.jpg]

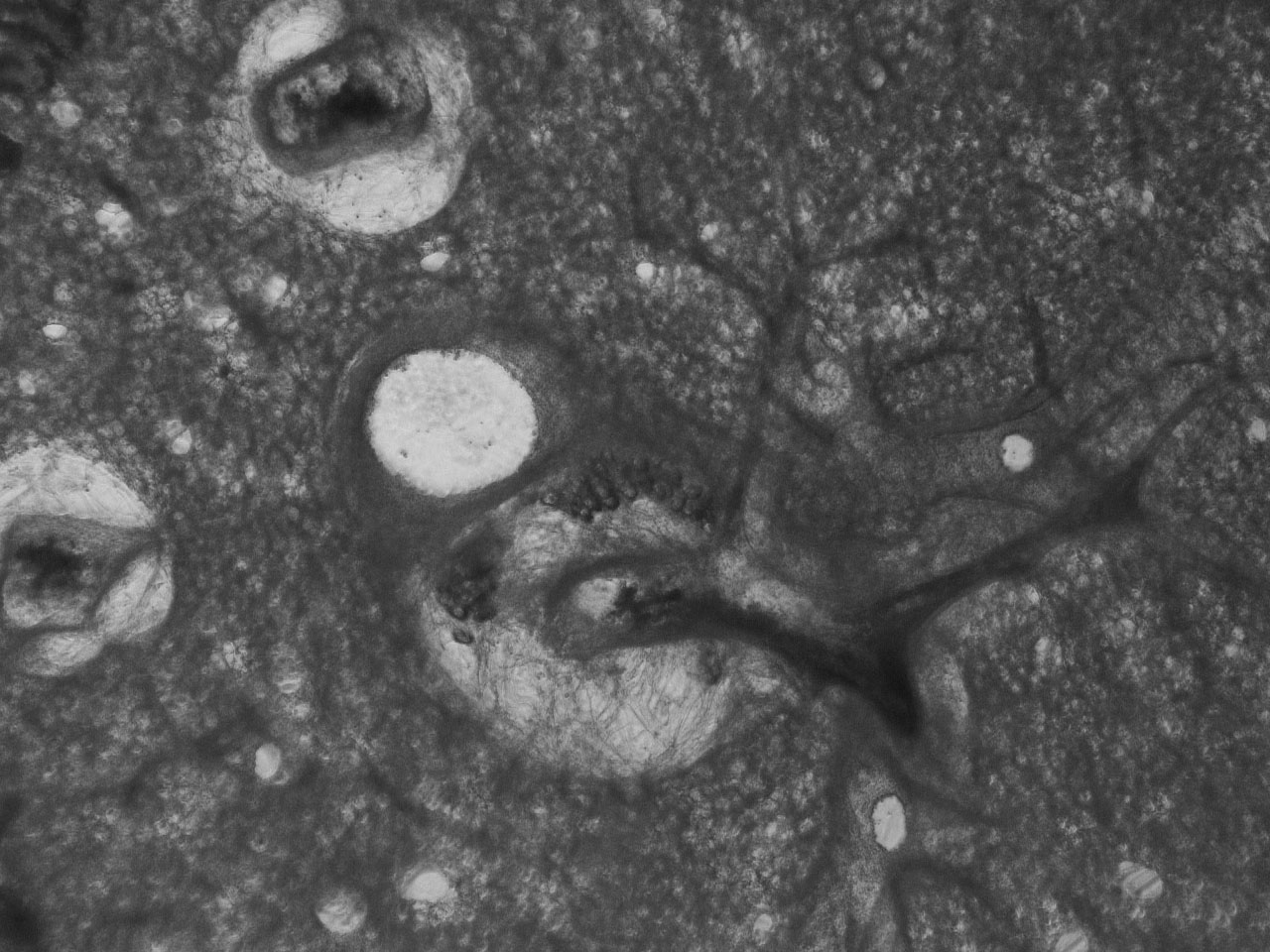

Supplement: Supplementary file 4 [file Presentation1.zip › StrainMap_package_21-08/Strain_Map/A1_c1_01_clean/frame0115.jpg]

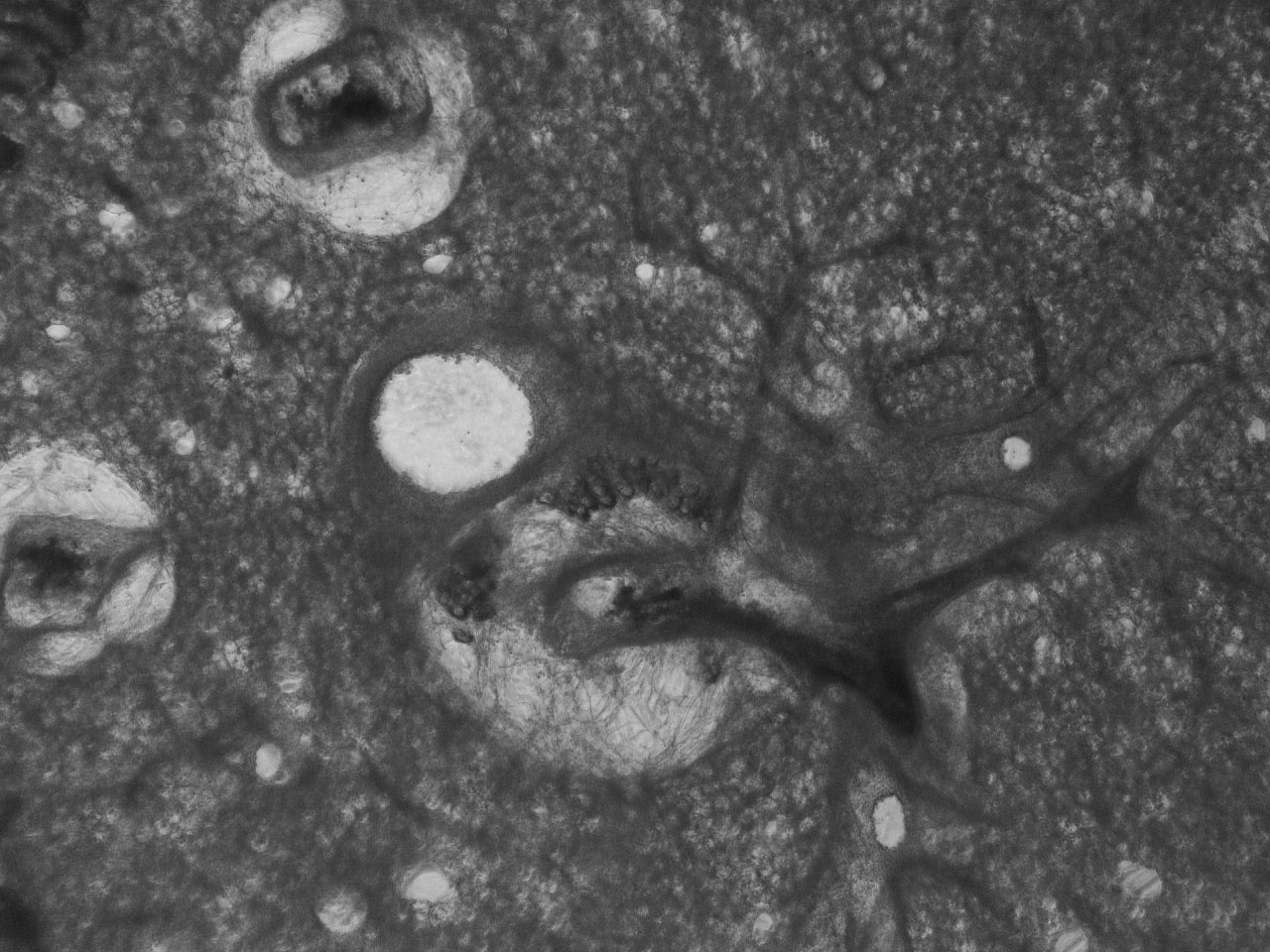

Supplement: Supplementary file 4 [file Presentation1.zip › StrainMap_package_21-08/Strain_Map/A1_c1_01_clean/frame0125.jpg]

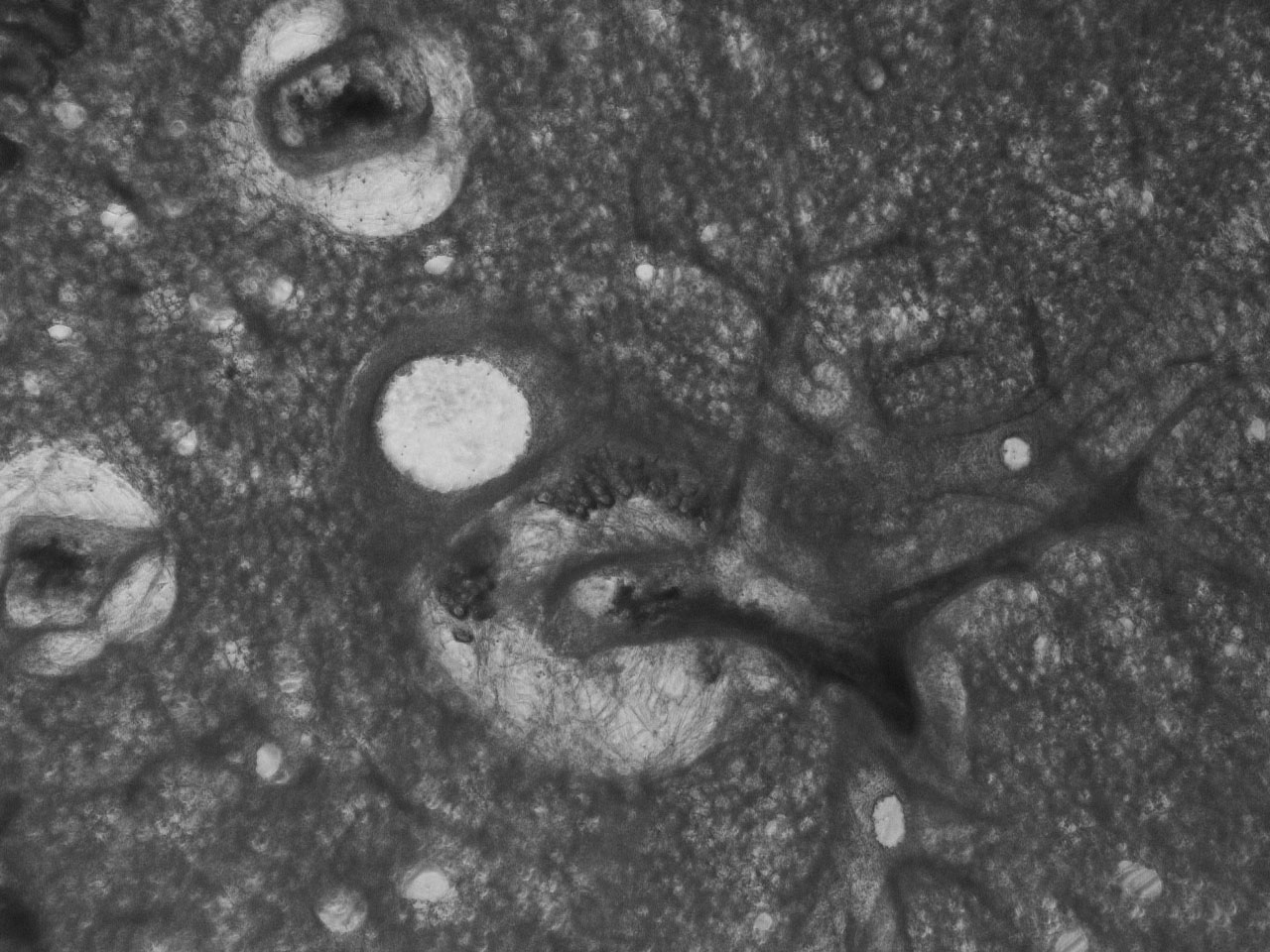

Supplement: Supplementary file 4 [file Presentation1.zip › StrainMap_package_21-08/Strain_Map/A1_c1_01_clean/frame0135.jpg]

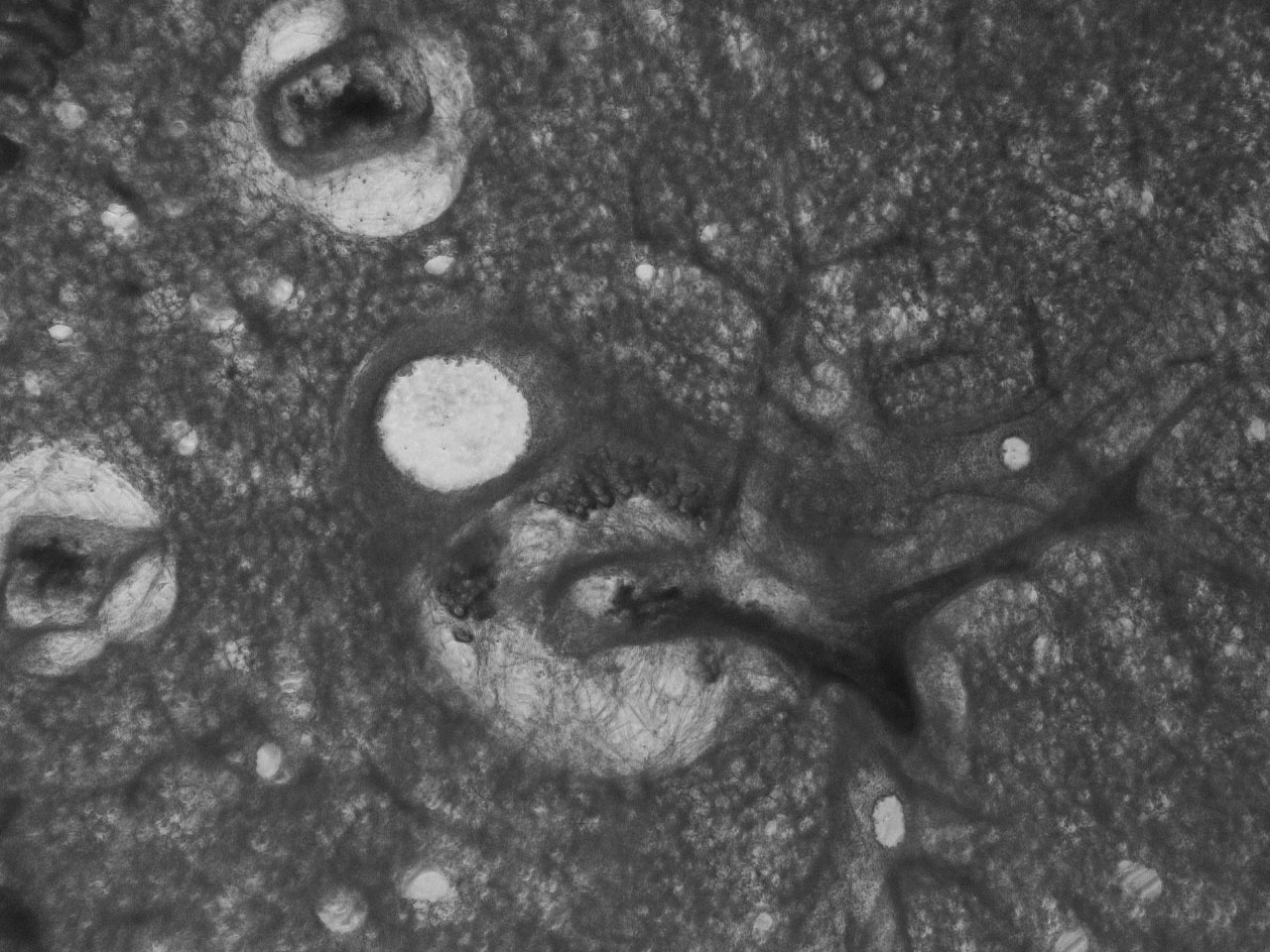

Supplement: Supplementary file 4 [file Presentation1.zip › StrainMap_package_21-08/Strain_Map/A1_c1_01_clean/frame0145.jpg]

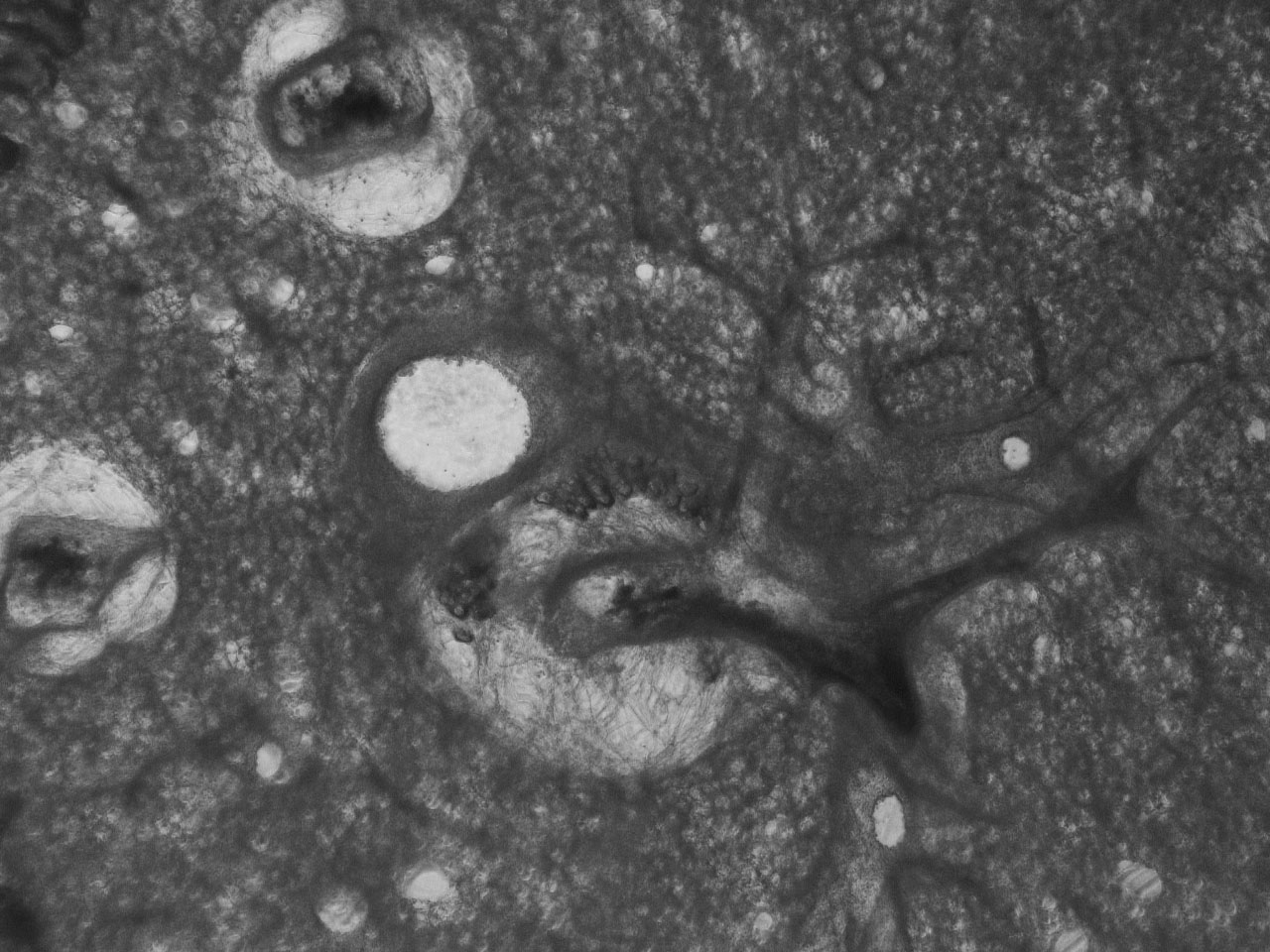

Supplement: Supplementary file 4 [file Presentation1.zip › StrainMap_package_21-08/Strain_Map/A1_c1_01_clean/frame0155.jpg]

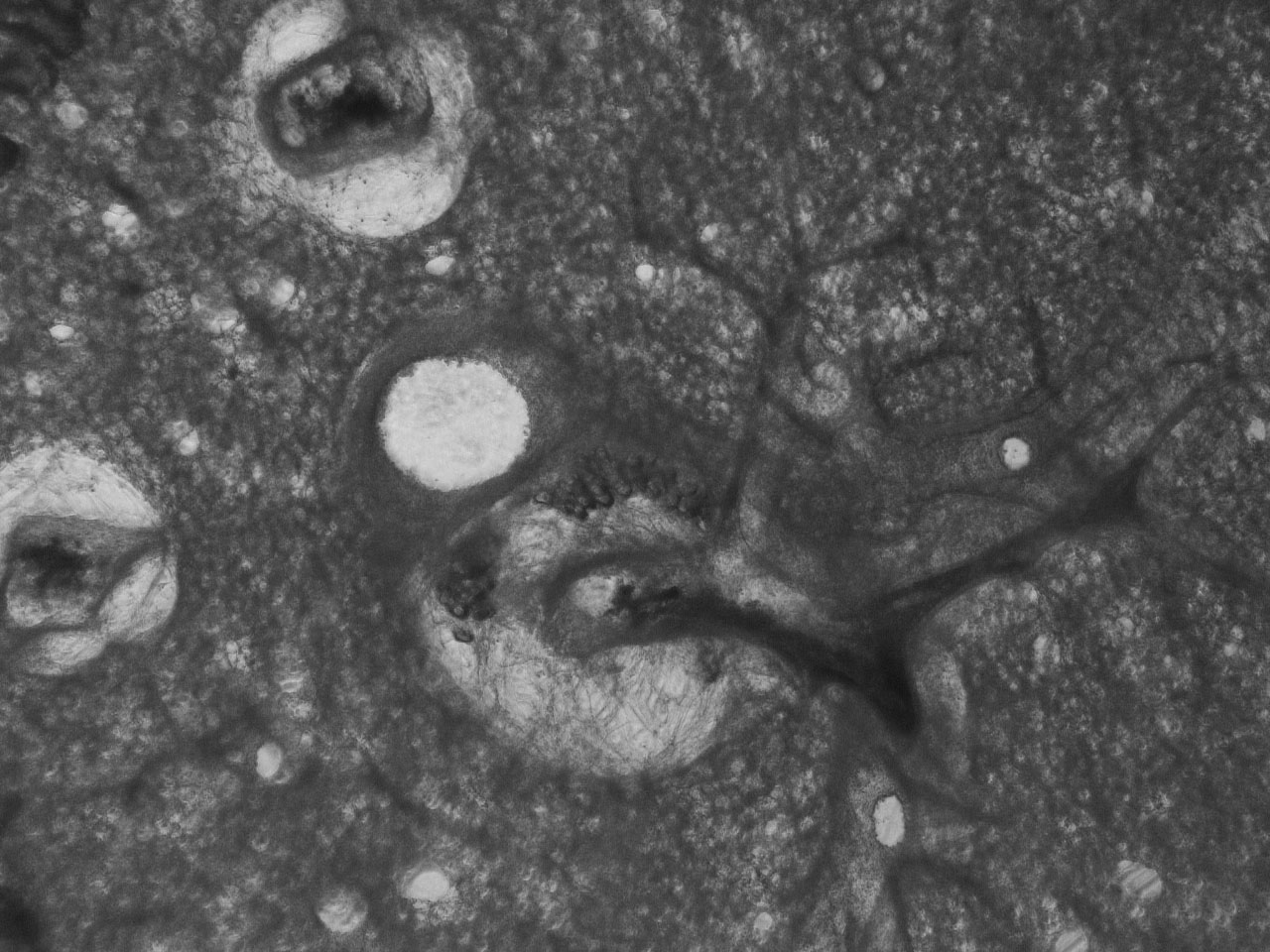

Supplement: Supplementary file 4 [file Presentation1.zip › StrainMap_package_21-08/Strain_Map/A1_c1_01_clean/frame0165.jpg]

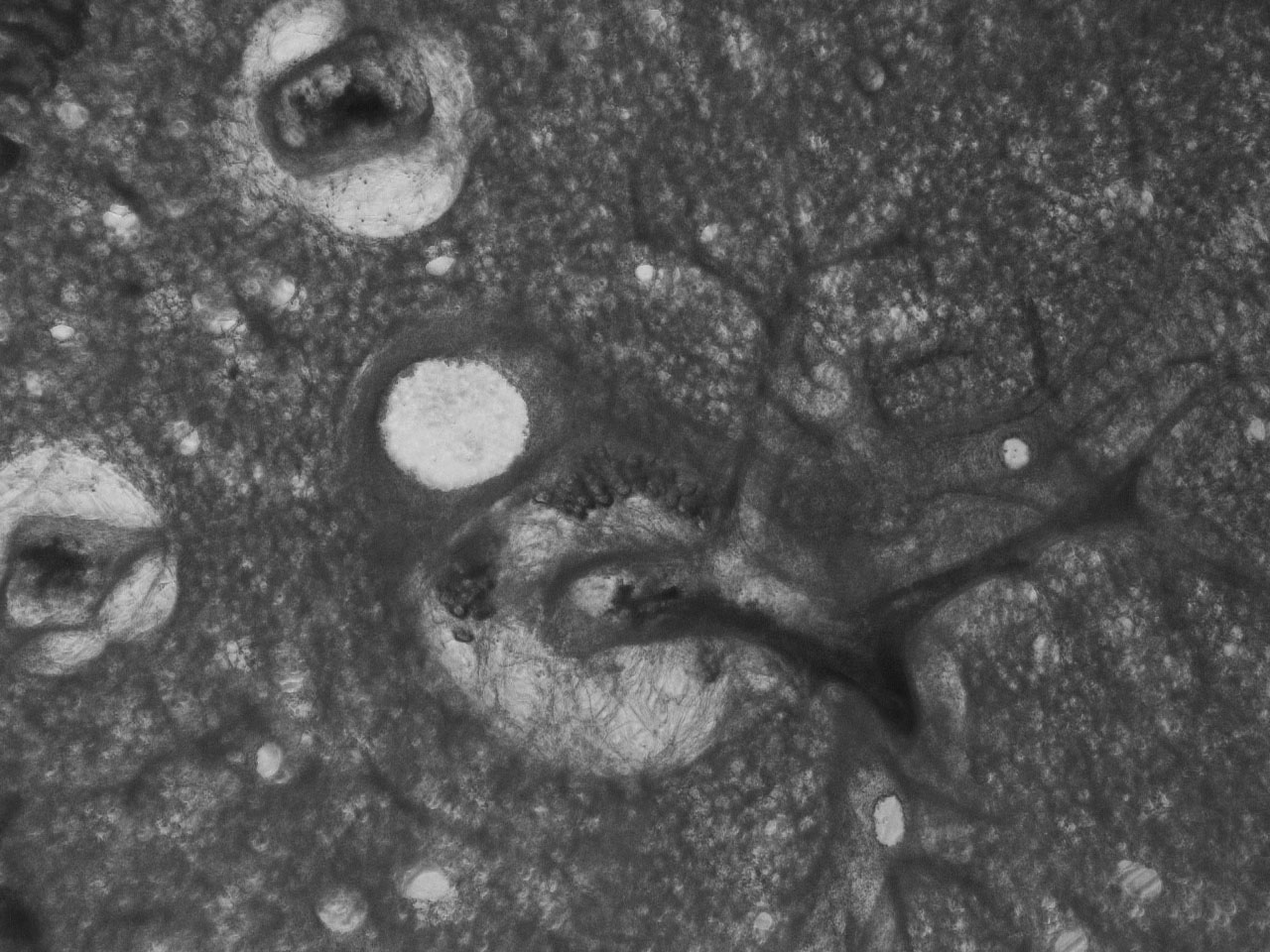

Supplement: Supplementary file 4 [file Presentation1.zip › StrainMap_package_21-08/Strain_Map/A1_c1_01_clean/frame0175.jpg]

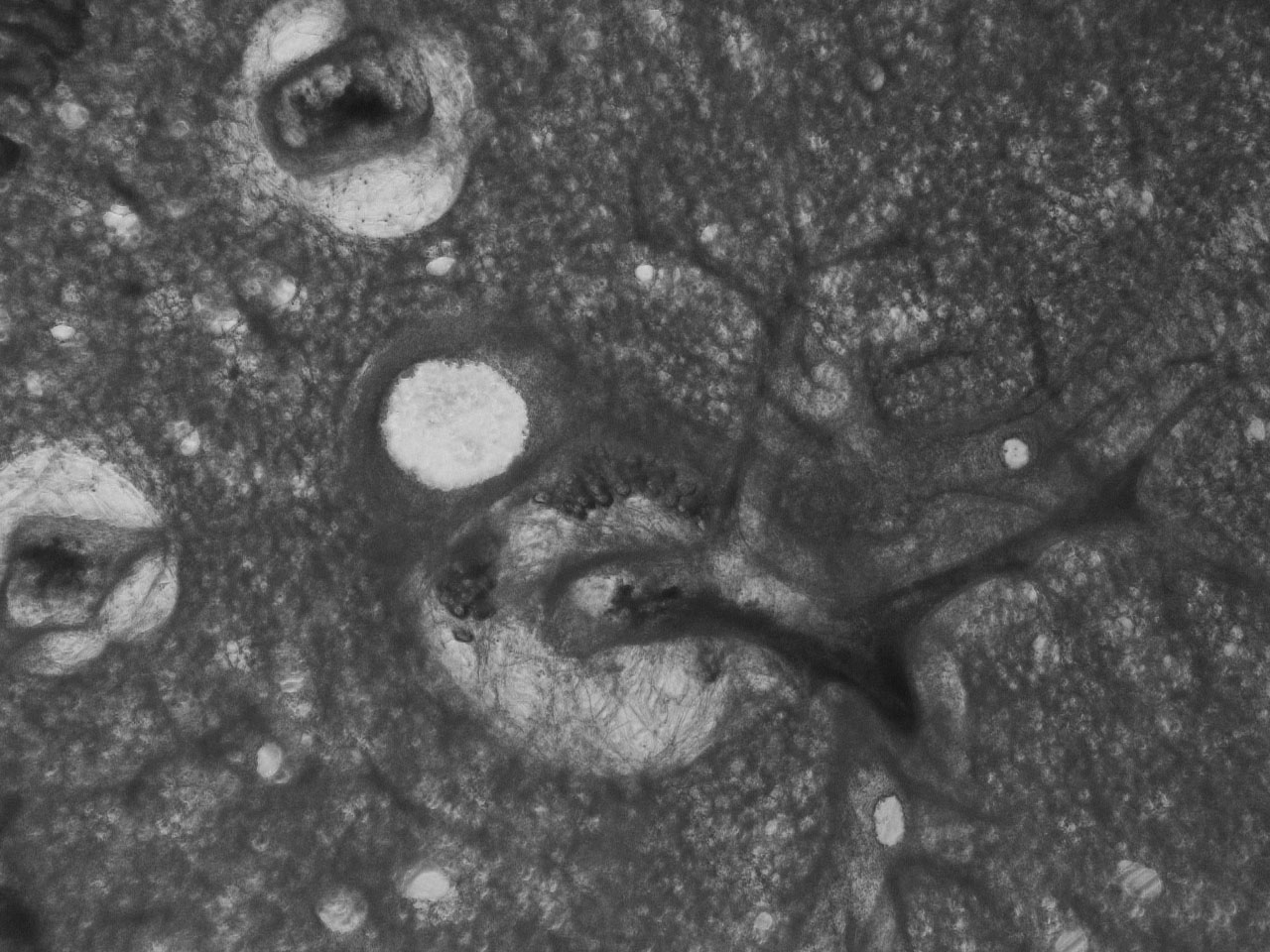

Supplement: Supplementary file 4 [file Presentation1.zip › StrainMap_package_21-08/Strain_Map/A1_c1_01_clean/frame0185.jpg]

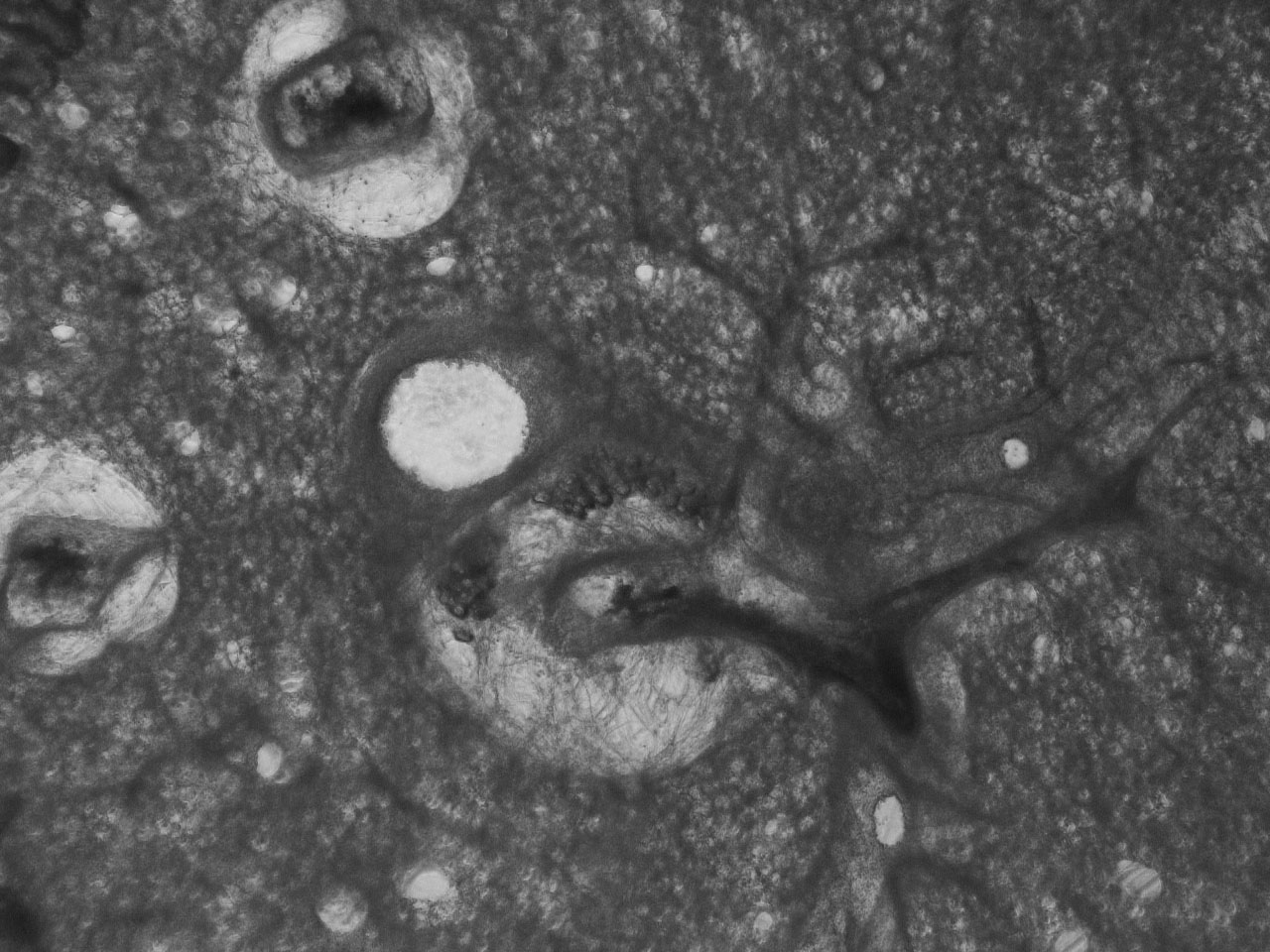

Supplement: Supplementary file 4 [file Presentation1.zip › StrainMap_package_21-08/Strain_Map/A1_c1_01_clean/frame0195.jpg]

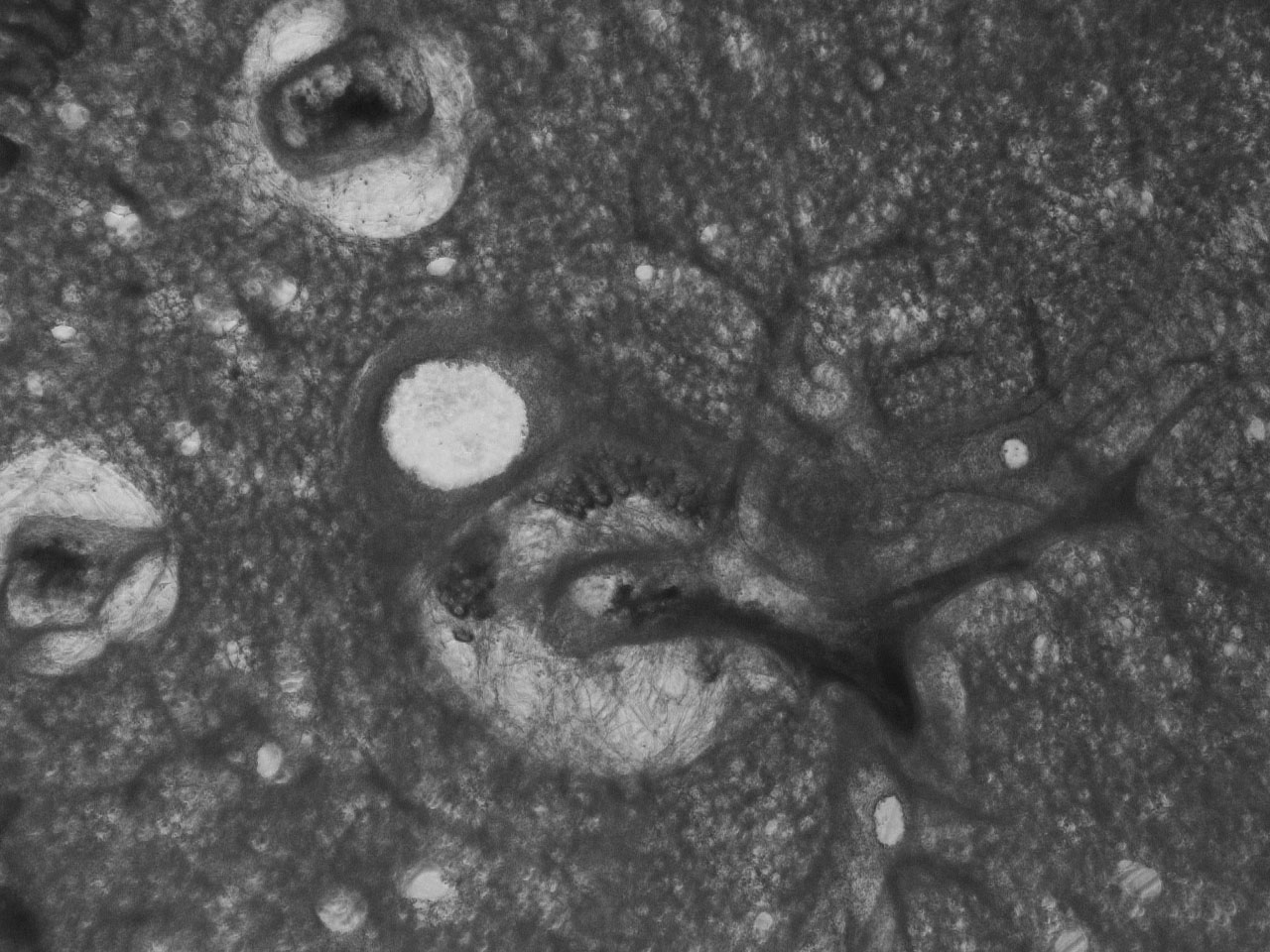

Supplement: Supplementary file 4 [file Presentation1.zip › StrainMap_package_21-08/Strain_Map/A1_c1_01_clean/frame0205.jpg]

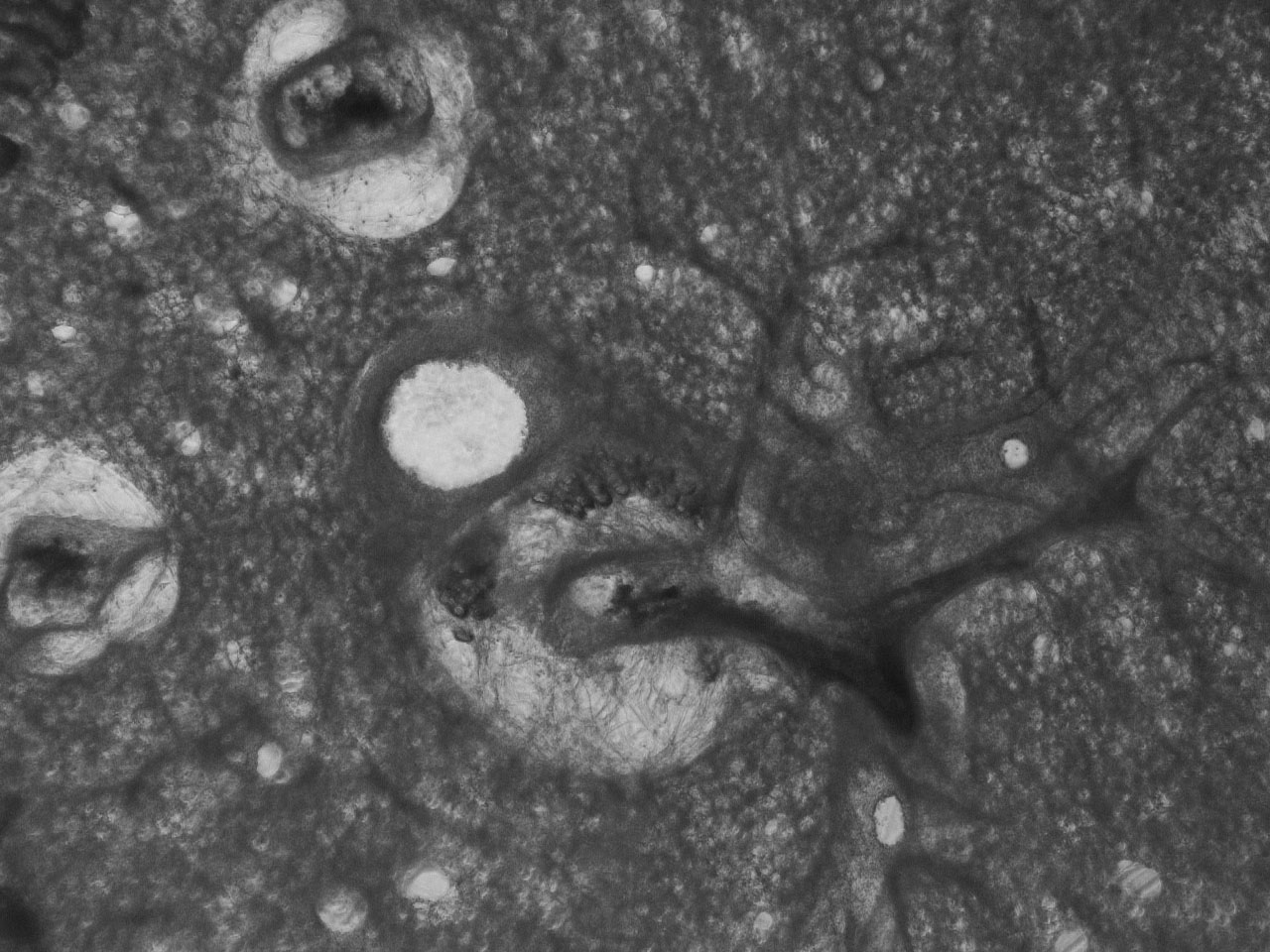

Supplement: Supplementary file 4 [file Presentation1.zip › StrainMap_package_21-08/Strain_Map/A1_c1_01_clean/frame0215.jpg]

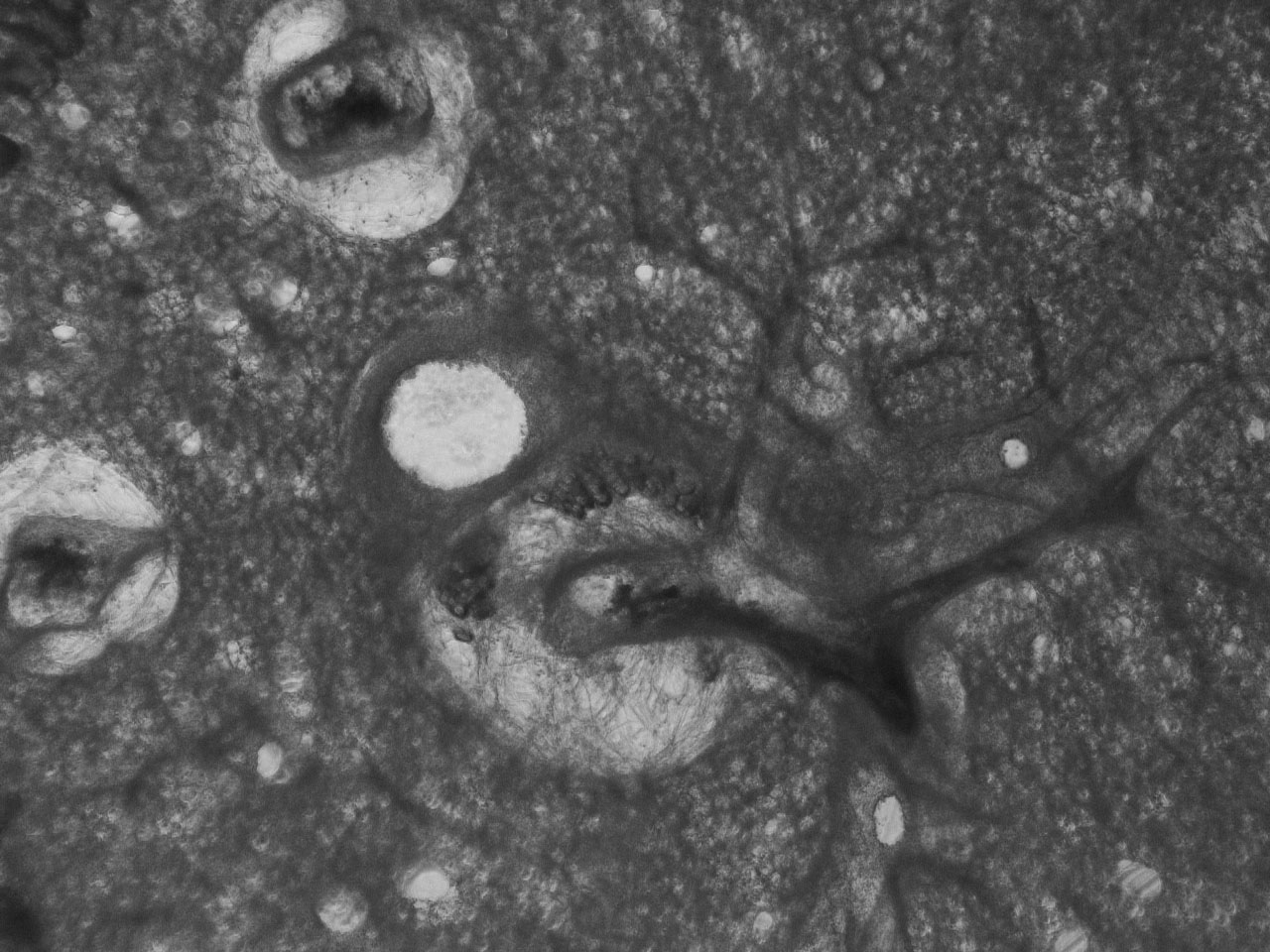

Supplement: Supplementary file 4 [file Presentation1.zip › StrainMap_package_21-08/Strain_Map/A1_c1_01_clean/frame0225.jpg]

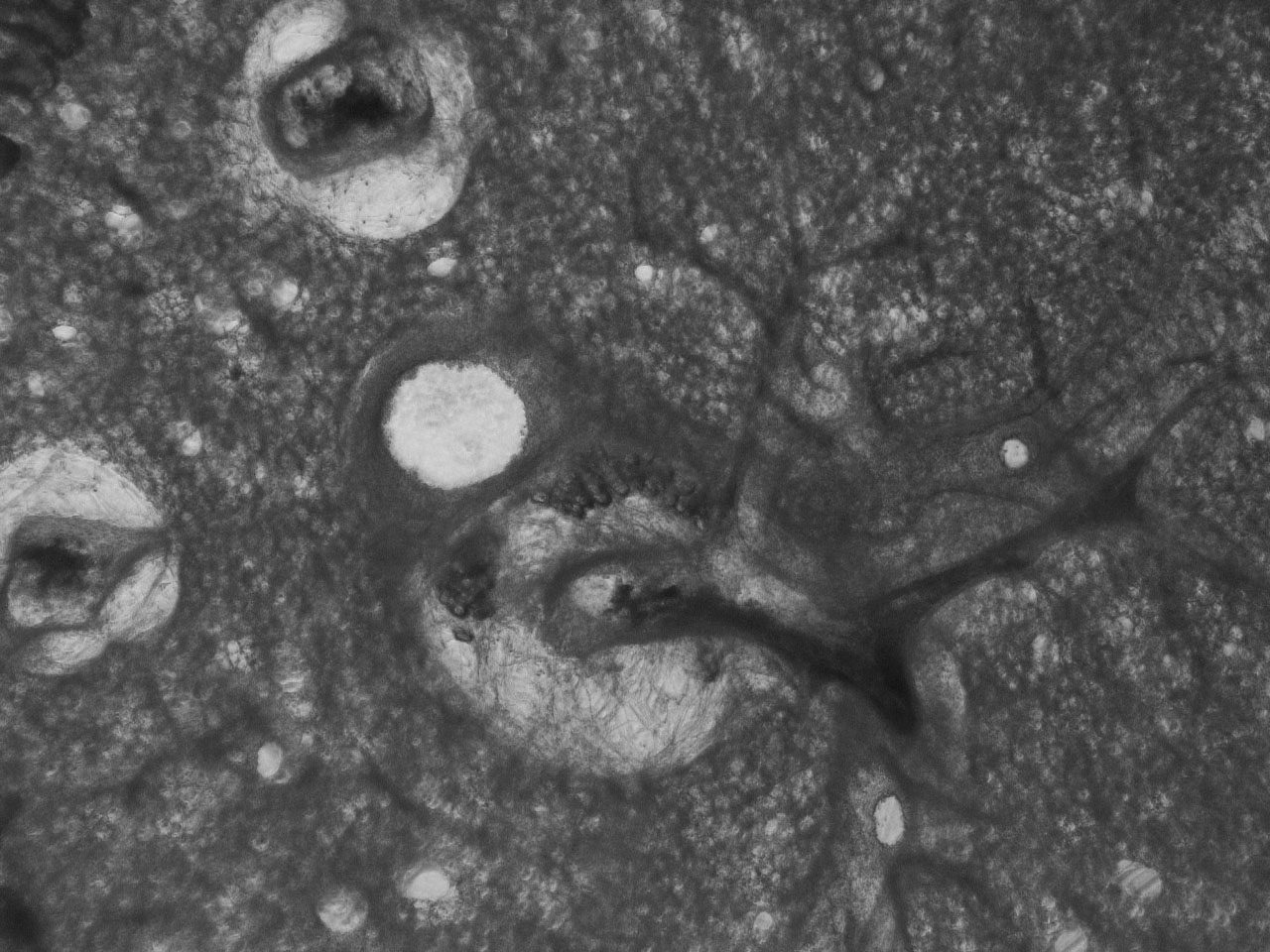

Supplement: Supplementary file 4 [file Presentation1.zip › StrainMap_package_21-08/Strain_Map/A1_c1_01_clean/frame0235.jpg]

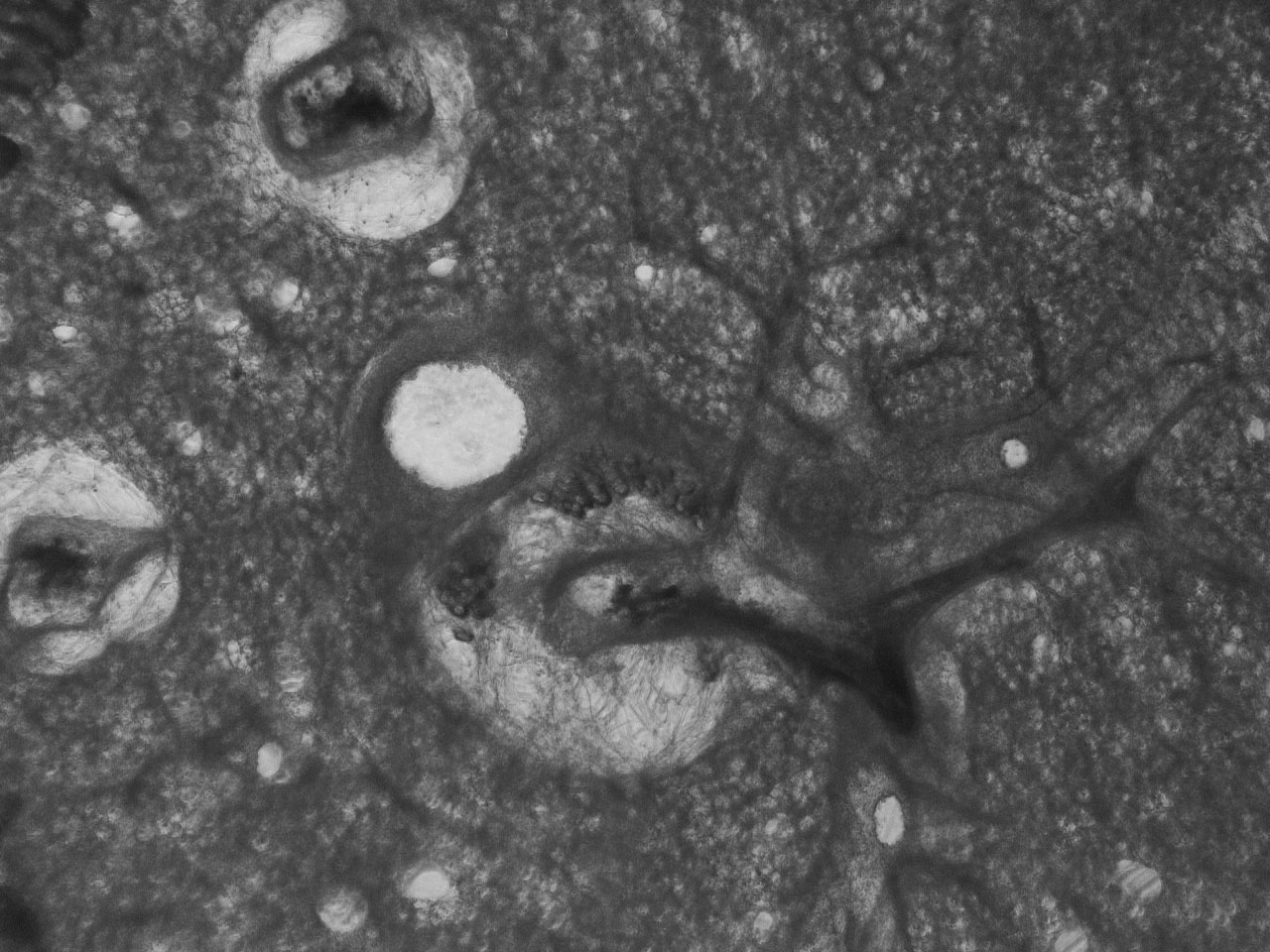

Supplement: Supplementary file 4 [file Presentation1.zip › StrainMap_package_21-08/Strain_Map/A1_c1_01_clean/frame0245.jpg]

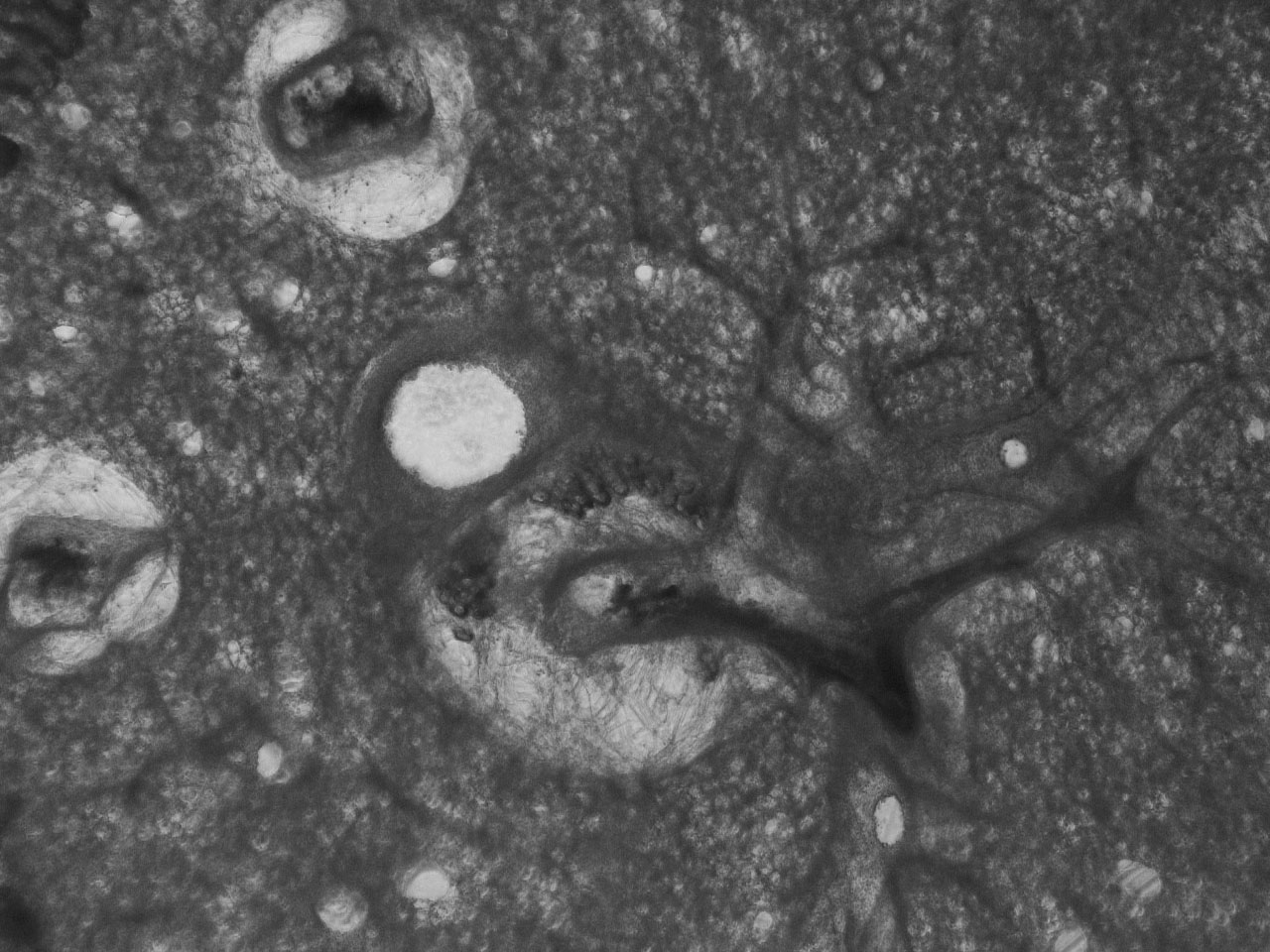

Supplement: Supplementary file 4 [file Presentation1.zip › StrainMap_package_21-08/Strain_Map/A1_c1_01_clean/frame0255.jpg]

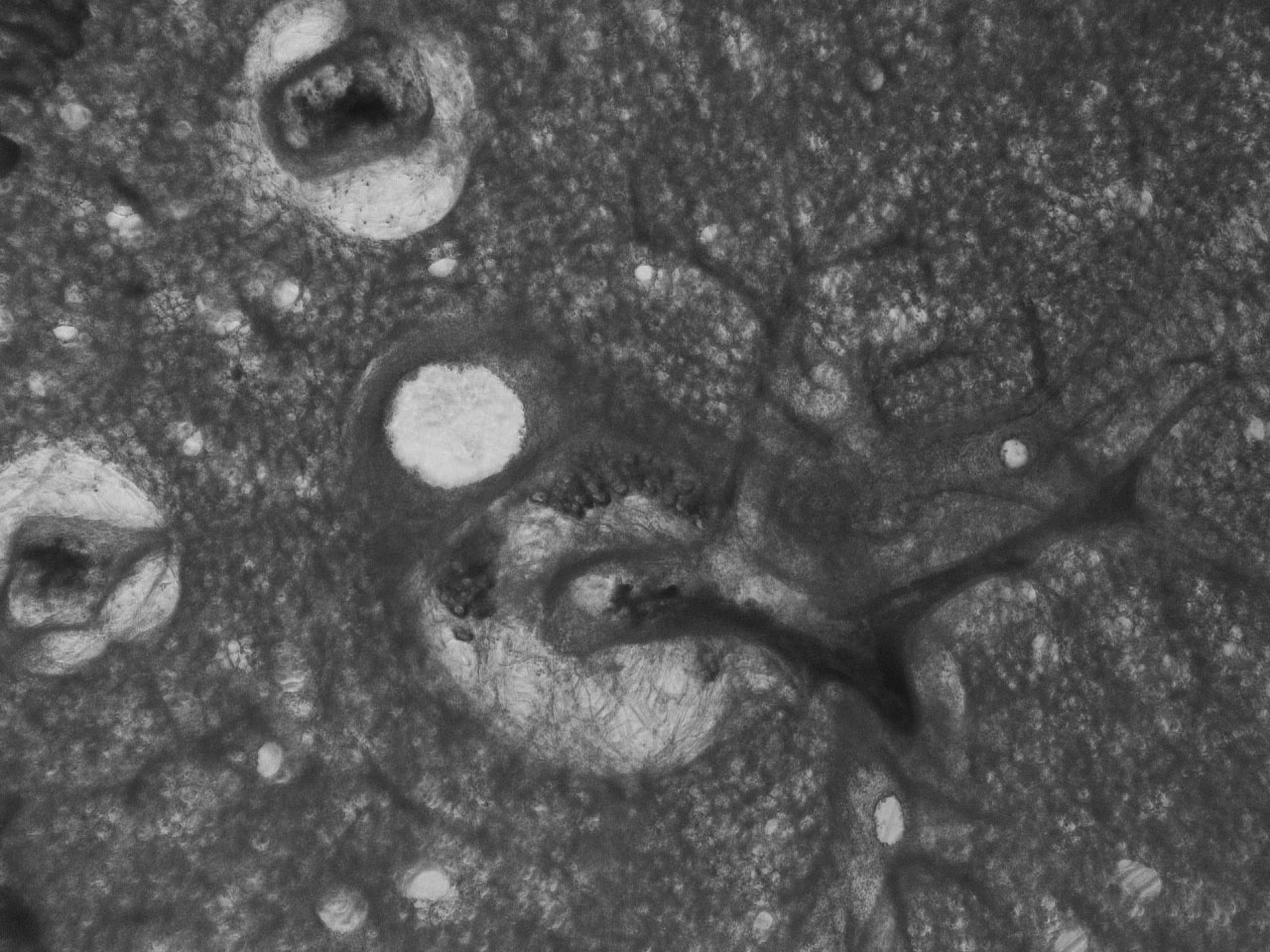

Supplement: Supplementary file 4 [file Presentation1.zip › StrainMap_package_21-08/Strain_Map/A1_c1_01_clean/frame0265.jpg]

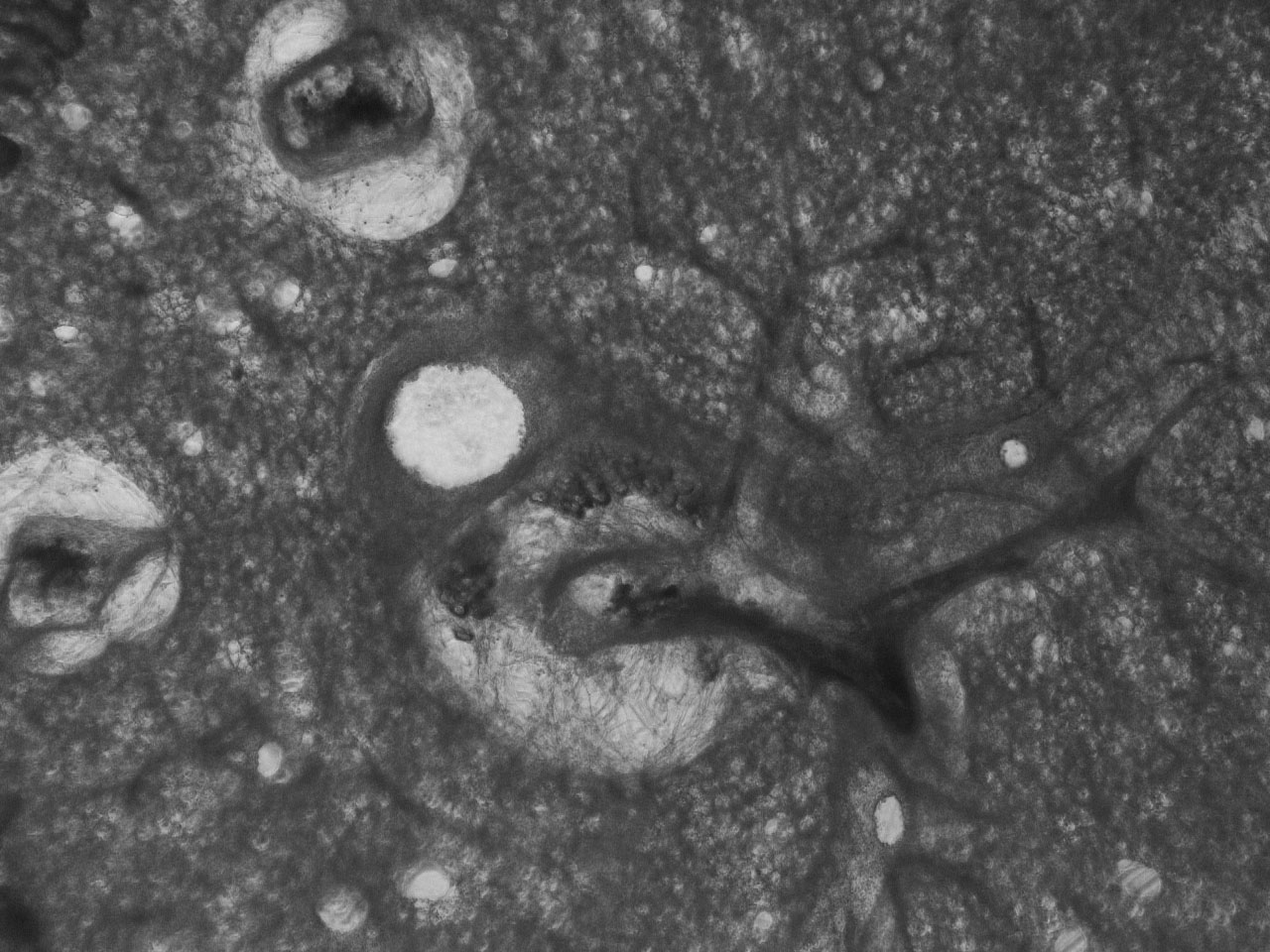

Supplement: Supplementary file 4 [file Presentation1.zip › StrainMap_package_21-08/Strain_Map/A1_c1_01_clean/frame0275.jpg]

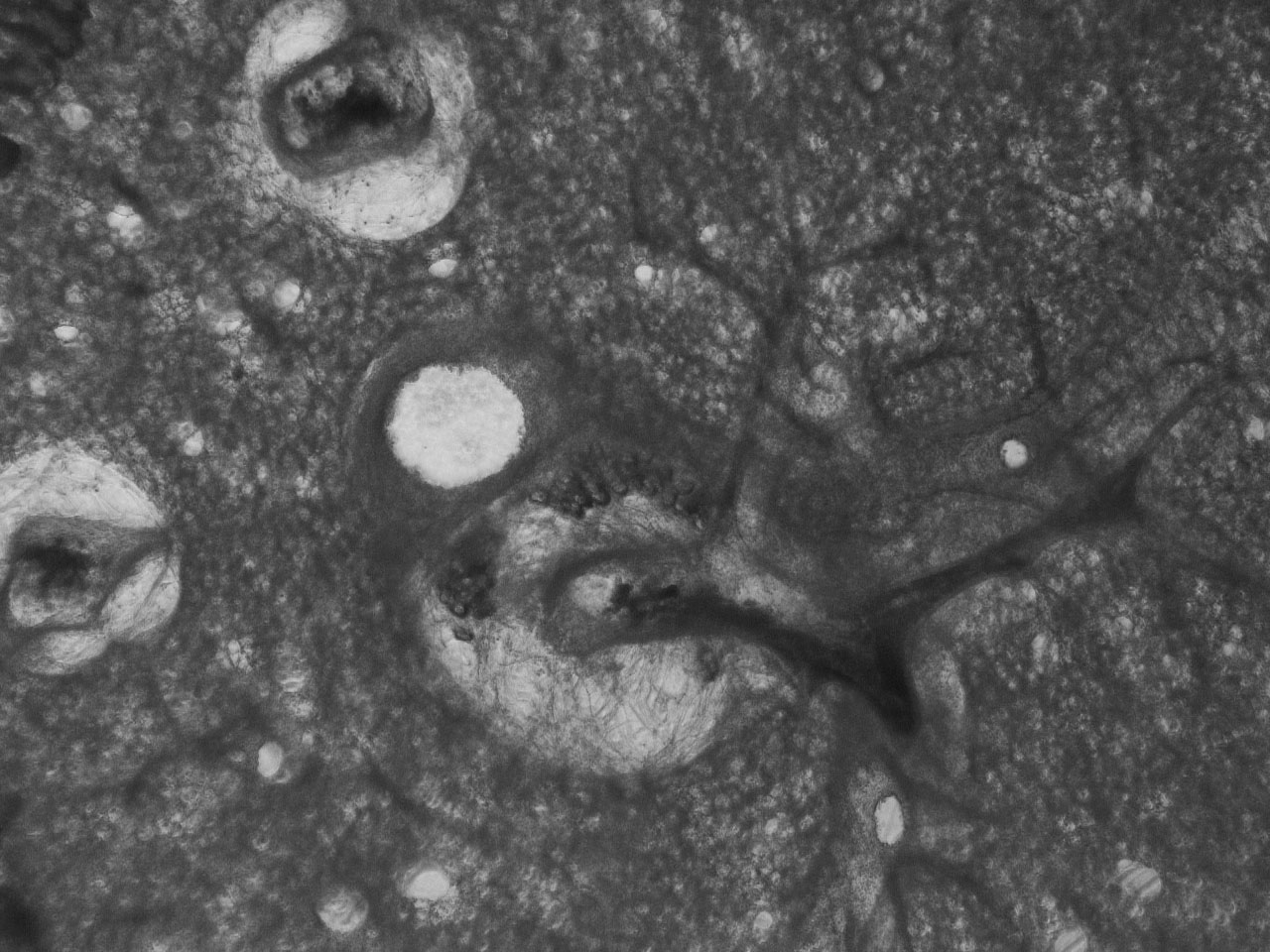

Supplement: Supplementary file 4 [file Presentation1.zip › StrainMap_package_21-08/Strain_Map/A1_c1_01_clean/frame0285.jpg]

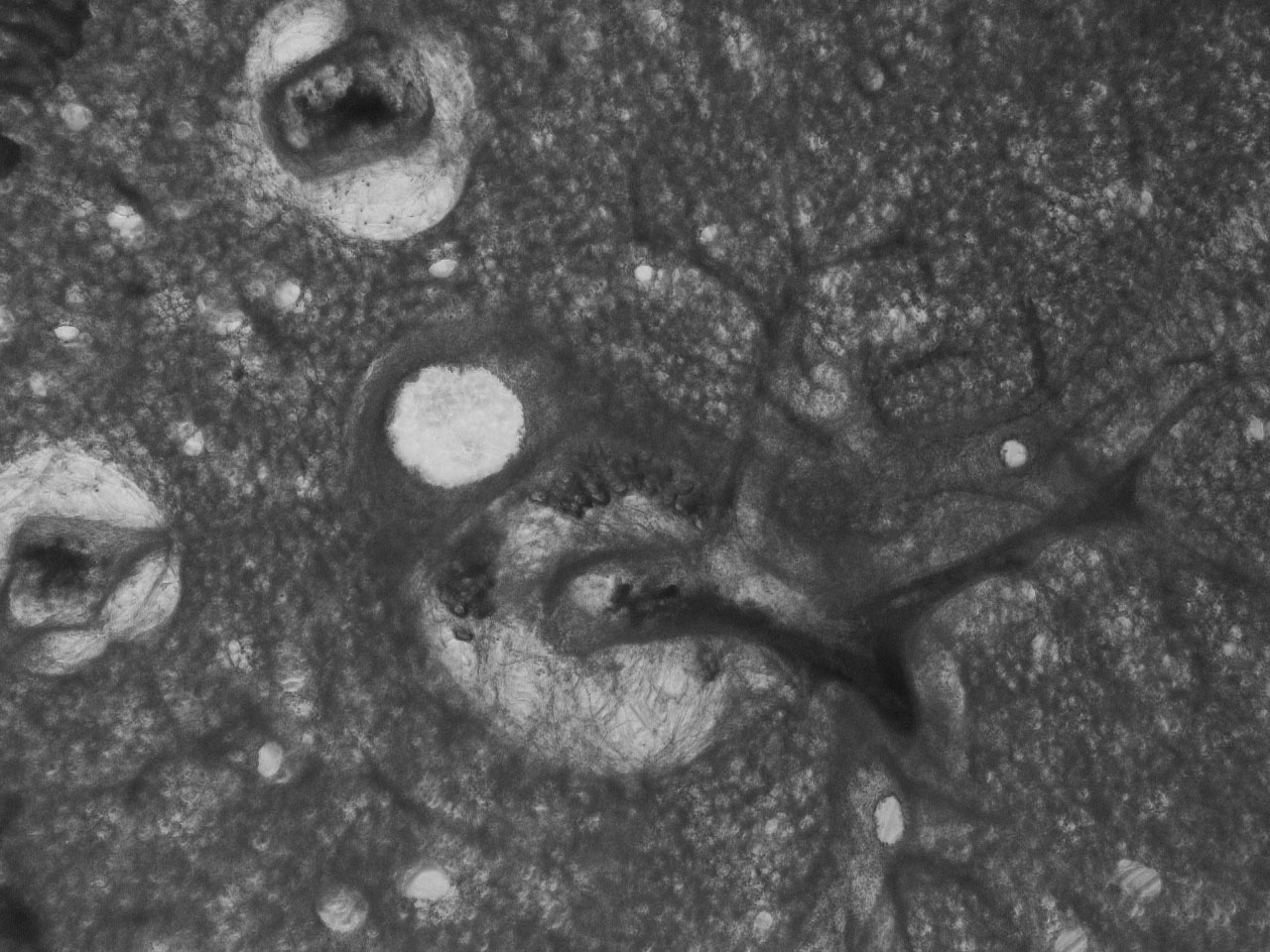

Supplement: Supplementary file 4 [file Presentation1.zip › StrainMap_package_21-08/Strain_Map/A1_c1_01_clean/frame0295.jpg]

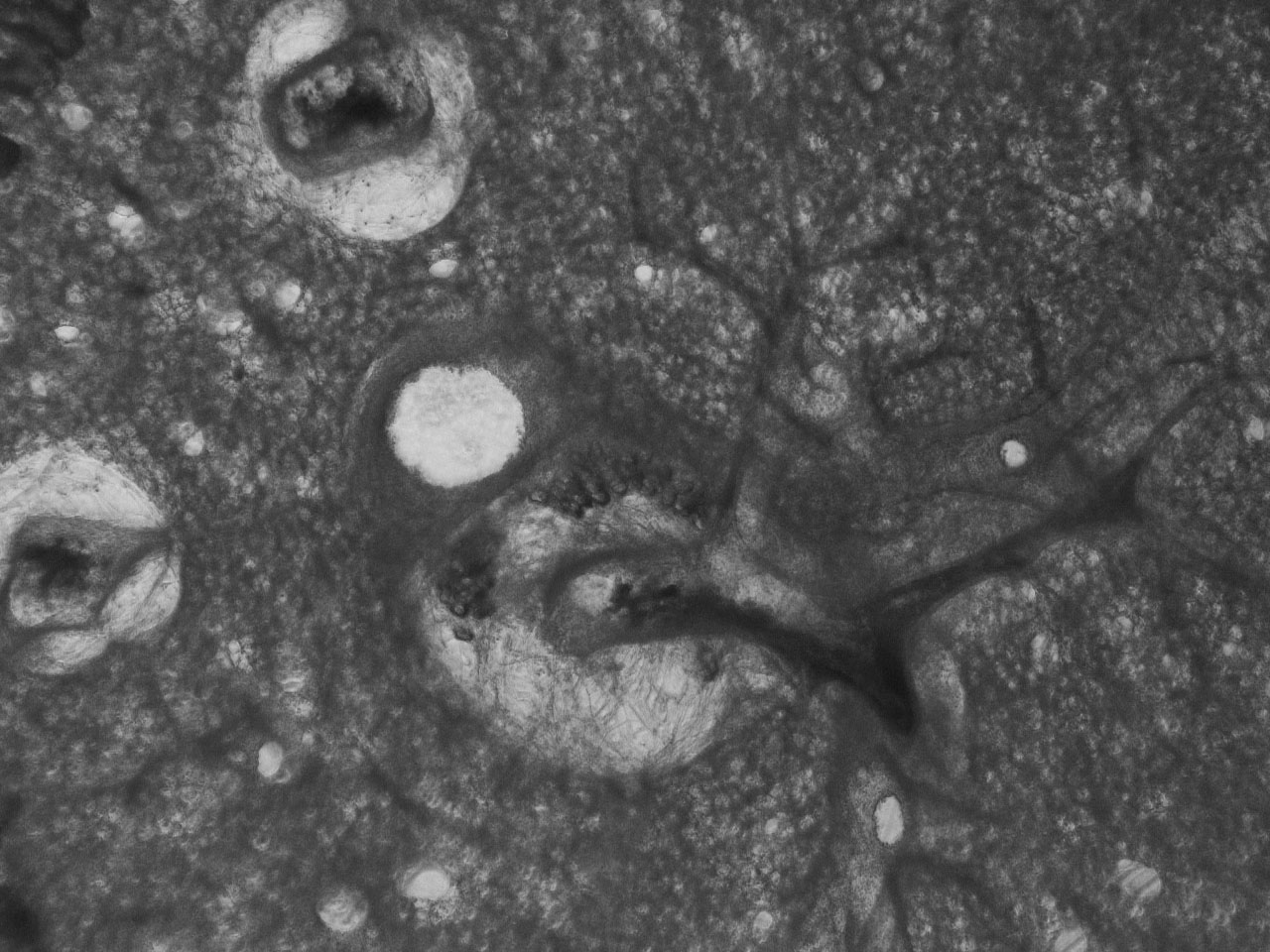

Supplement: Supplementary file 4 [file Presentation1.zip › StrainMap_package_21-08/Strain_Map/A1_c1_01_clean/frame0305.jpg]

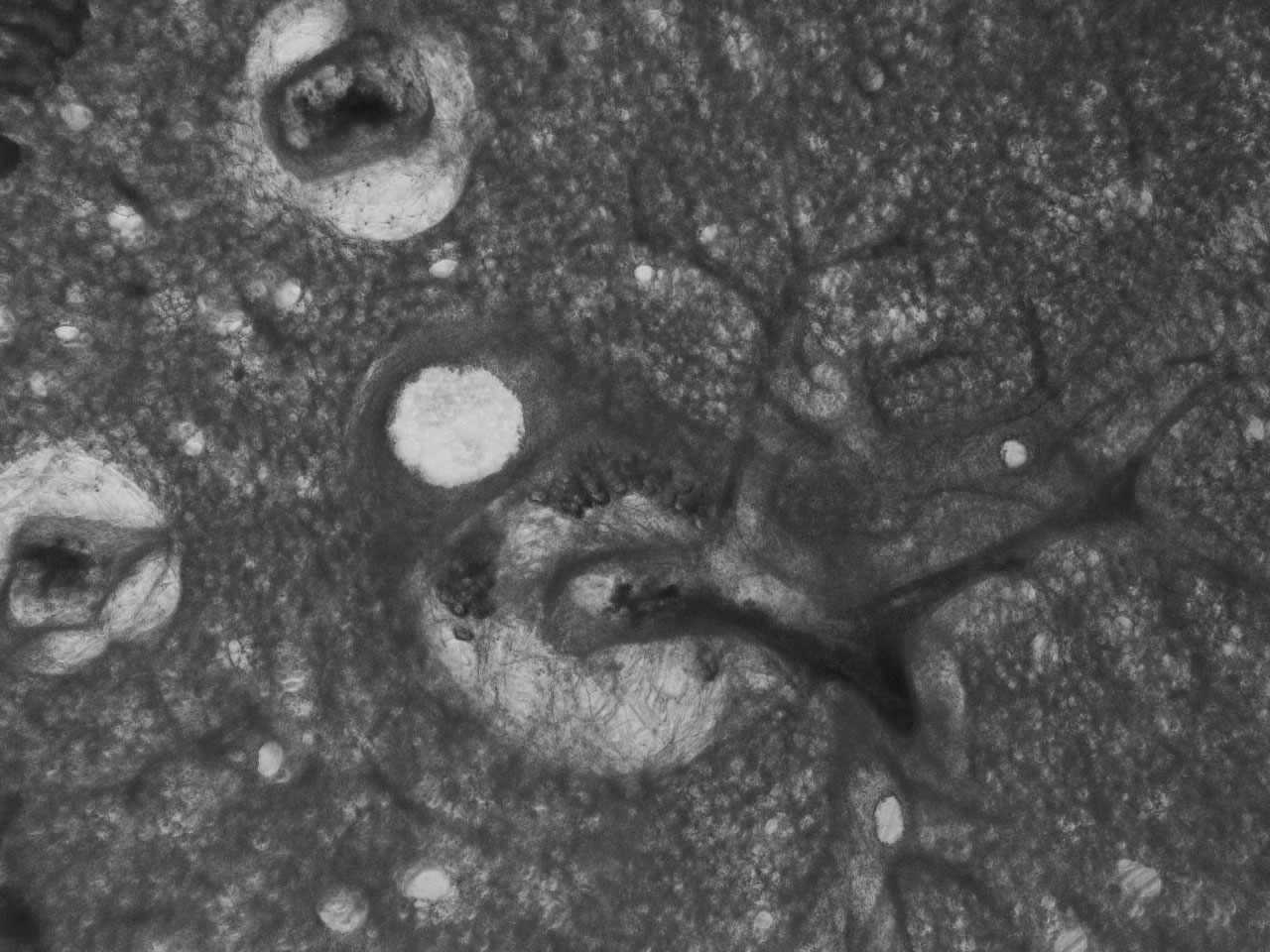

Supplement: Supplementary file 4 [file Presentation1.zip › StrainMap_package_21-08/Strain_Map/A1_c1_01_clean/frame0315.jpg]

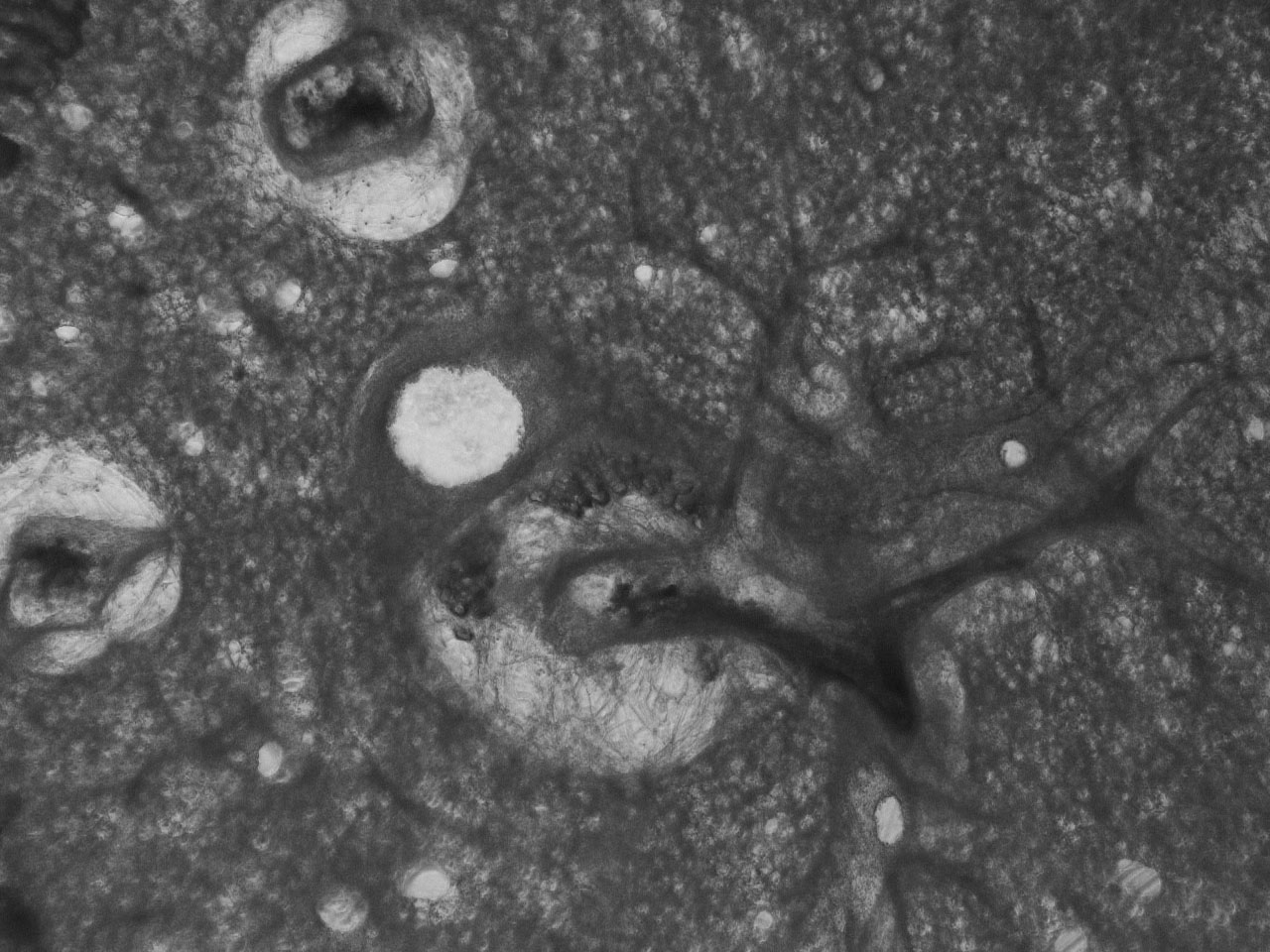

Supplement: Supplementary file 4 [file Presentation1.zip › StrainMap_package_21-08/Strain_Map/A1_c1_01_clean/frame0325.jpg]
