# Supplementary material for: Airway and Parenchymal Strains during Bronchoconstriction in the Precision Cut Lung Slice
Source: Front Physiol. 2016 Jul 21;7:309. doi: 10.3389/fphys.2016.00309 (PMC4989902; doi:10.3389/fphys.2016.00309)
Supplement: Supplementary file 5 [file Presentation2.pdf]

A-1 Appendix

A-1.1 Overview of image analysis

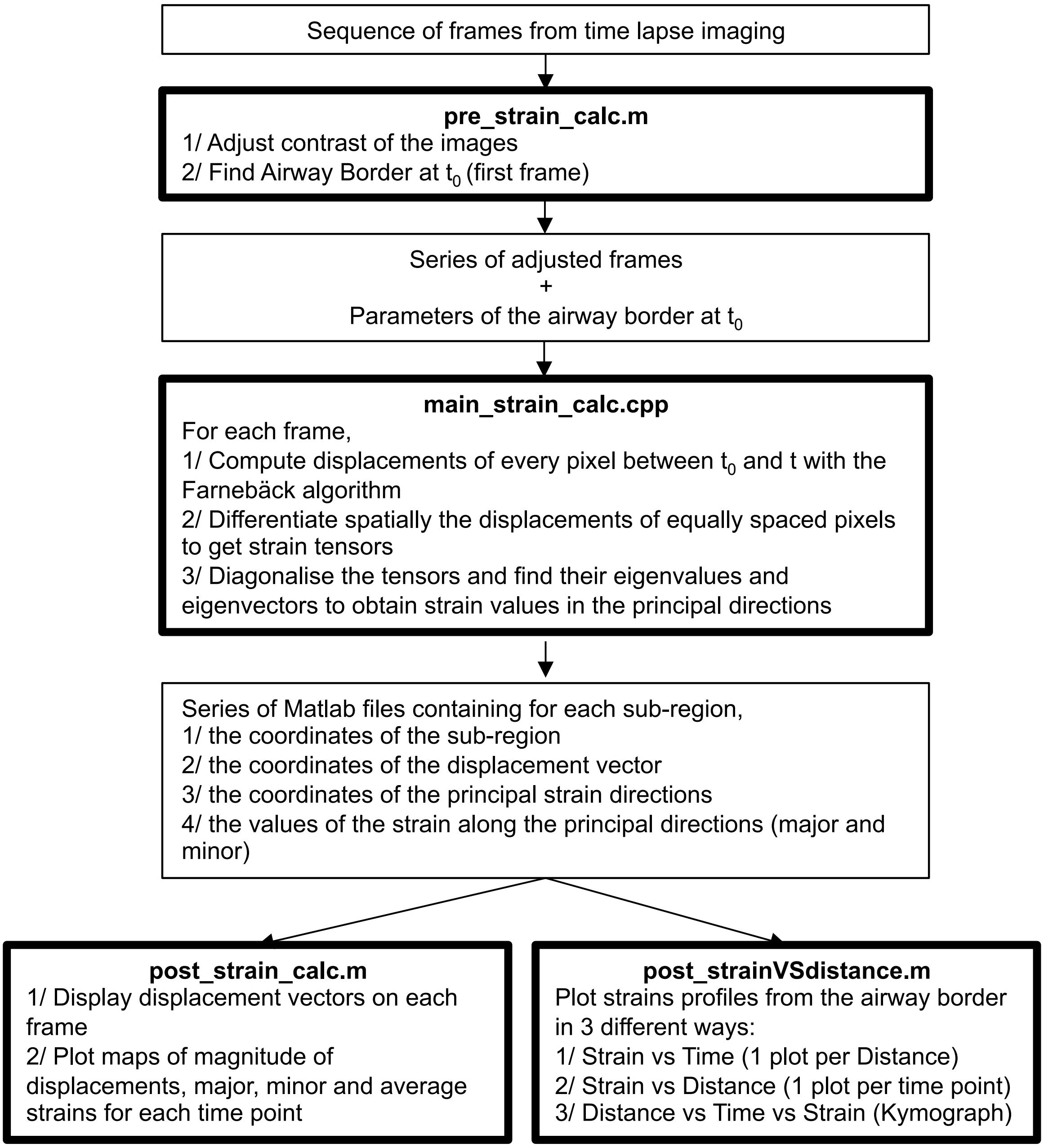

Figure A-1: Workflow showing steps required to compute global strain maps

A-1.2 Specific algorithms

A-1.2.1 Lumen edge detection

Having received the data in the form of videos, the free software Virtualdub (see www.virtualdub.org) is used to save the individual frames. The image processing toolbox in MATLAB is used

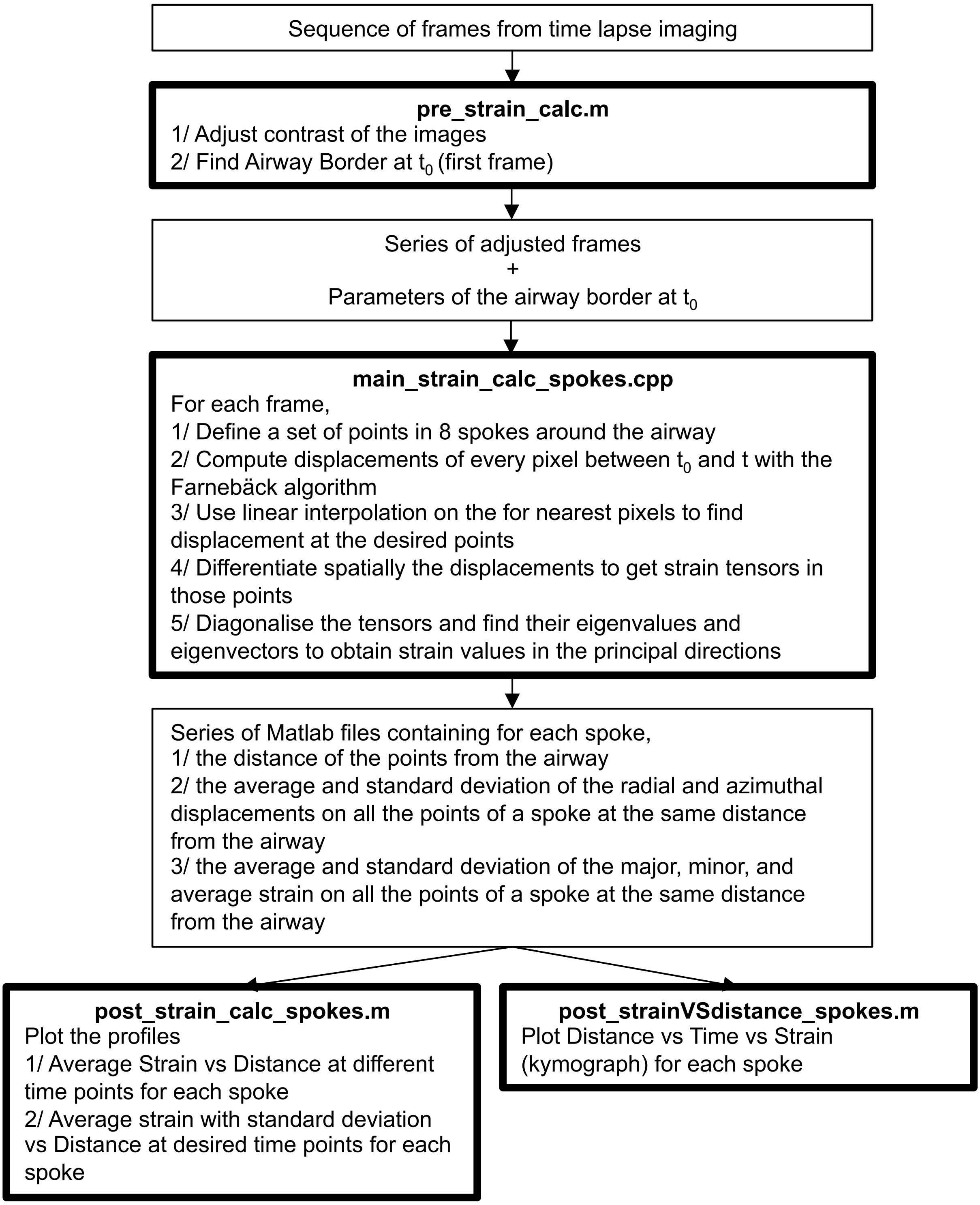

Figure A-2: Workflow showing steps required to compute strain maps along user-specified spokes.

to detect the edge, and the area, of the lumen in each frame. Depending on the lung slice being considered, specific MATLAB procedures are used in each of the frames; the image processing tools used in the procedures are detailed further, and the code is provided, in the Supplementary Material.

In each of the two methods we developed, an estimate for the edge of the lumen is determined, which can also be used to determine the lumen area either directly or by fitting an ellipse to the lumen. For the PCLS that had a clear contrast between the lumen and the airway wall we used the following sequence of tools: (i) `imread`, `imcrop`; (ii) `graythresh`, `im2bw`; (iii) `bwareaopen`; (iv) `imfill`; (v) `bwconncomp`, `regionprops`. From `regionprops` we obtain two estimates for the area of the lumen at each frame. An area can either be calculated within the region found (using `pwperim`) or an ellipse can be fitted to the region.

An alternative method for lumen edge detection (if the contrast between lumen and airway is not sufficient) uses the following sequence of MATLAB tools: (i) `imread`, `imcrop`, `rgb2gray`, `edge(I,'canny', thresh)`; (ii) `imdilate(I, [se90 se0])` (this closes the gaps between the edges that have been found); (iii) `imcomplement`, `bwareaopen`; (iv) `imfill`; (v) the area of the region detected, or that within a fitted ellipse, can be found using `bwconncomp` and `regionprops`.

A-1.2.2 Farnebäck method

The Farnebäck algorithm [12] as implemented in the opencv code cv::calcOpticalFlowFarneback[1], was used to calculate an estimate of the displacement vector between an initial and final image, for each of the pixels. In order to make the features in each of the images more prominent, prior to using the algorithm, the contrast of each image was increased. Each of the images was converted to greyscale and the range of the pixel intensities was stretched so that 1% of the pixels were saturated at the brightest value and 0.01% were saturated at the darkest value. The following MATLAB commands were used to do this: `imread`, `rgb2gray`, `stretchlim`, `imadjust` and `imwrite`. In regions where not enough features and/or insufficient contrast remained, thresholds were set to recalculate the displacements of the corresponding pixels by interpolation. This avoided the computation of spurious displacements. For any such points, the displacement was first set to `NAN` and then `griddata` in MATLAB was used with the `v4` method, to update the displacement at each of these points.

Following [12], we suppose that the two images are approximated by quadratic polynomial functions that describe the intensity of the pixels at position $\mathbf{x}$. The polynomials for the first and second image have the form

$$f_1(\mathbf{x}) = \mathbf{x}^T \mathsf{A}_1 \mathbf{x} + \mathbf{b}_1^T \mathbf{x} + c_1, \tag{A-1}$$

$$f_2(\mathbf{x}) = \mathbf{x}^T \mathsf{A}_2 \mathbf{x} + \mathbf{b}_2^T \mathbf{x} + c_2, \tag{A-2}$$

where A_1, A_2 are 2x2 matrices, $\mathbf{b}_1$, $\mathbf{b}_2$ are 2x1 vectors and c_1, c_2 are scalars. If the two images are only different by a rigid shift, $f_1(\mathbf{x}) = f_2(\mathbf{x} - \mathbf{d})$, where $\mathbf{d}$ is the displacement of the shift to be found. In this case

$$\mathsf{A}_2 = \mathsf{A}_1, \quad \mathbf{b}_2 = \mathbf{b}_1 - 2\mathsf{A}_1\mathbf{d}, \quad c_2 = \mathbf{d}^T \mathsf{A}_1 \mathbf{d} - \mathbf{b}_1^T \mathbf{d} + c_1, \tag{A-3}$$

[1] http://opencv.willowgarage.com/documentation/cpp/motion_analysis_and_object_tracking.html

where, assuming that A_1 is non-singular, $\mathbf{d}$ is given by

$$\mathbf{d} = -\mathsf{A}_1 \frac{\mathbf{b}_2 - \mathbf{b}_1}{2}. \tag{A-4}$$

In general it is more complicated than this, since the displacement is spatially dependent and will also involve rotation and stretching. Rather than finding intensity polynomial functions over the whole region, local polynomial functions are found over a small neighbourhood surrounding each of the pixels. A spatially-dependent displacement $\mathbf{d}(\mathbf{x})$ is found using the local polynomials of the two images. If however, the displacements are large, the comparison of local polynomials in the two images may be insufficient, since the displaced point may not be located within the local neighbourhood of the initial position used to form the polynomial. In this case a false displacement will be found. The algorithm is able to overcome this problem by using *a priori* knowledge. Given an *a priori* displacement $\tilde{\mathbf{d}}(\mathbf{x})$, a relative displacement can be found using $f_1(\mathbf{x})$ and $f_2(\tilde{\mathbf{x}})$, where $\tilde{\mathbf{x}} = \mathbf{x} + \tilde{\mathbf{d}}(\mathbf{x})$. $\tilde{\mathbf{d}}(\mathbf{x})$ (which is measured relative to pixel width) is rounded to the nearest integer, so that the polynomial in the second image is centred on a pixel. Now in general, $\mathsf{A}_1 \neq \mathsf{A}_2$, but introducing

$$\mathsf{A}(\mathbf{x}) = \frac{\mathsf{A}_1(\mathbf{x}) + \mathsf{A}_2(\tilde{\mathbf{x}})}{2}, \quad \Delta\mathbf{b}(\mathbf{x}) = -\frac{1}{2}(\mathbf{b}_2(\tilde{\mathbf{x}}) - \mathbf{b}_1(\mathbf{x})) + \mathsf{A}(\mathbf{x})\tilde{\mathbf{d}}(\mathbf{x}), \tag{A-5}$$

the constraint for the updated displacement is

$$\mathsf{A}(x)\mathbf{d}(\mathbf{x}) = \Delta\mathbf{b}(\mathbf{x}). \tag{A-6}$$

In practice the displacement field that is found will be too noisy. The algorithm overcomes this by assuming that the displacement field is only slowly varying. In this case, for each pixel, it is possible to solve with an appropriate weight function $w(\Delta\mathbf{x})$ over a region Ω, which forms a square of pixels around the current pixel. This results in having to find the minimum of

$$\sum_{\Delta\mathbf{x}\in\Omega} w(\Delta\mathbf{x}) \|\mathsf{A}(\mathbf{x}+\Delta\mathbf{x})\mathbf{d}(\mathbf{x}) - \mathbf{b}(\mathbf{x}+\Delta\mathbf{x})\|^2. \tag{A-7}$$

Increasing the size of Ω results in smoother displacement fields.

In reality an initial guess of the displacements was generally not available, in which case an iterative system could be used. The initial iterations were used to find an approximation of the displacements, with further iterations improving the approximation. If the displacement between the two frames was large, the initial size of the neighbourhood, used to fit the polynomials $f_1(\mathbf{x})$ and $f_2(\mathbf{x})$, was increased, in order to find a rough but reasonable displacement estimation. This displacement was then used as a priori displacement, which was improved in two ways. Further iterations were carried out with the same neighbourhood size, or in order to find more of the local features of the displacement field, the size of the neighbourhood of the pixels used to find the polynomials was reduced.

When implementing the OpenCV code, unless otherwise stated we used three sizes of square neighbourhoods to form the pixel intensity polynomials. For each subsequent square size we halved the length of sides and iterated three times for each size. Using the suggested values in the OpenCV documentation, we used a final side length of 5 pixels and set the standard deviation of the Gaussian, used to smooth derivatives in order to form the polynomials $f_1(\mathbf{x})$ and $f_2(\mathbf{x})$, to 1.1 pixels. However this had to be modified to 7 and 1.5 (still following the OpenCV documentation) to track large displacements on high resolution pictures (1280x960 pixels). We found that introducing additional larger squares

did not improve the results.

A-1.2.3 Determining displacements for points along normal vectors

An alternative to calculating the entire displacement field was to find the displacement at selected points. By selecting points along a normal vector to the lumen, it was easier to quantify displacement as a function of radius (or distance from the lumen). By doing this at various points around the lumen, the displacement-radius relationship could be compared. We first fitted an ellipse to the lumen at the start of the contraction (details in S. in Supp. Mat). We then split the airway into eight sections, within each of which we selected points along normal vectors starting at seven points on the lumen boundary. We found displacements in the tangential and normal directions at each point and averaged these values within each of the sections for each radial position, in order to remove small errors.

We begin by fitting an ellipse to the lumen at the start of the contraction, using the techniques described in section A-1.2.1. In parametric form an ellipse centred at (x_0, y_0), with major and minor axis of length $2a$ and $2b$ and angle α between the x axis and the major axis, has coordinates

$$x = x_0 + a\cos t\cos\alpha - b\sin t\sin\alpha, \tag{A-8a}$$

$$y = y_0 + a\cos t\sin\alpha + b\sin t\cos\alpha, \tag{A-8b}$$

where $t \in [0, 2\pi)$ is the parametric parameter. The unit vectors in the tangential and normal directions are

$$\mathbf{t} = \frac{(-a\sin t\cos\alpha - b\cos t\sin\alpha, -a\sin t\sin\alpha + b\cos t\cos\alpha)}{\sqrt{a^2\sin^2 t + b^2\cos^2 t}}, \tag{A-9a}$$

$$\mathbf{n} = \frac{(-a\sin t\sin\alpha + b\cos t\cos\alpha, a\sin t\cos\alpha + b\cos t\sin\alpha)}{\sqrt{a^2\sin^2 t + b^2\cos^2 t}}. \tag{A-9b}$$

Eight groups of seven points are chosen on the ellipse with the coordinates

$$(x_e, y_e) = (x(t), y(t)), \quad t = \alpha + m\pi/4 + n\pi/180, \tag{A-10}$$

with $m = 0, 1, .., 7$ and $n = -3, -2, .., 3$. If the ellipse was a perfect fit to the lumen each of the points would be located at the lumen boundary. In practice the lumen is not so regular, so the choice of points given in (A-10) may need to be slightly altered. Where required, we slightly inflate or deflate the ellipse, while fixing the ratio of a and b, in order to select a point on the boundary. For each of the new points we find the normal to the lumen and select further points spaced by k pixels in the direction of the normal. This yields the points

$$(x, y) = (x_e, y_e) + \mathbf{n}(t)ks, \quad s = 0, 1, ... \tag{A-11}$$

An illustration of how one line of points are chosen and an example of the points chosen is shown in Fig. A-3. Since in general the coordinates are not integer values, bilinear interpolation of the four nearest pixels is used to find the displacement. The radial and azimuthal components of the displacements are found by taking the dot product of the displacement with the unit normal and tangent vectors.

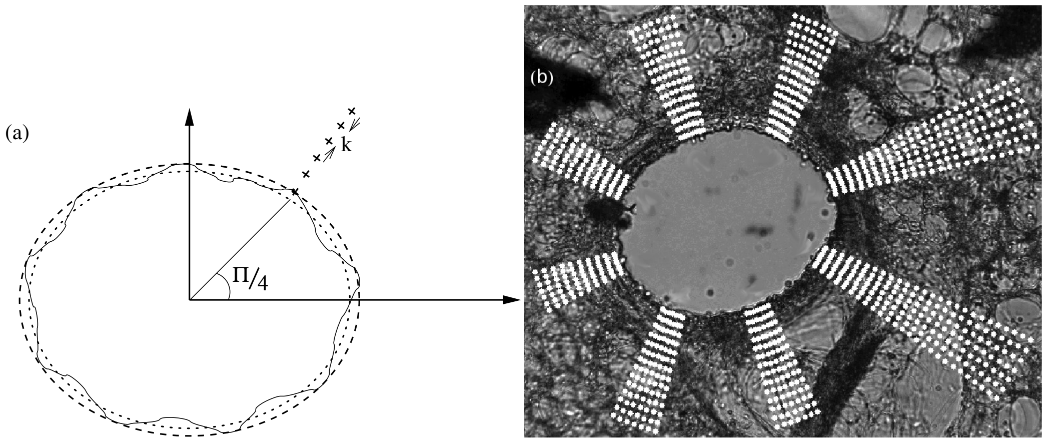

Figure A-3: (a) An ellipse (dotted line) is fitted to the edge of the lumen (solid line). However, a particular point on the lumen boundary may not lay on this ellipse, in which case we inflate (or deflate) the ellipse accordingly so that the point lies on the adjusted ellipse (dashed line). The normal to the adjusted ellipse is found and points are chosen at intervals of k pixels. (b) An example of the initial set of points (white dots) superimposed on an image of a lung slice.

A-1.3 Lumen area image processing tools

The following tools are used in at least one of the procedures (`I` is used to represent the latest version of the image):

- `imread(N)`: used to load up the image from a file N;
- `imcrop(I, rect)`: used to take a rectangular section (`rect` specifies the coordinates of the section) of the image around the airway;
- `level = graythresh(I)`: computes a threshold of the image, which can be used to produce a binary image;
- `im2bw(I,level)`: changes the image to a binary image;
- `rgb2gray(I)`: converts an image to greyscale;
- `bwareaopen(I, numpixel, 4)`: removes from the binary image any groups of less than numpixel of connected pixels (`4` means that two pixels are only connected if they share an edge);
- `imfill(I,'holes')`: fills in any small holes in an object;
- `edge(I,'canny', thresh)`: detects edges using the Canny method (Edges are found by searching for local maxima of the gradient of `I`. The derivative of a Gaussian filter is used to calculate the gradient. The method uses two thresholds, to detect strong and weak edges, only including the weak edges if they are connected to strong edges.);
- `imdilate(I, [strel('line', 3, 90) strel('line', 3, 0)])`: lines are dilated by three pixel each way in the horizontal and vertical directions;
- `imcomplement(I)`: the binary image is inverted;

- `cc = bwconncomp(I,4)`: the binary image is split up into sections depending on the connectivity of the pixels (the resulting number of objects can be obtained using `cc.NumObjects`);
- `imagedata = regionprops(cc, 'Area', 'Centroid', 'Orientation', 'MajorAxisLength', 'MinorAxisLength')`: finds the area and centroid of each object and the length of the major and minor axis and the orientation of the major axis to the horizontal of an ellipse that has the same second-moments as the object;
- `BWoutline = bwperim(I);Segout = I2;Segout(BWoutline) = 255`: draws the outline found onto the original image.
