# Supplementary material for: Airway and Parenchymal Strains during Bronchoconstriction in the Precision Cut Lung Slice
Source: Front Physiol. 2016 Jul 21;7:309. doi: 10.3389/fphys.2016.00309 (PMC4989902; doi:10.3389/fphys.2016.00309)
Supplement: Supplementary file 6 [file DataSheet1.PDF]

Supplementary Material: Airway and parenchymal strains during bronchoconstriction in the precision cut lung slice

J.E. Hiorns, C.M. Bidan, O.E. Jensen, R. Gosens, L.E.M. Kistemaker, J.J. Fredberg, J.P. Butler, R. Krishnan, B. S. Brook*.
*Correspondence:
Bindi Brook:
bindi.brook@nottingham.ac.uk

1 SUPPLEMENTARY VIDEOS

Video M1: Global analysis of the deformations of the mouse lung slice. Displacement vectors of small regions (7x7pixels) of the slice computed between the reference (at t_0) and images at increasing time, shown as they evolve over time during the contraction and relaxation phase.

Video M2: Global analysis of the deformations of the mouse lung slice. Displacements of small regions (7x7pixels) of the slice computed between the reference (at t_0) and images at increasing time, shown as a colour map as they evolve over time during the contraction and relaxation phases.

Video M3: Global analysis of the deformations of the mouse lung slice. Radial (major) and circumferential (minor) strains calculated by spatial derivation of the displacements and displayed as maps over the whole field as they evolve over time during the contraction and relaxation phases.

2 SUPPLEMENTARY CODE

Details of the code and algorithms used are described in the main text and the Appendix. Here we have made available the code in C++ and MATLAB scripts within the zipped archive "StrainMap_package_Frontiers.zip". All instructions for use and for trouble shooting are included, and specific scripts mentioned in the manuscript can be found in Strain_Map > A1_c1_01_clean > Strain Files .

3 SUPPLEMENTARY FIGURES

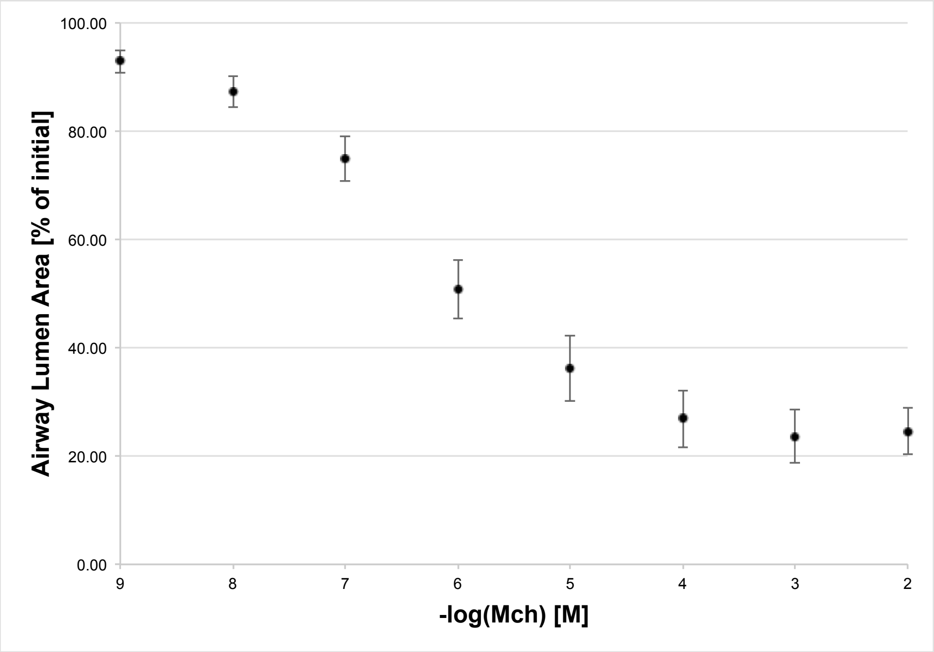

Figure S1. Dose response curve showing mean airway lumen area as a percentage of baseline area on application of increasing doses of MCh. $n = 8$ with 3-7 slices per mouse. Bars show standard error of the mean.

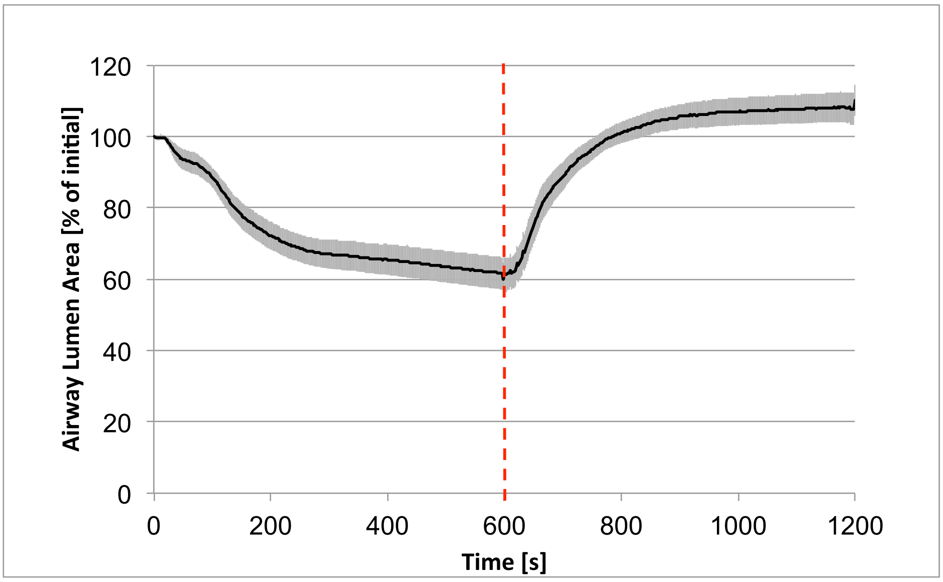

Figure S2. Temporal evolution of mean airway lumen area as a percentage of baseline area on application of methacholine (MCh; 10^{-4}M) at $t_0 = 0$ s, and then addition of the bitter taste receptor agonist chloroquine (ChQ; 10^{-3}M) to induce relaxation at $t_1 = 600$ s (in the presence of MCh); $n = 8$ with 3-7 slices per mouse. Bars show standard error of the mean.

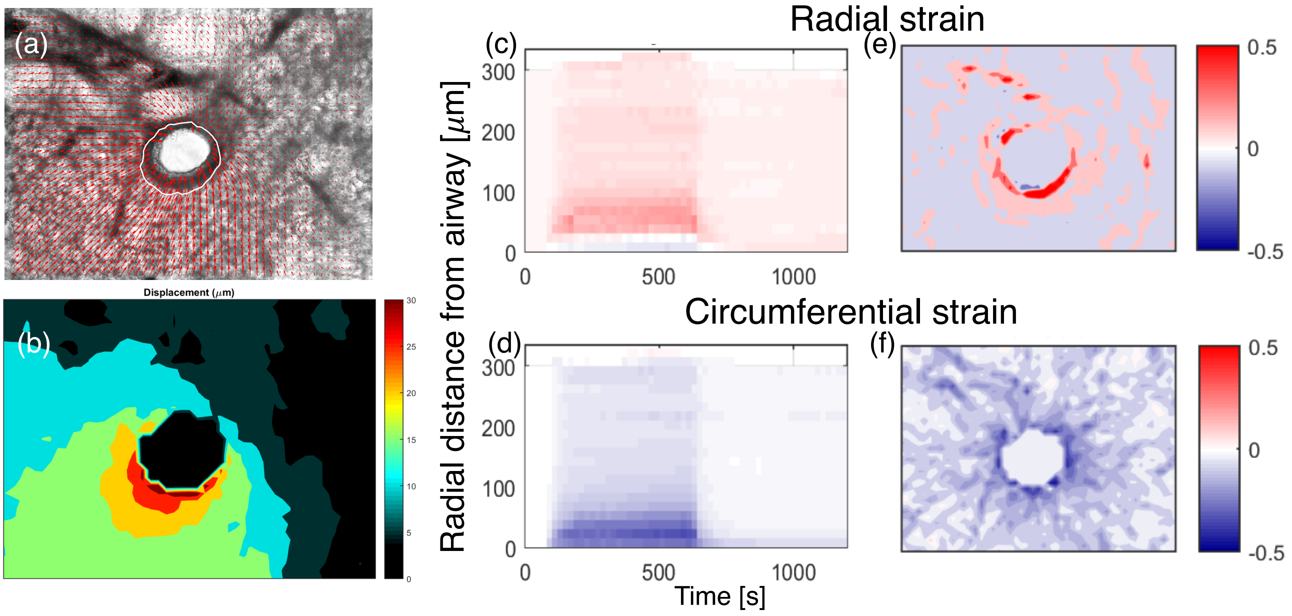

Figure S3. Global analysis of the deformations of additional mouse lung slice no. 1. Displacement (a) vectors and (b) magnitude of small regions (7x7pixels) of the slice computed between the reference (at t_0) and the contracted state (at t_1). The boundary of the airway before contraction is represented as a solid white line. (c-f) Radial (top panel) and circumferential (bottom panel) strains calculated by spatial derivation of the displacements and displayed as functions of time in adapted kymographs (c,d) and as maps over the whole field (e,f). Magnitude of strain is indicated by the colorbars on the right.

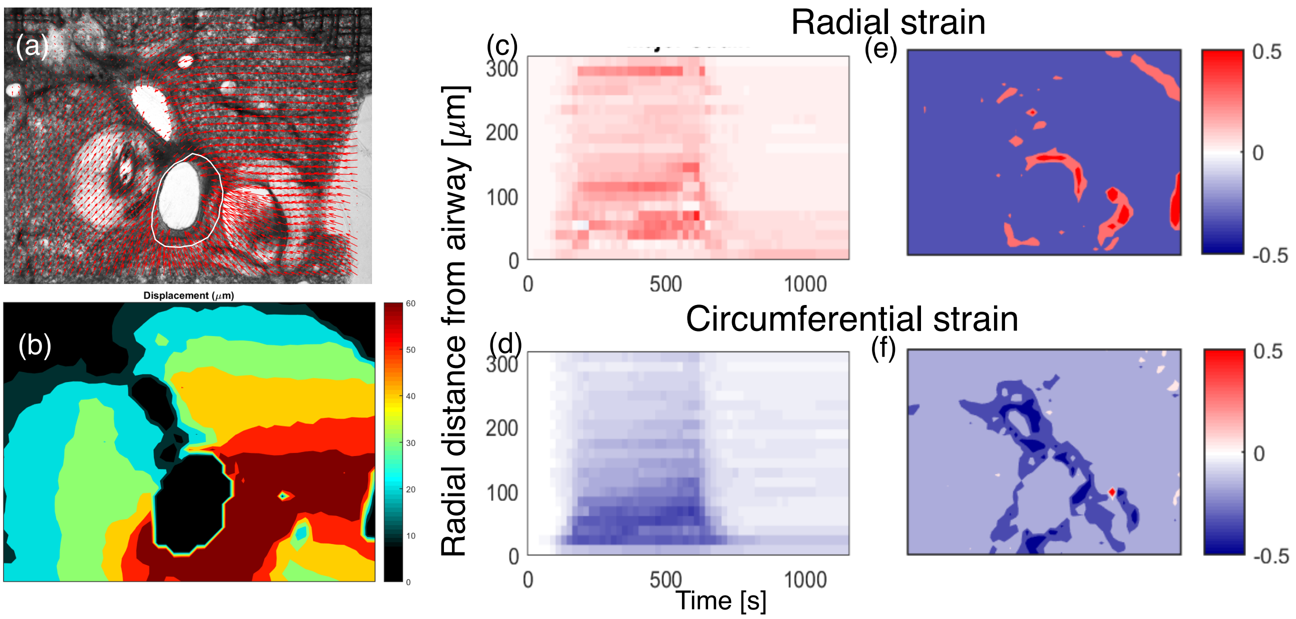

Figure S4. Global analysis of the deformations of additional mouse lung slice no. 2. Displacement (a) vectors and (b) magnitude of small regions (7x7pixels) of the slice computed between the reference (at t_0) and the contracted state (at t_1). The boundary of the airway before contraction is represented as a solid white line. (c-f) Radial (top panel) and circumferential (bottom panel) strains calculated by spatial derivation of the displacements and displayed as functions of time in adapted kymographs (c,d) and as maps over the whole field (e,f). Magnitude of strain is indicated by the colorbars on the right.

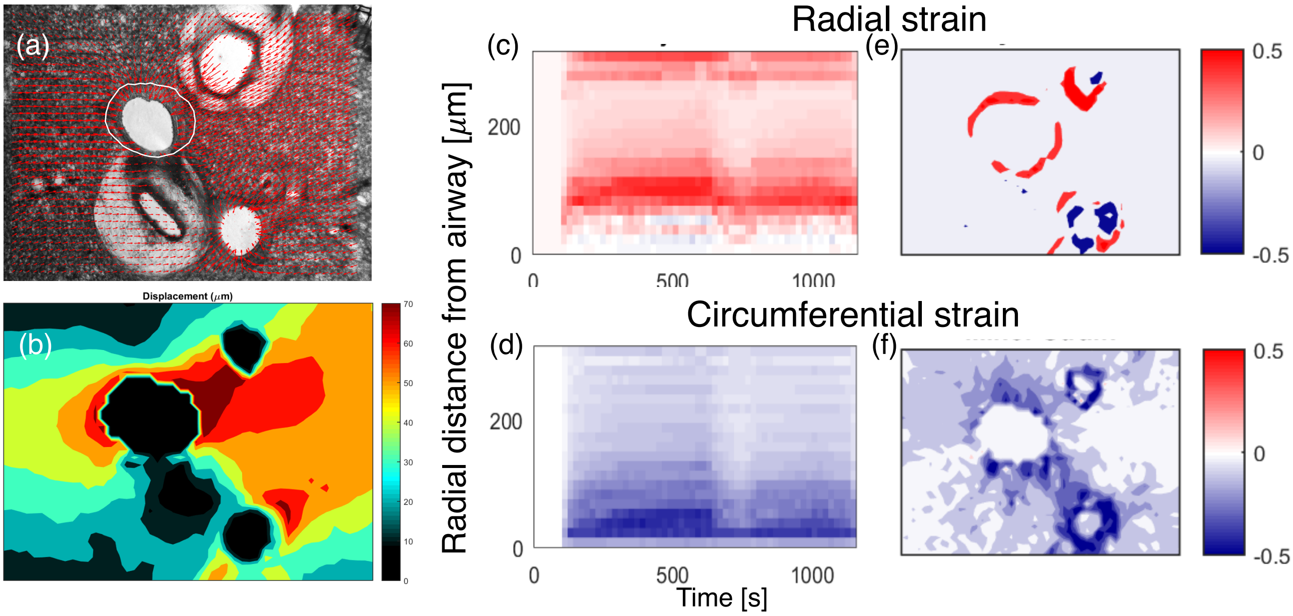

Figure S5. Global analysis of the deformations of additional mouse lung slice no. 3. Displacement (a) vectors and (b) magnitude of small regions (7x7pixels) of the slice computed between the reference (at t_0) and the contracted state (at t_1). The boundary of the airway before contraction is represented as a solid white line. (c-f) Radial (top panel) and circumferential (bottom panel) strains calculated by spatial derivation of the displacements and displayed as functions of time in adapted kymographs (c,d) and as maps over the whole field (e,f). Magnitude of strain is indicated by the colorbars on the right.
