# Supplementary material for: Airway and Parenchymal Strains during Bronchoconstriction in the Precision Cut Lung Slice
Source: Front Physiol. 2016 Jul 21;7:309. doi: 10.3389/fphys.2016.00309 (PMC4989902; doi:10.3389/fphys.2016.00309)
Supplement: Supplementary file 4 [file Presentation1.zip › StrainMap_package_21-08/Strain_Map/File_Notes_Jonathan.doc]

File Notes

There are five files included. Three of them are Matlab files and two are C++ files. The Matlab files are used for pre and post processing.

pre_strain_calc.m is used to adjust the contrast in the initial images and to find a list of all of the frame numbers that will be used. An ellipse is also fitted to the lumen of the first image, which is used if the spokes method is subsequently used.

I made use of Code::blocks to run the C++ code and OpenCV 2.2. The following website was used to get it working: <http://conanhung.wordpress.com/2012/05/23/opencv-2-2c-codeblocks-and-mingw-got-it-working-on-windows/>

There are two options:

1. a grid of points are chosen and the displacements and strains are found at each of the points

The C++ file main_strain_calc.cpp is used and the matlab file post_strain_calc.m is used to	set the displacement and strain to zero within the lumen and then plot and save figures

2. the slice is split into 8 regions within which points are chosen along lines radiating out from the lumen. Averages and standard deviations of several such lines in each of the sets can be found in each of the regions. Again displacements and strains can be found

The C++ file main_strain_calc_spokes.cpp is used and the matlab file post_strain_calc_spokes.m is used to plot figures
